# Ancient DNA from the Green Sahara reveals ancestral North African lineage

**DOI:** 10.1038/s41586-025-08793-7

**Published:** 2025-04-02

**Authors:** Nada Salem, Marieke S. van de Loosdrecht, Arev Pelin Sümer, Stefania Vai, Alexander Hübner, Benjamin Peter, Raffaela A. Bianco, Martina Lari, Alessandra Modi, Mohamed Faraj Mohamed Al-Faloos, Mustafa Turjman, Abdeljalil Bouzouggar, Mary Anne Tafuri, Giorgio Manzi, Rocco Rotunno, Kay Prüfer, Harald Ringbauer, David Caramelli, Savino di Lernia, Johannes Krause

**Affiliations:** 1 Max Planck Institute for Evolutionary Anthropology, Leipzig, Germany; 2 Max Planck−Harvard Research Center for the Archaeoscience of the Ancient Mediterranean (MHAAM), Leipzig, Germany; 3 Biosystematics Group, Wageningen University, Wageningen, The Netherlands; 4 Department of Biology, University of Florence, Florence, Italy; 5 The Department of Antiquities (DOA), Tripoli, Libya; 6 Institut National des Sciences de l’Archéologie et du Patrimoine, Origin and Evolution of Homo sapiens Cultures, Rabat, Morocco; 7 Department of Environmental Biology, Sapienza University of Rome, Rome, Italy; 8 Department of Ancient World Studies, Sapienza University of Rome, Rome, Italy; 9 School of Geography, Archaeology and Environmental Studies (GAES), University of Witwatersrand, Johannesburg, South Africa

**Keywords:** Genomics, Population genetics, Archaeology, Evolutionary genetics, Anthropology

## Abstract

Although it is one of the most arid regions today, the Sahara Desert was a green savannah during the African Humid Period (AHP) between 14,500 and 5,000 years before present, with water bodies promoting human occupation and the spread of pastoralism in the middle Holocene epoch^1^. DNA rarely preserves well in this region, limiting knowledge of the Sahara’s genetic history and demographic past. Here we report ancient genomic data from the Central Sahara, obtained from two approximately 7,000-year-old Pastoral Neolithic female individuals buried in the Takarkori rock shelter in southwestern Libya. The majority of Takarkori individuals’ ancestry stems from a previously unknown North African genetic lineage that diverged from sub-Saharan African lineages around the same time as present-day humans outside Africa and remained isolated throughout most of its existence. Both Takarkori individuals are closely related to ancestry first documented in 15,000-year-old foragers from Taforalt Cave, Morocco^2^, associated with the Iberomaurusian lithic industry and predating the AHP. Takarkori and Iberomaurusian-associated individuals are equally distantly related to sub-Saharan lineages, suggesting limited gene flow from sub-Saharan to Northern Africa during the AHP. In contrast to Taforalt individuals, who have half the Neanderthal admixture of non-Africans, Takarkori shows ten times less Neanderthal ancestry than Levantine farmers, yet significantly more than contemporary sub-Saharan genomes. Our findings suggest that pastoralism spread through cultural diffusion into a deeply divergent, isolated North African lineage that had probably been widespread in Northern Africa during the late Pleistocene epoch.

## Abstract (web summary)

Pastoralism spread through cultural diffusion into the Green Sahara, where an isolated, distinct North African ancestry persisted.

## Main

**Fig. 1:**
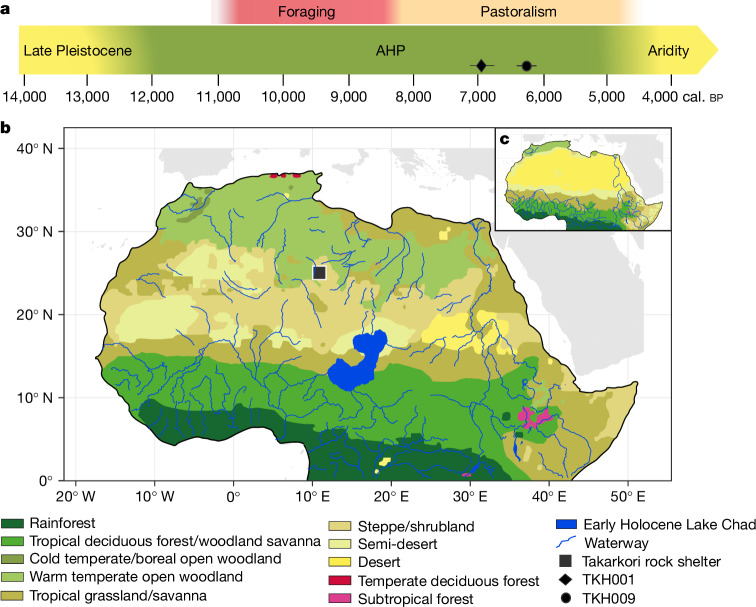
Chronology of the ecozones and subsistence strategies in the broader Sahara region. **a**, Timeline of climate phases and subsistence strategies during the late Pleistocene and the Holocene in North-East Africa and Central Sahara. The radiocarbon dates for both Takarkori individuals are given by the black diamond and circle. **b**,**c**, The distribution of ecozones in Northern Africa in the Green Sahara period during the early Holocene 9,000 years ago (**b**) and in recent times (1901–1930) (**c**) using the dynamic vegetation model Carbon Assimilation in the Biosphere (CARAIB). The location of the Takarkori rock shelter site is marked with a black square. The maps are adapted from refs. ^20^ and ^60^ under a Creative Commons licence CC BY 4.0.

Following the last glacial period, a climatic transformation in the Sahara desert led to the AHP, which peaked around 11,000 to 5,000 years ago^3,4^. During this period of increased humidity, the region transformed into a ‘Green Sahara’ with savanna-like landscapes, varying tree cover, permanent lakes and extensive river systems^5^ (Fig. 1). Evidence from ancient lake deposits, pollen samples and archaeological artifacts confirm human presence, hunting, herding and resource gathering in the currently arid desert region^1,6,7^. However, despite this rich history, much about the genetic history of the human population of the Green Sahara remains unclear due to limited DNA preservation under the current climatic conditions. Ancient DNA data from northwestern Africa points to a stable and isolated genetic population from at least 15,000 to 7,500 years ago^2,8^. This stability was disrupted by the arrival of early farming groups from southwestern Europe between 7,500 and 5,700 years ago who marked the beginning of the Neolithic in the Maghreb by introducing farming practices to the local foragers^9^. The earliest herders with their livestock entered Africa probably along the Sinai and the Red Sea routes, after which they rapidly spread into northeastern Africa and reached the Central Sahara around 8,300 years ago^10^. By 6,400 years ago, further gene flow occurred with the appearance of ancestry associated with Neolithic groups from the Levant, whose archaeological signatures are visible in the Eastern Sahara^9,11,12^. A previous study^13^ analysed mitochondrial DNA (mtDNA) from individuals recovered from the Takarkori rock shelter in the Tadrart Acacus Mountains of southwestern Libya—the same individuals examined in this study—providing the first ancient DNA from pastoralists of the Green Sahara. However, non-recombining and therefore effectively single genetic loci like mtDNA have much less statistical power to reveal population dynamics than genome-wide autosomal data. Their origins and whether the arrival of pastoralism into the Green Sahara was linked to the movement of peoples from the Levant or rather cultural diffusion remain a matter of debate^10,14^.

Here we present first genome-wide data obtained from the same two approximately 7,000-year-old Saharan herders, recovered from the Takarkori rock shelter in the Central Sahara (Supplementary Figs. 1.2–1.5), a site that has yielded an exceptional wealth of data and material remains^15–19^. Our findings show that these individuals predominantly carry a previously unknown ancestral North African lineage that lacks the Neanderthal admixture typically found outside Africa and appears to have remained largely isolated, with the notable exception of small traces of Levantine admixture. These results support that pastoralism in the Sahara was established through cultural diffusion^10^ rather than significant human gene flow. Furthermore, the Takarkori individuals exhibit a close genetic affinity to Northwestern African foragers but no substantial ties with sub-Saharan African lineages, implying no detectable genetic exchange across the Green Sahara during the AHP from sub-Saharan to northern Africa. For a non-peer-reviewed Arabic summary of the article, see Supplementary Note 3.

## Takarkori: a window into the Green Sahara

The Takarkori rock shelter—situated in southwestern Libya’s Tadrart Acacus Mountains—offers a remarkable glimpse into the Sahara’s greener past^15,20^. The excavations at this archaeological site revealed a timeline of human settlement from Late Acacus hunter-gatherer-fishers from around 10,200 calibrated years before the present (cal. bp) to a long Pastoral Neolithic occupation, dated from approximately 8,300 to 4,200  cal. bp^10,21^. Data from these latter periods traces the sociocultural trajectories of Neolithic herding societies in Central Sahara, from early livestock introduction to the development of a full pastoral economy characterized by transhumance and the use of secondary product^22,23^ (Supplementary Note 1). Within the deepest recess of the rock shelter, 15 human burials were unearthed, pertaining to a timeframe between approximately 8,900 and 4,800 cal. bp^22^, with the majority dating back to the Early (8,300–7,300 cal. bp) and Middle (7,100–5,600 cal. bp) Pastoral period^22^. Strontium isotope analysis on the remains, primarily of women of reproductive age, children and juveniles, indicated a local geographical origin^24^. Two naturally mummified adult female individuals, attributed to the Middle Pastoral Period, were selected for our DNA analysis. These individuals were directly radiocarbon dated to 7,158–6,796 and 6,555–6,281 cal. bp (95.4% probability), respectively^13^ (Supplementary Note 1).

## Genomic analyses

We extracted DNA from the powdered tooth root from individual TKH001 and from two fibula fragments from TKH009. Given their extremely low endogenous human DNA content (0.085–1.363%; Supplementary Table 2.1), we opted for a DNA capture approach to cost-effectively retrieve informative single-nucleotide polymorphisms (SNPs) for autosomal analysis. Sampling of other skeletal elements in the future may provide DNA extracts with higher percentage of endogenous DNA that allow for whole-genome shotgun sequencing. Using dedicated ancient DNA protocols (Methods), we prepared DNA libraries and enriched them through a DNA hybridization approach with the Twist Ancient DNA panel^25^ targeting 1.4 million SNPs for TKH001 and the 1240K panel^26^ targeting 1.2 million SNPs for TKH009. Despite challenging preservation conditions, sequencing yielded 881,765 SNPs for TKH001 and 23,317 SNPs for TKH009 (Supplementary Table 2.4). For TKH001, we conducted additional enrichment for 1.7 million SNPs, targeting sites informative about Neanderthal and Denisovan admixture (Archaic Admixture SNP capture panel)^27^. DNA sequences from both Takarkori individuals had a post-mortem degradation pattern typical of ancient DNA and low contamination estimates (Supplementary Figs. 2.1, 2.2 and Supplementary Table 2.3).

**Fig. 2:**
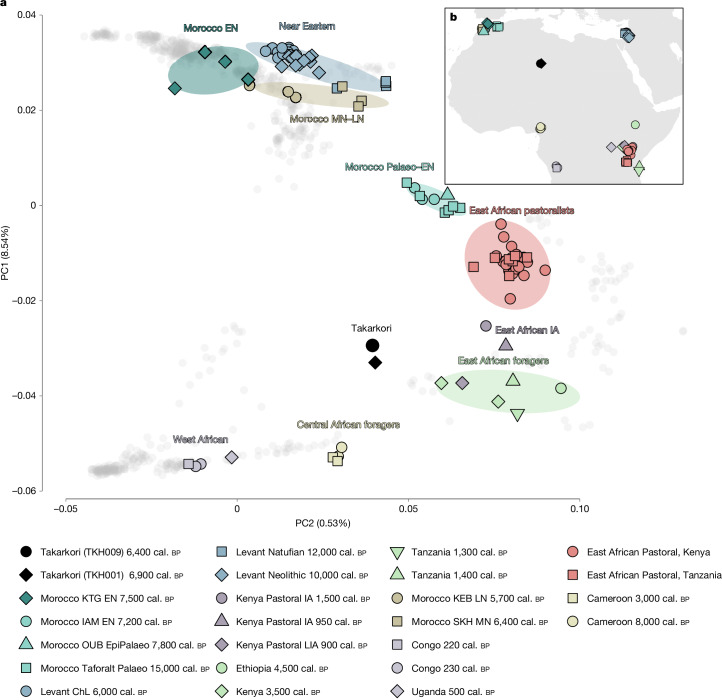
PCA calculated on present-day individuals from Africa, the Near East and Southern Europe, and the geographical locations of these individuals. **a**, PCA with projecting key ancient groups from the region (Supplementary Data 3). **b**, The geographical locations of ancient genomes from Africa and the Near East included in our analysis. ChL, Chalcolithic; EN, Early Neolithic; EpiPalaeo, Epipalaeolithic; IA, Iron Age; IAM, Ifri n’Amr o’Moussa; KEB, Kehf el Baroud; KTG, Kaf Taht el-Ghar; LIA, Late Iron Age; LN, Late Neolithic; MN, Middle Neolithic; OUB, Ifri Ouberrid; Palaeo, Palaeolithic; SKH, Skhirat-Rouazi.

**Extended Data Fig. 1:**
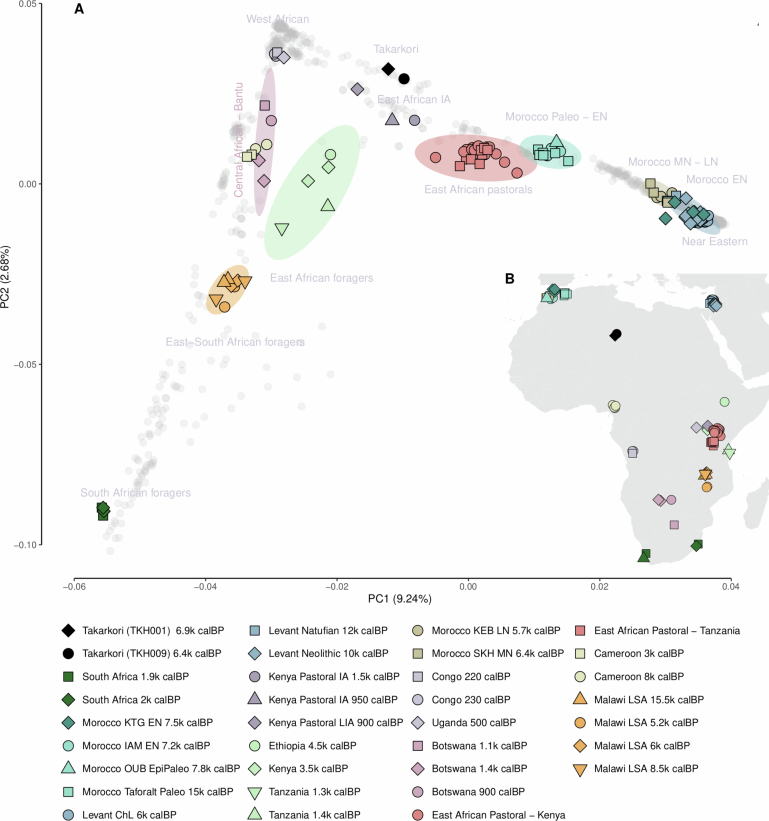
PCA calculated on present-day individuals from the whole of the African continent, the Near East, and Southern Europe. **A)** with projections of key ancient groups from these regions. **B)** Geographic locations of ancient genomes from Africa and Near East included in our analysis. EN: Early Neolithic, LN: Late Neolithic, ChL: Chalcolithic, LSA: Late Stone Age, IA: Iron Age.

**Extended Data Fig. 2:**
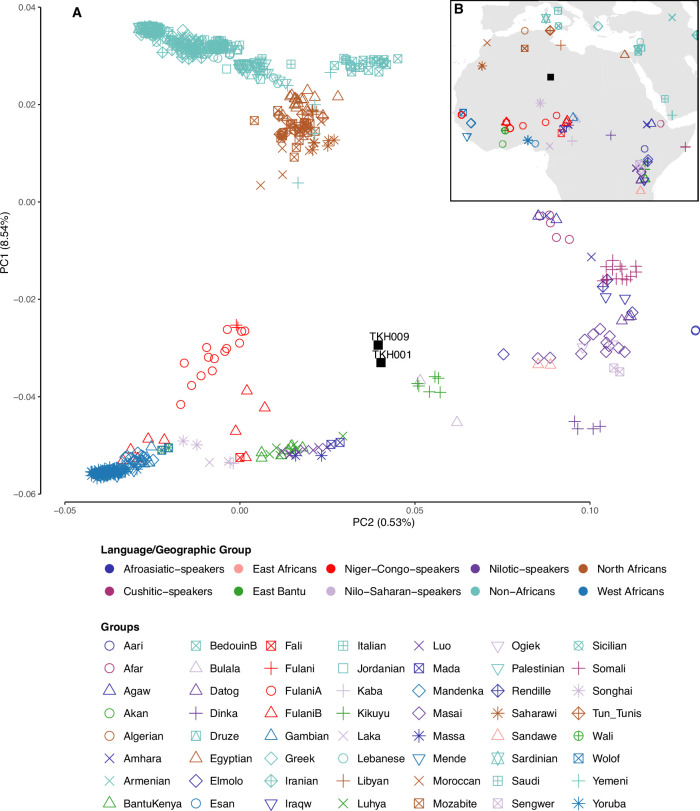
PCA calculated on present-day individuals from West and East Africa, the Sahel, the Near East, and Southern Europe. **A)** with Takarkori individuals projected and highlighted in black squares. **B)** Geographic locations of Takarkori individuals and relevant present-day populations included in our analysis.

To visualize the variation in the genetic ancestry of the Takarkori individuals, we performed a principal component analysis (PCA) using genome-wide data from 795 present-day individuals from the whole African continent, the Near East and Southern Europe, all genotyped on the Human Origins SNP panel^28–38^ (Supplementary Data 1 and 2). We then projected the Takarkori individuals and 117 relevant published ancient genomes^2,8,9,35,36,39–44^ (Supplementary Data 2) onto the first two principal components (PCs). The Takarkori individuals plot broadly intermediate between West African and Near Eastern groups on PC1, albeit closer to West Africans (Extended Data Fig. 1, Supplementary Fig. 2.6 and Supplementary Data 3). To obtain a finer-scale view, we restricted the African populations to West Africa, the Sahel and East Africa, while retaining the Near Eastern and Southern European populations (Supplementary Data 3). This PCA captured the geographical distributions of these populations, whereby PC1 separates African from non-African populations and PC2 differentiates within Africa, particularly separating Sahel/West African from East African populations (Extended Data Fig. 2). Both Takarkori individuals maintained a distinct position, intermediate between Sahel/West and East African populations (Fig. 2a), and formed a tight cluster with overlapping 95% confidence interval (CI) ellipses (Supplementary Fig. 2.4). The PCA projections broadly mirror geography, including the placement of Takarkori. For subsequent population genetic analyses, we merged the low-coverage data from the TKH009 individual with the high-coverage data from the TKH001 individual. We note that many of the signals in this group analysis are probably driven by TKH001 owing to its much higher coverage.

**Fig. 3:**
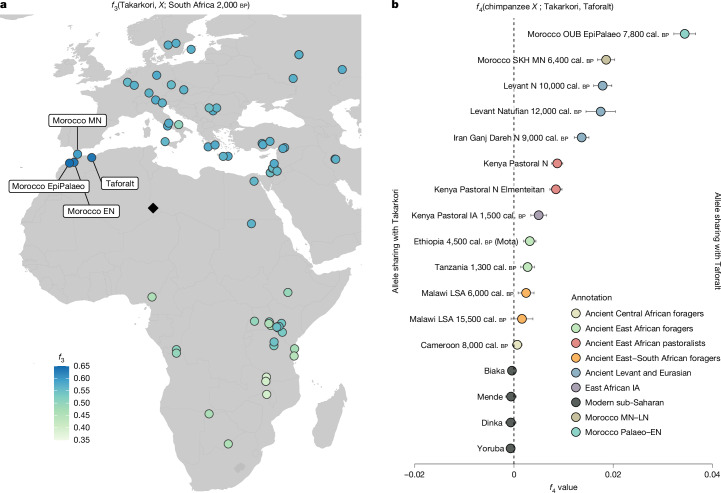
Shared genetic drift and affinity with Takarkori genomes. **a**, Outgroup-*f*_3_ statistics *f*_3_(Takarkori, *X*; South Africa 2,000 cal. bp), where *X* represents ancient groups, mapped at their geographical positions. The colour gradient from blue to green indicates the genetic proximity to Takarkori, with the bluer colours representing closer genetic relationships. The statistics and their associated s.e. values for the top 70 signals are presented in Supplementary Fig. 2.12. **b**, No group shares extra affinity with Takarkori genomes compared with Taforalt, as measured by *f*_4_ statistics of the form *f*_4_(chimpanzee, *X*; Takarkori, Taforalt). The error bars represent 3 s.e. Group colours follow the same scheme as in Fig. 2. A more extensive list is presented in Supplementary Fig. 2.19. LSA, Late Stone Age; N, neolithic.

**Extended Data Fig. 3:**
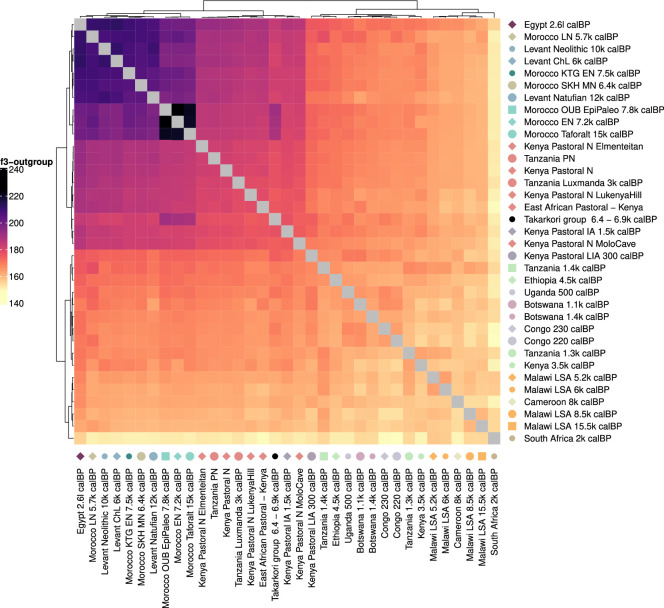
Outgroup-*f*_3_(Chimp; X1, X2) statistics, where X represents relevant ancient populations from Africa and the Near East. Within the shared drift of Takarkori group, the highest genetic drift is exhibited with Taforalt-related groups, OUB and IAM. The colour scheme for populations in the legend follows that of PCA Fig. 1. The error bars represent three standard errors.

To probe for shared genetic drift of Takarkori with other ancient and modern genomes, we computed outgroup *f*_3_ statistics^30^ of the form *f*_3_(Takarkori, *X*; South Africa 2,000 cal. bp), with *X* representing worldwide ancient and present-day test populations and South Africa 2,000 cal. bp as the deepest modern human outgroup lineage^44^. We found that the Takarkori individuals share the most genetic drift with Moroccan Palaeolithic and Early Neolithic individuals. Specifically, we observed the highest level with an Epipalaeolithic individual from Ifri Ouberrid and similarly elevated shared drift with the 15,000-year-old foragers from Taforalt and the Early Neolithic individuals from Ifri n’Amr o’Moussa (Fig. 3a, Extended Data Fig. 3 and Supplementary Fig. 2.12). Both Epipalaeolithic and Early Neolithic groups have been previously shown to maintain high genetic continuity with the much older Taforalt group^8,9^, explaining the similarly elevated shared drift statistics between all three groups and the Takarkori individuals.

**Extended Data Fig. 4:**
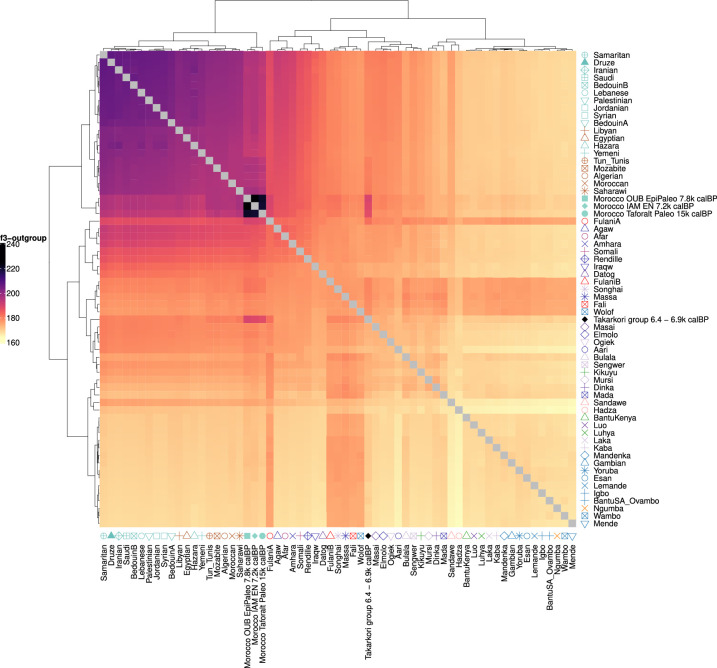
Outgroup-*f*_3_(Chimp; X1, X2) statistics, where X represents relevant modern populations from Africa and the Near East. The Taforalt and later Epipaleolithic and Neolithic groups from Morocco are included for comparison due to their highest genetic drift with Takarkori. The Takarkori group shows the second highest affinity with *FulaniA* after Taforalt-related groups. The colour and shape scheme for populations in the legend follows that of the PCA in Extended Data Fig. 1. The error bars represent three standard errors.

When restricting the comparative groups to present-day African and Near Eastern populations, we found that a group of individuals defined as FulaniA in a previous study^38^, which includes individuals from relatively less admixed Fulani herders from eight Sahelian countries, shows an increased genetic affinity to Takarkori, although less so to Taforalt (Extended Data Fig. 4 and Supplementary Fig. 2.16). This finding aligns with ref. ^38^, which observed a non-sub-Saharan ancestry component in FulaniA similar to that found in the Taforalt and Late Neolithic Moroccans. To further probe for Takarkori-like ancestry in FulaniA, we conducted an *f*_4_ analysis *f*_4_(chimpanzee, *X*; Masai/Datog/Iraqw, Takarkori), using Masai/Datog/Iraqw as baseline references owing to their similar amount of out-of-Africa (OoA) ancestry to Takarkori (Supplementary Fig. 2.20.1–2.20.3). The results indicated that the FulaniA have an increased affinity to Takarkori-like ancestry, as do other Sahelian and West African groups. These findings are consistent with the archaeological evidence of the southward expansion of Pastoral Neolithic groups from Central Sahara. Rock art, ceramic production and funerary practices provide detailed indications of the spread of these herders at the end of the Middle Holocene, probably driven by the progressive aridification of the Central Saharan regions^45,46^.

**Extended Data Fig. 5:**
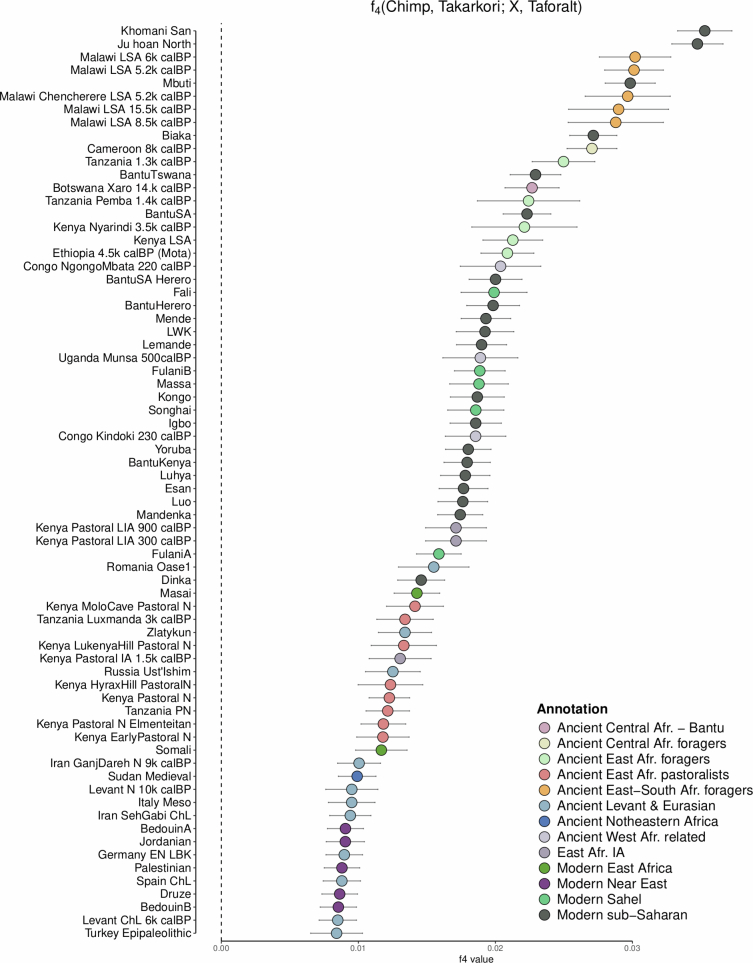
Results of f_4_(Chimp, Takarkori, X, Taforalt) analysis with X representing various ancient and modern populations from Africa and the Near East. All positive f4 values underscore shared genetic drift between Takarkori and Taforalt that surpasses other potential affinities tested here. The error bars represent three standard errors.

Given the high amount of shared genetic drift between the Takarkori and the ancestry first appearing in the 15,000-year-old Taforalt individuals in the outgroup *f*_3_ statistics, we subsequently investigated whether the Takarkori genomes share more alleles with other human groups compared to the Taforalt genome. For this analysis, we computed the statistics *f*_4_(chimpanzee, *X*; Takarkori, Taforalt), where *X* represents ancient and present-day groups from Africa and Eurasia. We obtained significantly positive values for Eurasian and Eurasian-admixed African groups, suggesting that the Taforalt group shares more alleles with these groups than Takarkori does. Conversely, none of the ancient or modern groups tested showed a significantly negative signal, indicating no detected closer affinity with Takarkori than Taforalt. Notably, both ancient and modern sub-Saharan groups, who are mostly unadmixed with Eurasian groups, yielded no significant value (|*Z*| < 3), suggesting that these groups are equally distant from both the Takarkori and Taforalt groups (Fig. 3b and Supplementary Data 4). Moreover, when running statistics *f*_4_(Chimp, Takarkori; *X*, Taforalt), we found significantly positive values for every population *X*, indicating that Takarkori shares more alleles with Taforalt than any other group (Extended Data Fig. 5 and Supplementary Data 4).

**Extended Data Fig. 6:**
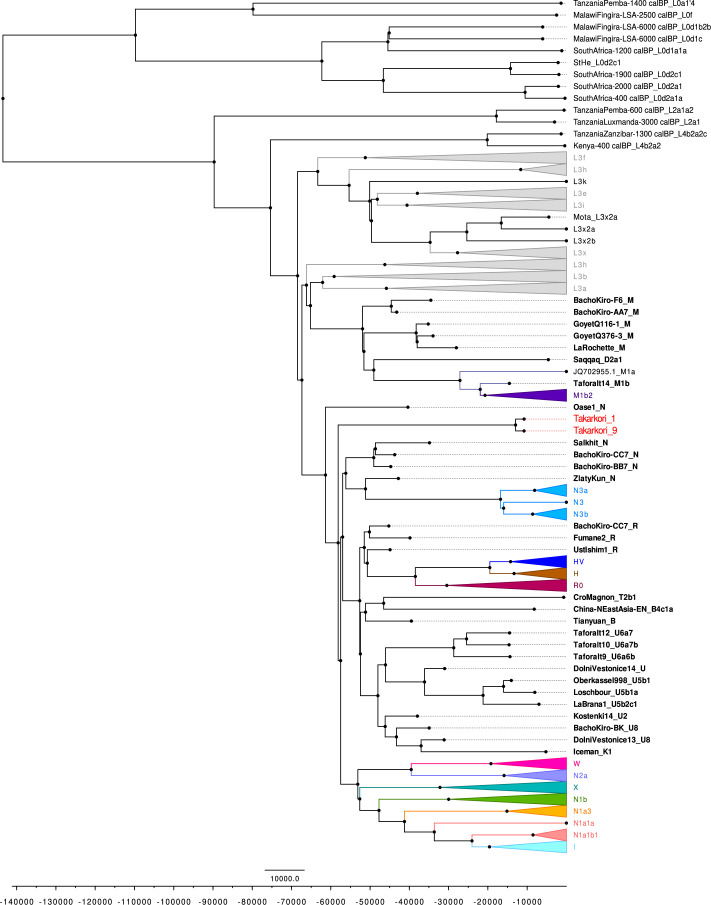
Bayesian Mitochondrial phylogeny of Takarkori samples and comparative genomes. The phylogenetic tree is constructed for the Takarkori samples TKH001 and TKH009, combined with 209 published complete genomes from ancient and modern samples. The major mitochondrial lineages and sub-lineages for the N macrohaplogroup are differentiated by distinct colours.

At the mtDNA level, both Takarkori individuals belong to a basal branch of haplogroup N, representing one of the deepest mtDNA lineages outside sub-Saharan Africa and predating present-day N-derived mtDNAs^13^. Using BEAST analysis for mtDNA dating, which included additional sequences from Upper Palaeolithic individuals and the dataset described previously^13^, we corroborate the previous findings of ref. ^13^ that the Takarkori individuals carried a basal N haplogroup lineage^13^, and refined the molecular split date estimate to 61,343 years old (95% highest posterior density (HPD) = 54,408–69,046) (Extended Data Fig. 6). Notably, the mtDNA lineage of the Oase 1 individual falls more basal to haplogroup N, suggesting an earlier split from the OoA lineage before the divergence of the Takarkori lineage. However, owing to incomplete lineage sorting and mtDNA representing a single lineage, the exact timing of the underlying population splits remains uncertain.

**Extended Data Fig. 7:**
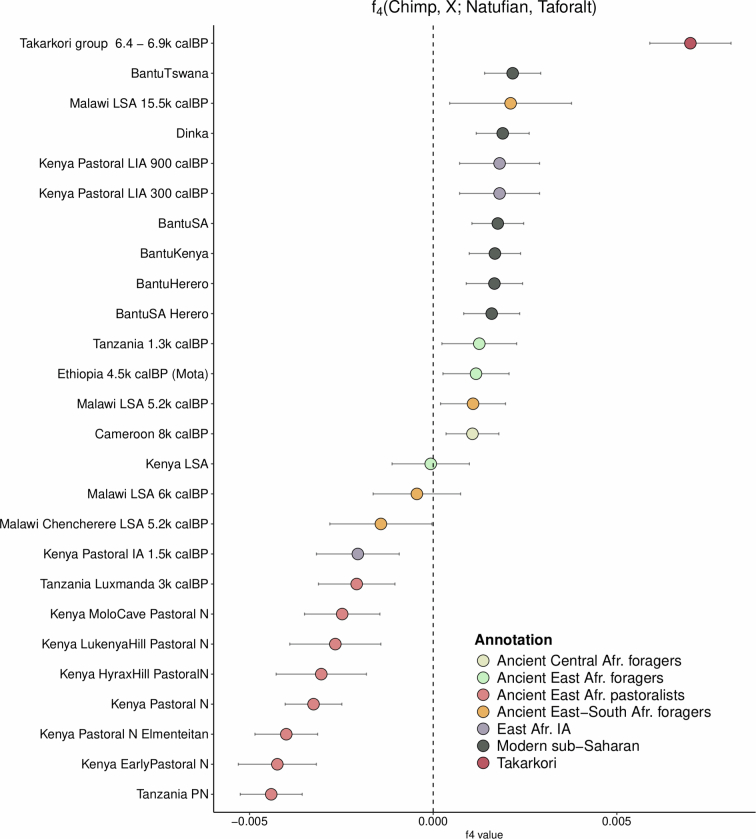
Results of *f*_4_(Chimp, X, Natufian, Taforalt) analysis with X representing various ancient African groups. Takarkori exhibits a notably higher affinity to Taforalt, with an f4 value approximately 3.5 times higher than the next closest African group. The error bars represent three standard errors.

**Extended Data Fig. 8:**
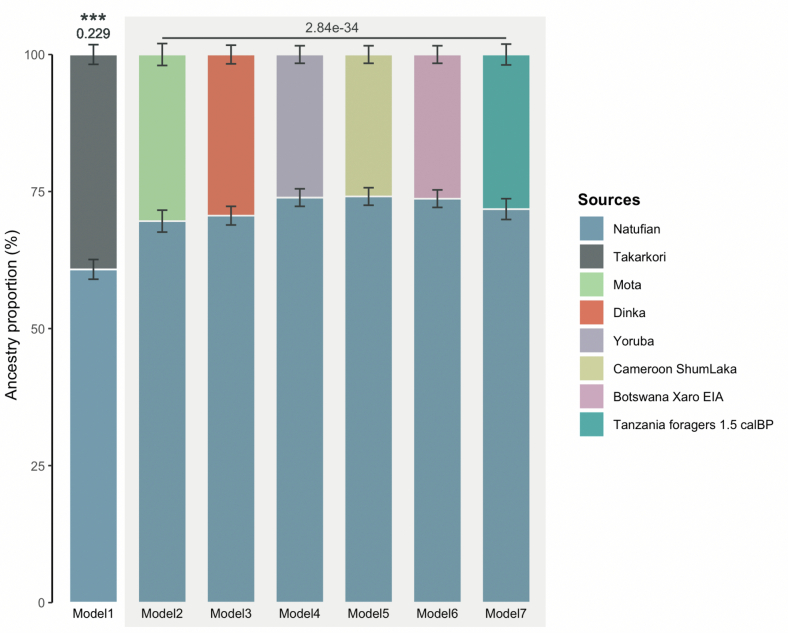
qpAdm analysis of the Taforalt group. Results of modelling the Taforalt group as a two-way admixture between Natufian and various African populations. Takarkori yielded the best model fit with a P-value > 0.05, indicating the sufficiency of the two-way admixture model for Taforalt. In contrast, the P-values for the other models were all <2.84 × 10^−34^. The error bars represent the standard error of the ancestry proportion estimates, calculated using 5 cM block jackknifing.

Previous work modelled the Taforalt group’s ancestry as a two-way admixture of approximately 63.5% Natufian (ancient Levantine foragers) and 36.5% sub-Saharan African ancestries^2^. However, this model using the software qpAdm^2^ could not pinpoint the origin of Taforalt’s African ancestry, resulting in unknown ghost ancestry only broadly linked to South, East and Central African groups^2^. Here we included Takarkori as a possible source of the African ancestry in Taforalt in comparison to several potential sources (namely Yoruba, Dinka, Mota, Cameroon Shum Laka, Botswana Xaro Early Iron Age (EIA) and Tanzania Zanzibar 1,300 cal. bp) through rotation-based qpAdm. We found that Saharan Takarkori provides a much better fit as an African proxy for Taforalt than the sub-Saharan groups, attaining a *P* value of >0.05, indicative of a much better model fit compared to the other sources (*P* < 2.84 × 10^−34^) (Extended Data Figs. 7, 8 and Supplementary Tables 2.6, 2.7). With this revised model, we estimated that the Taforalt ancestry retains a comparable 60.8% (±1.8%) contribution from Natufians, with the remaining 39.2% (±1.8%) derived from Takarkori.

**Extended Data Fig. 9:**
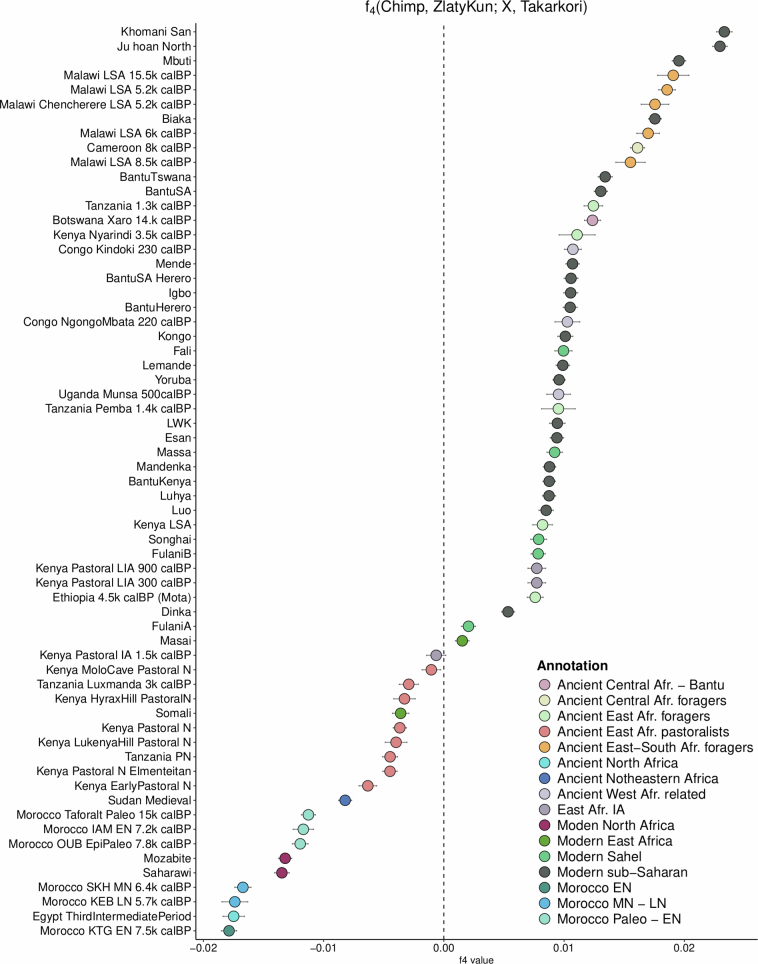
Assessment of Takarkori’s affinity with OoA ancestry, represented by Zlatý kůň, versus various African groups. The results suggest a stronger connection between Takarkori and the OoA ancestry compared to sub-Saharan Africans. The error bars represent three standard errors.

We next explored the direct genetic affinity between Takarkori and the OoA ancestry found in all non-African modern humans, in contrast to African groups. For this, we computed *f*_4_ statistics of the form *f*_4_(chimpanzee, Zlatý kůň; African, Takarkori). Zlatý kůň, a 45,000-year-old Upper Palaeolithic individual from Czechia, was used as a proxy for OoA ancestry as it is probably the oldest modern human sequenced to date and represents the deepest known human lineage after the OoA lineage splits from the African lineages^47^. Our results showed positive values for Takarkori, indicating that it is genetically closer to Zlatý kůň than to sub-Saharan Africans, including Mota, a 4,500-year-old genome from East Africa. Nevertheless, various African populations with substantial OoA admixture were still genetically closer to Zlatý kůň than to Takarkori (Extended Data Fig. 9 and Supplementary Data 5).

**Fig. 4:**
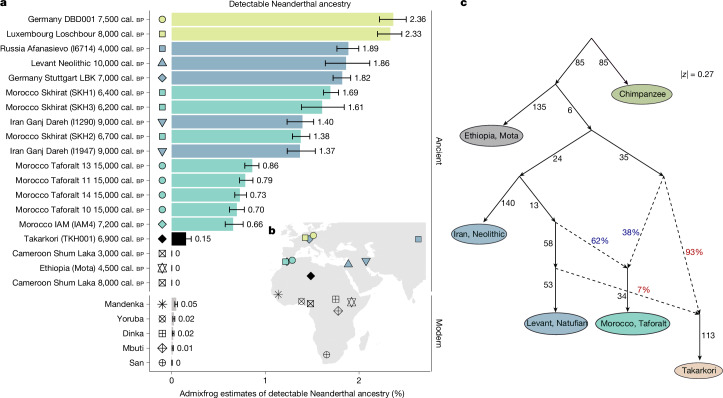
Neanderthal ancestry and admixture graph. **a**, Detectable Neanderthal ancestry in segments longer than 0.05 cM in ancient individuals from Africa and Eurasia, along with present-day sub-Saharan African groups. The error bars represent the minimum and maximum estimates from all iterations. **b**, The geographical locations of groups included in the analysis. **c**, Admixture graph modelling of Takarkori’s ancestral relationship with relevant populations.

**Extended Data Fig. 10:**
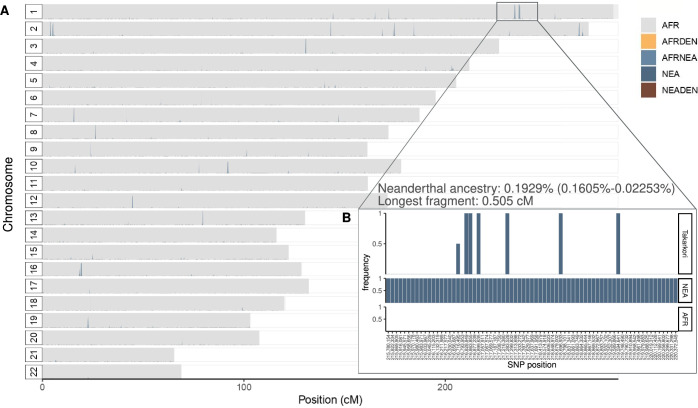
Neanderthal ancestry. **A)** Detected archaic ancestry fragments in Takarkori and Taforalt inferred by admixfrog. Dark and light blue regions are homozygous and heterozygous Neanderthal fragments, respectively. Grey fragments indicate African ancestry and bar height is proportional to the posterior probability of ancestry. **B)** The longest archaic fragment in Takarkori is on chromosome 1 compared to the reference panel. The y-axis refers to the frequency of the alternative allele in the reference population (Africans or Neanderthals in this case) at the given position, and the x-axis stands for the SNP positions.

These results raise the question of whether Takarkori’s ancestors are closely related to OoA groups but remained in Africa, or whether they received later gene flow from OoA groups. If Takarkori experienced such later gene flow, it would carry Neanderthal admixture that is found in all OoA groups. To explore this signal, we used the data generated using the Archaic Admixture SNP panel for Takarkori and the software admixfrog^48^ that detects Neanderthal segments and included other ancient African and Eurasian groups as well as modern sub-Saharan African populations for comparison (Supplementary Note 2). We detected a total of 12 Neanderthal fragments that surpass 0.05 cM in length (approximately 50 kb), with the longest fragment located on chromosome 1 (Extended Data Fig. 10 and Supplementary Fig. 2.32), translating to a low level of Neanderthal ancestry in the Takarkori genome of approximately 0.15% (Fig. 4a). This percentage is less than a quarter of the Neanderthal ancestry in segments longer than 0.05 cM found in Taforalt and Neolithic Morocco individuals (0.6–0.9%), and about tenfold lower than in most OoA groups (1.4–2.36%), yet significantly higher than that in other ancient and contemporary sub-Saharan African genomes, where Neanderthal ancestry was completely absent. This pattern suggests that the Takarkori individuals have received a small amount of ancestry from OoA groups. However, the estimation of admixture dating of this OoA ancestry with DATES^49^ based on linkage disequilibrium (Supplementary Figs. 2.26.1–2.26.3) indicated very ancient admixture events with substantial uncertainty.

Finally, we used the find_graphs() function from the ADMIXTOOLS 2^50^ package to model Takarkori’s ancestral relationship with other populations. We used a function for automated graph exploration with a model that includes Mota, Iran Neolithic, Natufian, Taforalt, Takarkori and the outgroup chimpanzee. The model fits with small *f*-statistic residuals (max |*f*_4,expected_ − *f*_4,observed_| = 0.27 s.e.). The fitted graph suggests that Takarkori traces most of its ancestry (93%) to a hitherto unknown North African population, in agreement with the isolation signature obtained from the *f*_4_ statistics mentioned above (Fig. 3b). This unknown North African population is closely related to OoA populations and branches off the lineage leading to OoA later than the ancient individual from Mota Cave, Ethiopia, who represents the sub-Saharan African lineage most closely related to OoA groups identified so far. In our model, the remainder of the Takarkori individuals’ ancestry (7%) is derived from a deeply ancient Levantine source. The Levantine gene flow also accounts for the Neanderthal ancestry found in Takarkori as the Levantine Neolithic genomes carry around 1.86% Neanderthal ancestry, aligning with our estimates using admixfrog. The graph also models Taforalt as a mixture of a 40% contribution from a Takarkori-related branch and 60% from a Natufian-related branch, consistent with our qpAdm results (Fig. 4c and Supplementary Data 6).

## Discussion

Our study introduces ancient genome-wide data from humans that inhabited the Green Sahara, providing unique insights into the genomic ancestry of populations in this region. The individuals from the Takarkori rock shelter predominantly carry an ancestral African lineage, representing an ancestry profile that has not been previously described. They share the most genetic drift with Epipalaeolithic and Early Neolithic individuals from Ifri Ouberrid and Ifri n’Amr o’Moussa, as well as the 15,000-year-old foragers from the Taforalt Cave in Morocco, suggesting a long-standing and stable population in North Africa before the AHP (14,500–5,000 bp). Plausibly, this ancestry was present in large parts of Northern Africa after the OoA event, and the Takarkori individuals inherited it from the group that inhabited the area during the final period of the Late Acacus (10,200–8,000 cal. bp). In Southwestern Libya, this period preceded the arrival of domesticates and is characterized by cultural advancements within those hunter–gatherer groups^10^. This included an increase in sedentism^51^, and the use of sophisticated material cultures such as pottery, basketry, and bone and wooden tools^52–54^.

We found that individuals from Takarkori exhibit only a marginal amount of genetic admixture from Levantine groups, suggesting that the emergence of pastoralism in the Sahara was primarily driven by the dissemination of cultural practices rather than through large-scale human migration, as suggested on archaeological basis^10^. Material culture at the onset of the earliest pastoral period shows both continuity and change, reflecting possibly complex assimilation dynamics during socioeconomic transitions^10,19^. These patterns may further suggest gradual cultural transformations rather than abrupt population replacement. This interpretation is further supported by the comparatively lower levels of Neanderthal genetic admixture found in the Takarkori individuals. Our admixture dating analysis points to events far back in time, suggesting a more heterogeneous spread of pastoralism and food production in the Sahara compared to Morocco and East Africa, where there was a noticeable increase in recent Levantine genetic admixture^9,35,39,43^. In addition to these findings, a run of homozygosity (ROH) analysis for TKH001 revealed no ROH segments larger than 12 cM, indicating no close-kin inbreeding (Supplementary Fig. 2.28). Several shorter ROH segments over 4 cM suggest an effective population size (*N*_e_) of around 1,000 individuals, reflecting a moderately sized population.

Our research offers insights into the ancestry of the previously published Taforalt hunter-gatherers. While a previous study^2^ could not precisely ascribe the ‘sub-Saharan’ component in the Taforalt genome, we now identify this ancestry as a deep North African lineage, with higher proportions found in the Saharan Takarkori individuals. This refines the earlier model, which proposed a dual admixture of Natufian and broadly sub-Saharan African ancestries. Our updated model suggests that the Taforalt ancestry is composed of a 60% contribution from a Natufian-like Levantine population, with the remaining 40% derived from a Takarkori-like ancestral North African population (Extended Data Fig. 8). Notably, both the late Pleistocene Taforalt and the mid-Holocene Takarkori individuals demonstrate equally distant relationships with sub-Saharan African lineages. This pattern suggests that no substantial genetic exchanges across the Green Sahara occurred during the AHP or other humid periods preceding the Later Pleistocene. The Sahara, spanning around 9 million km^2^ and housing diverse biomes, such as grasslands, wetlands, woodlands, lakes, mountains and savannas^55,56^, probably saw fragmented habitats impacting human gene flow. These ecological barriers, combined with social and cultural barriers, spatial structuring of populations, and the selective adoption of specific practices, may have facilitated the widespread dissemination of similar archaeological features^57,58^, while limiting extensive genetic admixture. This genetic discontinuity is consistent with modern data, which show substantial genetic differentiation across the Sahara beyond just a geographical gap^59^. Our findings suggest that sporadic Green Sahara events, particularly before pastoralism, were insufficient to allow for considerable genetic exchange, mirroring the Sahara’s persistent role in limiting human genetic flow, as reflected in both ancient and modern population structures.

Our findings represent an important initial step, and future genetic studies could reveal more refined insights into human migration and gene flow across the Sahara. As sequencing costs continue to fall, whole-genome sequencing could enable more unbiased estimates regarding OoA events and other key aspects of human evolution.

## Methods

### Inclusion and ethics

The present-day communities of the Tadrart Acacus region, the Kel Tadrart pastoral group, have been actively engaged in both the excavation activities at the site (field assistance, sieving and so on) and in the decision-making processes and actions related to its conservation efforts. Other local community members contributed to the logistical management of the mission’s tented camp, performing various specialized labour roles.

Although these communities maintain centuries-long connections to the territory, they do not express a specific cultural or historical affinity with the prehistoric burials uncovered there. This sentiment extends to the broader archaeological contexts and local rock art, which are viewed as expressions of ancient, pre-Islamic times, for which no direct cultural link is recognized or claimed.

Despite the lack of a perceived direct connection, ethical considerations and the implications of analysing and handling human remains have been thoroughly discussed with the Department of Antiquities (DoA) in Libya, with substantial input from M.F.M.A.-F. and M.T., both affiliated with the same institution. After the excavation concluded, and in subsequent years, the results were shared in meetings held at the local governorate office in Ghat. Unfortunately, the onset of the Libyan Revolution in 2011, shortly after the excavation activities ended, disrupted further contact. However, over the past year, coinciding with the preparation of this manuscript, online meetings with these co-authors and other DoA representatives have resumed, fostering a renewed dialogue on these issues. These inclusive approaches have ensured that both scientific research and conservation practices are aligned with local values and ethical standards, promoting a collaborative framework for heritage management and scientific collaboration.

### Archaeology and bioarchaeology

Takarkori rock shelter counted 15 burials, varying in phases and preservation states, predominantly from the Early and Middle Pastoral periods, spanning the eighth and the seventh millennium  cal. bp^16,22^. The burials, found immediately adjacent to the rock wall and with scarce grave goods, appear to only consist of women and children^13,22,61^ (Supplementary Note 1). Osteological information is reported and discussed elsewhere^22^, although, in brief, age estimation for subadult individuals was based on methodologies for skeletal development^62,63^ and dental maturation and eruption^64^ developed previously. For adult individuals, sex determination was based on the observation of morphological traits of the skull and the pelvis, as synthesized previously^65^, whereas estimation of age at death was based on the modifications of the pubic symphysis as proposed previously^66^, cranial sutures closure^67^ as well as patterns of occlusal dental wear^68^, which took into account the possible role of the sandy environment. Detailed layouts of the excavation areas, along with the stratigraphic and chronological insights, are depicted in Supplementary Fig 1.4. Standard stratigraphical techniques were used in the excavation of skeletal remains, with strict adherence to precautions for the sampling of biological materials. Sediments associated with the remains underwent sieving using a 2 mm mesh, and samples for laboratory-based studies were collected. The excavation and handling of human remains have been extensively discussed with the Department of Antiquities (DOA) in Tripoli, Libya (formerly the Socialist People’s Libyan Arab Jamahiriya), particularly concerning ethical issues. Approval for the archaeological excavation was granted on 28 January 2004, under the reference number  (translated as H98-10000 TATM 49463). Currently, the human remains are curated at the Museum of Anthropology of the University of Rome, La Sapienza.

### Ancient DNA processing

Tooth root from individual TKH001 and fibula fragments from individual TKH009, both previously labelled as TK H1 and TK H9, respectively^13^, were sourced for this study. The sampling and DNA extraction procedures were carried out in a specialized ancient DNA facility at the University of Florence’s Molecular Anthropology department, as outlined previously^13^. DNA was extracted according to the protocol described previously^69^. From the extracted DNA, a double-stranded DNA library was prepared in the same Florence facility, without undergoing uracil-DNA-glycosylase (UDG) treatment. An additional single-stranded DNA library, better at capturing short fragments, was prepared at the Max Planck Institute of Geoanthropology (formerly Max Planck Institute for the Science of Human History, MPI-SHH) and/or the Max Planck Institute for Evolutionary Anthropology (MPI-EVA). The prepared DNA libraries underwent shotgun sequencing to a depth of 3–5 million reads, using 75 bp single-end and/or 50 bp paired-end configurations on the Illumina HiSeq 4000 system at MPI-EVA for initial quality assessment (Supplementary Table 2.1). Both TKH001 and TKH009 libraries underwent in-solution capture targeting over a million SNPs using the Twist Ancient DNA^25^ and 1240k^26^ panels, with an additional specialized ‘archaic ancestry’ panel applied to the single-stranded library from TKH001. After enrichment, all of the libraries were sequenced to 20 million reads on the Illumina HiSeq 4000 system at MPI-EVA. Read adapters were removed using AdapterRemoval^70^ v.2.3.0 as part of the EAGER (v.1.92.56)^71^, and the genome-wide captures were aligned to the human reference genome (hg19) using a mapping quality filter of 25 with BWA aligner^72^ v.7.12. Duplicate reads were eliminated using DeDup (v.0.12.2), which can be found at GitHub (https://github.com/apeltzer/DeDup). The contamination of the single-stranded library from TKH001 was evaluated with AuthentiCT^73^ v.1.0 and hapCon_ROH (https://haproh.readthedocs.io/en/latest/hapROH_with_contamination.html), and Schmutzi^74^ was used for the mtDNA-captured double-stranded library from TKH009 (Supplementary Table 2.3). The damage pattern was assessed with DamageProfiler^75^ v.1.1.

Double-stranded 1240k-captured sequences from TKH009 and single-stranded Twist-captured sequences from TKH001 were genotyped using Samtools v.1.3 and pileupCaller from SequenceTools v.1.4.0.2 (https://github.com/stschiff/sequenceTools). TKH001 has a total of 881,765 SNPs on the 1240k panel, and TKH009 libraries showed lower coverage of 22,484 SNPs. We merged the genotype data from TKH009 and TKH001. We then ran all the analyses throughout the manuscript (including PCA, *f*_3_ and *f*_4_ statistics, qpAdm, admixture graph, DATES and ADMIXTURE), except for admixfrog and hapROH, which, as individual analyses, use only the higher-coverage data from TKH001. To prevent potential artifacts from affecting the results, additional versions of the genotyped data for the TKH001 sample were generated. These included data from only the single-stranded library, data restricted to transversions only and data filtered using PMDtools (v.0.6)^76^ (Supplementary Table 2.4).

### Population genetic analyses

Using smartpca^77^ v.16000 from EIGENSOFT package v.8.0 with lsqmode enabled, PCAs were conducted on present-day individuals from Africa, Middle East and Southern Europe, genotyped on the Human Origins SNP panel^28–38^, alongside pertinent ancient groups from these regions^2,8,9,35,36,39–44^. All *f*_3_ and *f*_4_ statistics were calculated using the ADMIXTOOLS^30^ package v.5.1, using qp3pop v.435 for *f*_3_ statistics and qpDstat v.755 for *f*_4_ statistics. For the *f*_3_ outgroup test, the topology *f*_3_(outgroup; *X*, Takarkori) was used. When the outgroup was set as South Africa 2000 cal. bp, the ‘inbreed’ option was enabled for qp3pop. On the other hand, when the outgroup was chimpanzee, the ‘outgroupmode’ option was activated in qp3pop. This particular configuration was used to set a constant denominator of 0.01, as the inbuilt heterozygosity normalization in qp3pop does not function correctly when the outgroup is haploid, as in the case of the Chimpanzee. Furthermore, we used the Affymetrix Human Origins 1 Array, which comprises 13 unique SNP sets, to investigate potential sub-Saharan ancestry in the Takarkori and Taforalt populations. We conducted separate *f*_4_ statistics for Mbuti and Yoruba ascertained SNPs (Supplementary Figs. 2.21 and 2.22).

The Taforalt group was modelled as a two-way admixture between the Natufian population (left source) and variable African groups (right source) using qpAdm^78^ v.810 from ADMIXTOOLS package v.5.1. For every run, a consistent outgroup set was arranged, comprising Onge, Han, Papuan, Ust’-Ishim, Kostenki14, MA-1 and Iran_N. Every African group (Yoruba, Dinka, Mota, Cameroon Shum Laka, Botswana Xaro EIA, and Tanzania Zanzibar 1,300  cal. bp) was examined as a potential right source population in individual runs, while being omitted from the outgroup set (Supplementary Table 2.6). This methodology enabled the exploration of various admixture scenarios for the Taforalt group.

Neanderthal ancestry was inferred using admixfrog^48^ v.0.7.1 on single-stranded Takarkori and Taforalt libraries captured with the Archaic Admixture SNP panel^27^. The analysis included high-quality ancient shotgun sequencing data and present-day sub-Saharan genomes from the Allen Ancient Genome Diversity Project/John Templeton Ancient DNA Atlas (https://reich.hms.harvard.edu/ancient-genome-diversity-project) and the Human Genome Diversity Project^32^. Genomes were subsetted to positions in the Archaic Admixture SNP panel, with filters for mapping quality and base pair length. Libraries with insufficient SNP coverage or contamination were excluded (Supplementary Table 2.8). High-coverage genomes were subsetted to the positions covered by the Takarkori genome for direct comparison. The reference panel included three high-coverage Neanderthals^79–81^, one high-coverage Denisovan^33^ and the chimpanzee genome (panTro4) as the ancestral allele, along with Sub-Saharan African 1000 Genomes sequences^82^ for the African state.

The BEAST analysis of mitochondrial genomes involved 216 mtDNA sequences, aligned using MAFFT^83^ v.7.508 and adjusted by removing specific poly-C regions. The Hohlenstein–Stadel Neanderthal^84^ was the outgroup, and mtDNA sequences not in GenBank were processed using a specialized pipeline (mitoBench-ancientMT)^85^. The sequence consensus was determined using snpAD^86^ v.0.3.9, with haplogroups assigned by HaploGrep2^87^ v.2.1.19. BEAST^88^ v.2.6.7 used these alignments, incorporating radiocarbon dates from Supplementary Table 2.9 as priors. Model suitability was assessed with bModelTest^89^ v.1.2.1, determining a combination of TIM(21) + *I* + *G* model, strict clock rate and ‘coalescent Bayesian skyline’ tree prior as optimal. Eight independent analyses were conducted, combining results using LogCombiner and TreeAnnotator.

The ancestral relationship of the Takarkori population with others was investigated using the ‘find_graphs()’ function from ADMIXTOOLS 2, which finds admixture graphs aligning with observed *f* statistics. Chimpanzee was set as an outgroup, and we included representatives from key relevant genetic clusters: ‘Morocco_Iberomaurusian’, ‘Ethiopia_4500BP.SG’, ‘Israel_Natufian_published’ and ‘Iran_Ganj_Dareh_Neolithic’. Given the potential variability of ‘find_graphs()’ outputs, 20 iterations were performed, using unique random graphs for each run. Graphs with a *Z* score of <|3| were selected (Supplementary Figs. 2.23–2.25). Moreover, Takarkori and Taforalt groups were treated as admixed populations, guided by previous knowledge from admixfrog results.

The DATES method^49^ v.753 was applied to the Takarkori group to detect Levantine admixture. A bin size of 0.001 and a fit range of 0.0045 to 1 in Morgan units were used. Admixture dates were estimated based on the decay of linkage disequilibrium between SNP pairs, with Levantine admixture modelled using sub-Saharan populations (Yoruba, Mbuti, Dinka, ancient groups from Ethiopia and Tanzania) and Eurasian populations (Natufian, PPNB, Ganj Dareh) (Supplementary Figs. 2.26.1–2.26.3).

The effective population size (*N*_e_) for TKH001 was estimated using hapROH^90^ v.3.0. ROH segments longer than 4 cM were inferred using hapROH’s recommended default settings, using the 1000 Genomes reference panel for comparison (Supplementary Fig. 2.27). This method analyses runs of homozygosity to estimate the population size and genetic background of the individual (Supplementary Fig. 2.28).

ADMIXTURE^91^ v.1.3.0 was used for unsupervised genetic clustering of global populations. Modern and ancient groups were subsetted from the HO-based dataset, converted to PLINK format, and transposed to pseudohaploid format to reduce artificial drift. Linkage disequilibrium pruning was performed using PLINK^92^ v.1.9 with a window size of 200 SNPs, a step size of 25 SNPs and an *r*^2^ threshold of 0.4. Five replicates for each *k* (2–9) were run, selecting the replicate with the highest log-likelihood.

Data visualization was performed in RStudio v.2022.12.0 + 353. The following R packages were used cowplot (v.1.1.2), ggplot (v.3.4.2), ggh4x (v.0.2.3), ggnewscale (v.0.4.8), janno (v.1.0.0), magrittr (v.2.0.3), maps (v.3.4.1), patchwork (v.1.1.2), purrr (v.1.0.1), RColorBrewer (v.1.1.3), readxl (v.1.4.1), tidyr (v.1.3.0) and tidyverse (v.1.3.2).

### Reporting summary

Further information on research design is available in the Nature Portfolio Reporting Summary linked to this article.

## Online content

Any methods, additional references, Nature Portfolio reporting summaries, source data, extended data, supplementary information, acknowledgements, peer review information; details of author contributions and competing interests; and statements of data and code availability are available at 10.1038/s41586-025-08793-7.

## Supplementary information

Supplementary Information Supplementary Notes 1–3, including Supplementary Figs., Tables and References – see Table of Contents for details.

Reporting Summary

Supplementary Data Supplementary Data 1–6.

## Data Availability

All newly reported ancient nuclear DNA data are archived in the European Nucleotide Archive (PRJEB84057).
