# Supplementary material for: Ancient DNA from the Green Sahara reveals ancestral North African lineage
Source: Nature. 2025 Apr 2;641(8061):144–50. doi: 10.1038/s41586-025-08793-7 (PMC12043513; doi:10.1038/s41586-025-08793-7)
Supplement: Supplementary file 1 — Supplementary Notes 1–3, including Supplementary Figs., Tables and References – see Table of Contents for details. [file 41586_2025_8793_MOESM1_ESM.docx]

**Supplementary Material**

Ancient DNA from the Green Sahara reveal ancestral North African lineage

# Table of Contents

[**Table of Contents 2**](#_1ungoeywxmv5)

[**Supplementary Note 1: Environmental and cultural context of the Tadrart Acacus Region 4**](#_wsn09zmfv6um)

[The African Humid Period 4](#_mx8ccum15qfb)

[Role of Earth’s orbit and tilt in deriving the AHP 4](#_xabam5vm1k7o)

[Vegetation expansion during AHP 4](#_weft0rj99ihv)

[The termination of AHP 5](#_t5rcqi287jmp)

[The role of climate on Holocene societies in the Tadrart Acacus 5](#_dkd8zrxzg9nj)

[Takarkori rockshelter 6](#_dqx0e6p3kgmt)

[Burial and sampled individuals 12](#_5pku69hspi81)

[**Supplementary Note 2: Ancient DNA processing and population genetic analyses 14**](#_9i3itqi16nxt)

[Sampling and DNA extraction 14](#_gqn7t2ctpg7v)

[Double-stranded library preparation 14](#_l7oxclus21aj)

[Single-stranded library preparation 15](#_dmuujbnqlvoj)

[Shotgun sequencing and target enrichment of nuclear DNA 15](#_yyktt1ylvdei)

[**Population genetic analyses 19**](#_8qd1rberchrf)

[Analyzing enriched libraries 19](#_phxju4fd4qzs)

[Spurious alignment estimate 19](#_t6uyx6936sq2)

[Ancient DNA contamination 19](#_n21o4nobw18l)

[Genetic sex determination 20](#_c6fkqcwboo0j)

[Genotyping 21](#_7oz4h7qyy5b3)

[Data validation 23](#_hbha63tpqdo0)

[95% Confidence interval ellipse for PCA 23](#_5lvn7y1rfnia)

[PCA without projection 25](#_pi2y6mn1s4of)

[Comparative dataset 27](#_559x5cp0au4s)

[Comprehensive SNPs dataset 27](#_maluf8ge3l09)

[Reduced SNP dataset 27](#_jwa45xmhyyy1)

[The 184K SNP dataset 28](#_iwaj9an45r37)

[Principal component analysis 29](#_21i1annunk9k)

[F-statistics 37](#_rw6osg1cvxyq)

[Outgroup-f3 37](#_3f7ipkcrj2hq)

[1. Affinity with ancient populations 37](#_spp8os8lq9iv)

[2. Affinity with present-day populations 43](#_m6tvhpmue457)

[f4-statistics 47](#_ne8gm4eb4hsv)

[f4-statistics based on SNPs ascertained in African populations 52](#_l6ki66w33lb6)

[qpAdm modeling for Taforalt 55](#_mrmh5f1vehqi)

[Admixture graph analysis 57](#_r160md4a9vq)

[DATES 61](#_bsta4xkaobqk)

[Estimation of effective population size Ne with hapROH 65](#_abd4hzjuosbk)

[ADMIXTURE 68](#_hn2mhzgtqng)

[Admixfrog 72](#_y89j1sb8yaur)

[Estimating Neanderthal ancestry percentage 76](#_8jnzelxb84g4)

[BEAST analysis of mitochondrial genomes 80](#_pkzq3ynub3i0)

[Technical details of BEAST analysis 85](#_c1j87ehxj5ay)

[**References 86**](#_3b0nbm16ki0k)

[**Supplementary Note 3: Non-peer reviewed abstract in Arabic**](#_lfs2ct1sdafi) **91**

# Supplementary Note 1: Environmental and cultural context of the Tadrart Acacus Region

## The African Humid Period

The African Humid Period (AHP) was a time of significant climatic shift in Africa, occurring from roughly 14,800 to 5,500 years ago. During this time increased rainfall led to the expansion of vegetation across what is now the Sahara Desert. This period, also known as the "**Green** **Sahara**" was characterized by a monsoon climate with rainfall patterns significantly more pronounced than in the present day[^1^](https://paperpile.com/c/AGF0JC/tpNO3). Geological evidence, such as lake sediments and pollen records, have provided valuable insights into these dramatic environmental changes[^2^](https://paperpile.com/c/AGF0JC/x2S0j).

### Role of Earth’s orbit and tilt in deriving the AHP

During the AHP, changes in the Earth's axial tilt and orbit amplified summer insolation or incoming solar radiation in the Northern Hemisphere. This increased solar radiation intensified monsoon activity that resulted in more summer rainfall in North Africa[^1,3^](https://paperpile.com/c/AGF0JC/kyWiy+tpNO3). Additionally, the variation in seasonal sunlight led to a stronger Mediterranean winter rainfall system that extended further south, causing increased winter rainfall in the Mediterranean and areas further south than before[^4^](https://paperpile.com/c/AGF0JC/QbemM).

### Vegetation expansion during AHP

During the AHP, the increased rainfall contributed to the proliferation of grasslands and shrubs across North Africa, replacing the arid desert environment[^5,6^](https://paperpile.com/c/AGF0JC/P3dfE+OgCT). Pollen analysis from marine sediments off the coast of Africa has confirmed this shift in vegetation, showing increased percentages of grassland and woodland pollen during the AHP[^2^](https://paperpile.com/c/AGF0JC/x2S0j) (**Supp. Fig. 1.1**). Moreover, the increased rainfall during the AHP significantly impacted the lakes, rivers, and overall ecosystems. The hyper-arid Sahara transformed into a landscape dotted with perennial lakes and rivers, supporting abundant aquatic life and attracting various species of large mammals[^5^](https://paperpile.com/c/AGF0JC/P3dfE). Evidence for this can be found in paleolake sediments and fossil records, which reflect a high level of biodiversity[^7^](https://paperpile.com/c/AGF0JC/DAO70).


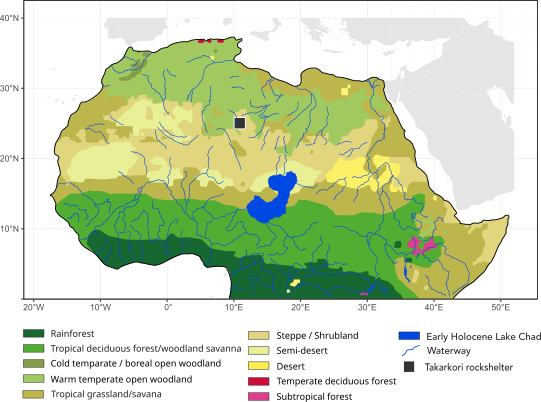


**Supplementary Figure 1.1:** Map of early Holocene Northern Africa, showing simulated biome distribution based on the CARAIB model. The location of the Takarkori rockshelter site is marked with a black square. (Adapted from[^4,8^](https://paperpile.com/c/AGF0JC/QbemM+Yiag))

### The termination of AHP

The AHP ended due to a shift towards drier conditions, often referred to as the "African Humid Period Termination". This change was triggered by shifts in the Earth's orbit over time, which reduced the amount of sunlight reaching the Northern Hemisphere in the summer. As a result, monsoon rains became less intense, leading to a return to a drier environment[^1^](https://paperpile.com/c/AGF0JC/tpNO3). This quick drop in heavy seasonal rains led to a drying out of North Africa and the development of the Sahara Desert as we recognize it today.

## The role of climate on Holocene societies in the Tadrart Acacus

The AHP also significantly transformed the Tadrart Acacus region in southwestern Libya. This once-arid landscape flourished into vibrant grasslands and lakes during the AHP, promoting human habitation. The resulting resource abundance enabled the development of diverse subsistence strategies, such as hunting, gathering, and fishing[^9^](https://paperpile.com/c/AGF0JC/hD86q). Numerous archaeological sites in the Tadrart Acacus, including the well-known Takarkori rockshelter, provide evidence of these adaptations. For example, the well-preserved organic remains at Takarkori provide insight into the plant and animal resources utilized during this period. Additionally, evidence suggests an early transition from wild cereal gathering to cultivation, possibly in response to the favorable conditions of the AHP[^10^](https://paperpile.com/c/AGF0JC/j1s8t). With the termination of the AHP and the return of arid conditions, the human groups in the Tadrart Acacus region had to adapt to the decline of previously abundant resources. This period saw the full establishment of pastoralism and reliance on domesticated animals[^10–12^](https://paperpile.com/c/AGF0JC/hOAVC+j1s8t+bsKd).

## Takarkori rockshelter

The Takarkori rockshelter, situated within the Libyan Tadrart Acacus mountain range near the Algerian border, overlooks the wadi floor from roughly 100 meters up, covering an area of about 2200 m^2^. This site is a critical junction in the southwest Libyan Tadrart, forming a natural connection between the Tanezzuft valley and the Acacus ranges (**Supp. Fig. 1.2, Supp. Fig. 1.3 and Supp. Fig 1.4A**). It is also in proximity to an ancient reservoir, which was supplied with water until the late Middle Holocene[^8^](https://paperpile.com/c/AGF0JC/Yiag). The site has been investigated during four excavation campaigns (2003-2006), covering a total area of 143 m^2^ divided into a 1x1 m square grid. The stratigraphic excavation in extension was carried out in association with the digital recording of relevant finds and features through ETS (Electronic Total Station). Four excavation areas were established: the Main Sector (TK-MS), the largest covering 117 m^2^; the Northern Sector (TK-NS), a smaller trench measuring 4x2 m, revealing the deepest levels of occupation, reaching approximately 1.60 meters below the walking surface; the Southern Sector (TK-SS), a 4x5 m area corresponding to the excavation of a conical tumulus with likely funerary significance; and the Western Sector (TK-WS), a 3x3 m trench located beyond the shelter's drip-line, featuring a notable concentration of surface artifacts[^13^](https://paperpile.com/c/AGF0JC/X7Bm) (**Supp. Fig 1.4B**). The site's chronology spans nearly 4000 years and is secured by an extensive radiocarbon dating program, allowing a breakdown of the sequence in major phases and relative sub-phases[^14^](https://paperpile.com/c/AGF0JC/1h7J) (**Supp. Fig 1.4C**). Initial occupation dates to the Early Holocene Late Acacus horizon (10,200-8,000 calBP), which is further divided into three sub-phases: LA1, LA2, and LA3. These phases featured stone structures, fireplaces, grinding stones, and pottery fragments, reflecting a semi-residential settlement pattern with extensive regional and inter-regional connections[^10,11,15,16^](https://paperpile.com/c/AGF0JC/j1s8t+hOAVC+QcLx+6CKi). The Early Holocene use was followed by a prolonged Pastoral Neolithic occupation (EP, MP, LP phases) marked by cattle and ovicaprine herders in the Middle and Late Holocene. The Early Pastoral phase (8,300-7,300 calBP), concomitant with the 8.2k event, with two sub-phases EP1 and EP2, featured fireplaces, stone structures, and human graves, and saw the onset of herding practices, among the earliest recorded in Africa[^17^](https://paperpile.com/c/AGF0JC/0tGG). The Middle Pastoral phase (7,100-5,600 calBP), divided into MP1 and MP2, emphasized cattle herding and the utilization of secondary products within a complex settlement strategy, including transhumance and periodic occupation of mountainous area. The Late Pastoral phase (LP1-5900-4300) is distinguished by specialized goat herders, as evidenced by extensive dung accumulations, small hearths, and other facilities indicating sporadic nomadic pastoralist occupation. The funerary area of 8 m^2^ was uncovered in the most recessed part of the shelter in the MS, near the rock wall. It hosted the remains of 12 human burials of different chronologies, from Late Acacus to Late Pastoral. Other fragmentary remains of 3 more individuals were recovered from other areas of the site. The inhumations differ in preservation pattern, stratigraphic features, and formal layout: from small stone accumulations to cover the Early Pastoral burials to earthen pits dug in the sediment and covered by the same soil characterizing the Middle and Late Pastoral burials[^18,19^](https://paperpile.com/c/AGF0JC/0xmvE+bNAT). The two specimens, TK H1 (TKH001) and TK H9 (TKH009), belong to the Middle Pastoral horizon. Funerary practices in the Acacus region appear to segregate the burial locations based on sex/age dimension, with rockshelter and mountainous sites seemingly reserved for women and children while men were likely interred in open-air sites[^18^](https://paperpile.com/c/AGF0JC/0xmvE). TK H1 is a female aged between 30 and 40 years at death. The body is naturally dried out and buried in a shallow pit dug in the organic sands and laid on the left side in a contracted, tightly bent posture, oriented North-South and facing East. While many skeletal districts retained anatomical connections, the skeleton was incomplete, with missing parts of the upper limbs, lower limbs, and elements of the thoracic/abdominal region. Dislocations and losses are likely the result of post-depositional processes, both recent and *ab antiquo,* the latter connected to the deposition of TK H9. This is a mature female individual, partially dried. Skeletal remains are represented only by some anatomical districts, notably the pelvis and lower limbs. No grave goods were associated with these burials but for a fictile zoomorphic figure resembling a cow (associated with TK H1), fitting the symbolic and ideological framework of this cultural phase characterized by the centrality of cattle and herding practices as also evidenced by the wealth of rock art and animal burials widespread in this and the neighboring regions[^20,21^](https://paperpile.com/c/AGF0JC/rQUC+JiTB).


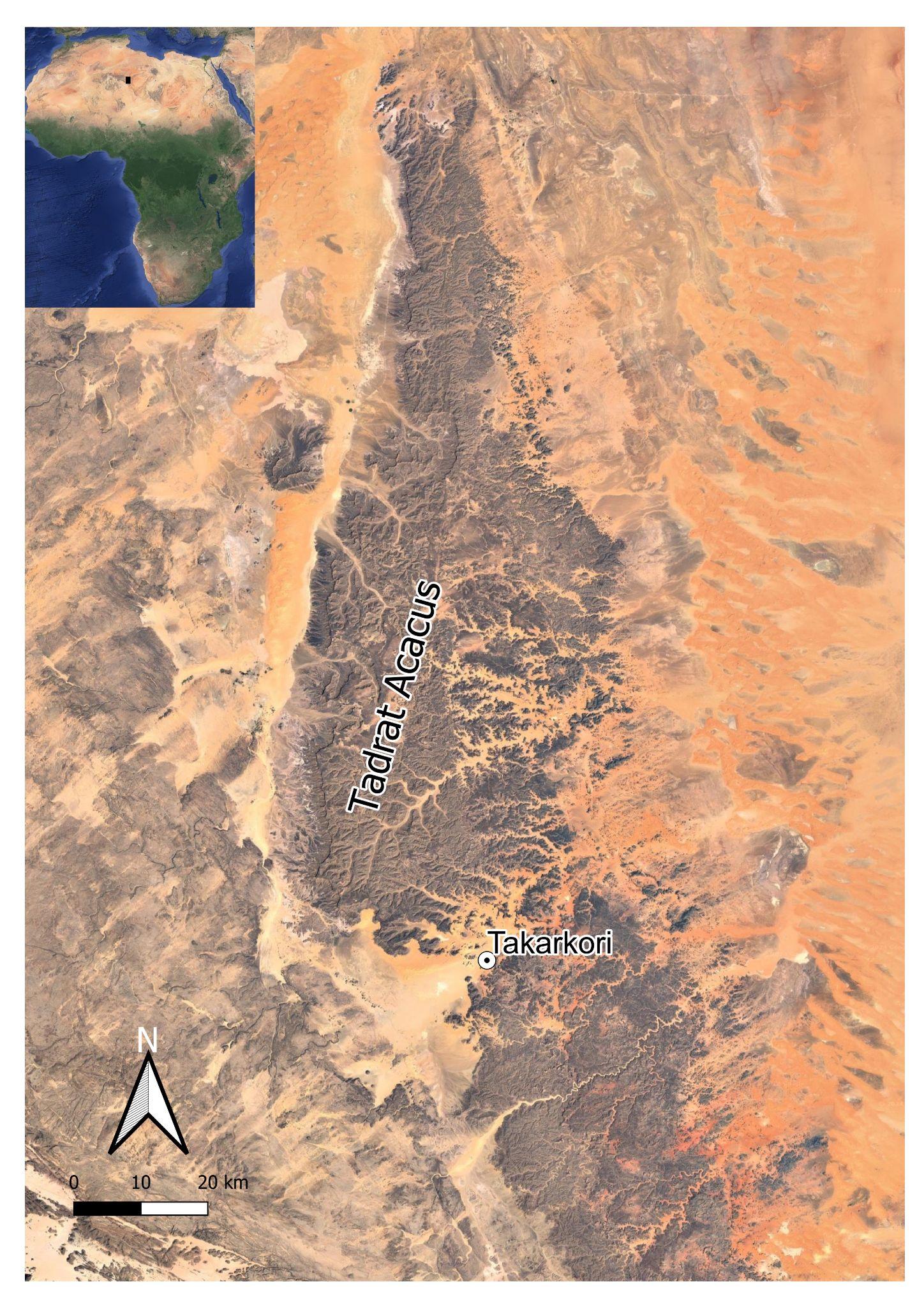


**Supplementary Figure 1.2:** Satellite image of the Tadrart Acacus mountains and the location of the Takarkori rockshelter (QGIS (v.3.34.10) elaboration; Google Earth base map).


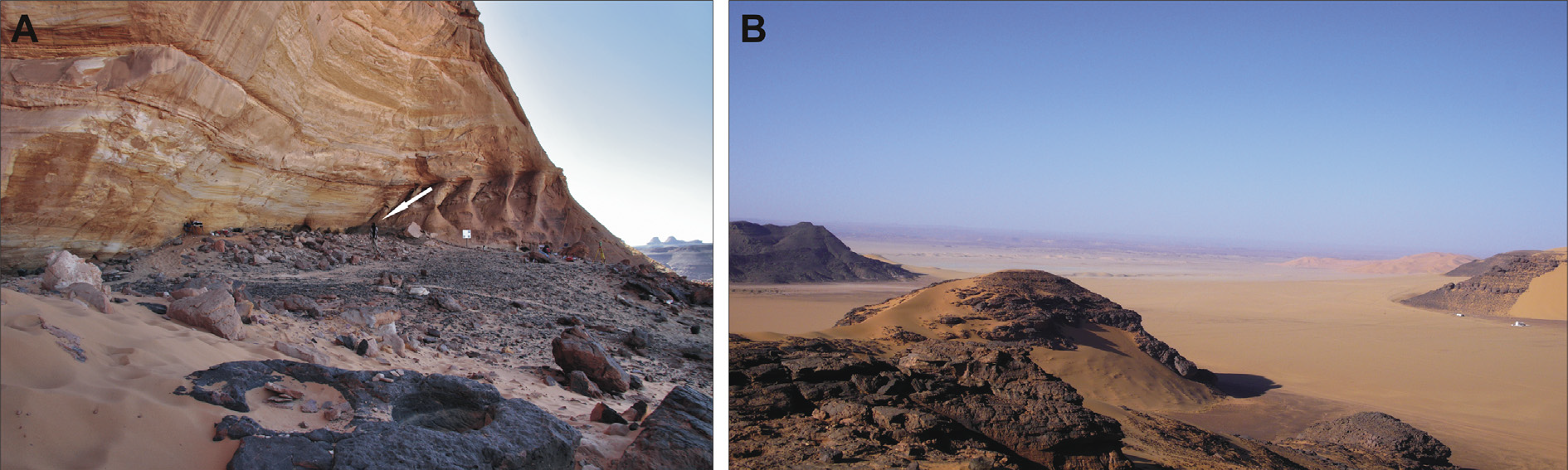


**Supplementary Figure 1.3:** **A)** Overview of the Takarkori rockshelter with an archaeologist indicated for scale (arrow). **B)** Landscape panorama from the exterior of the rockshelter, illustrating the site's surroundings. The location of the ancient swamp is visible in the lower section of (B), with the Algerian Tassili seen in the distance. Adapted with permission from[^6^](https://paperpile.com/c/AGF0JC/OgCT)**.**

**
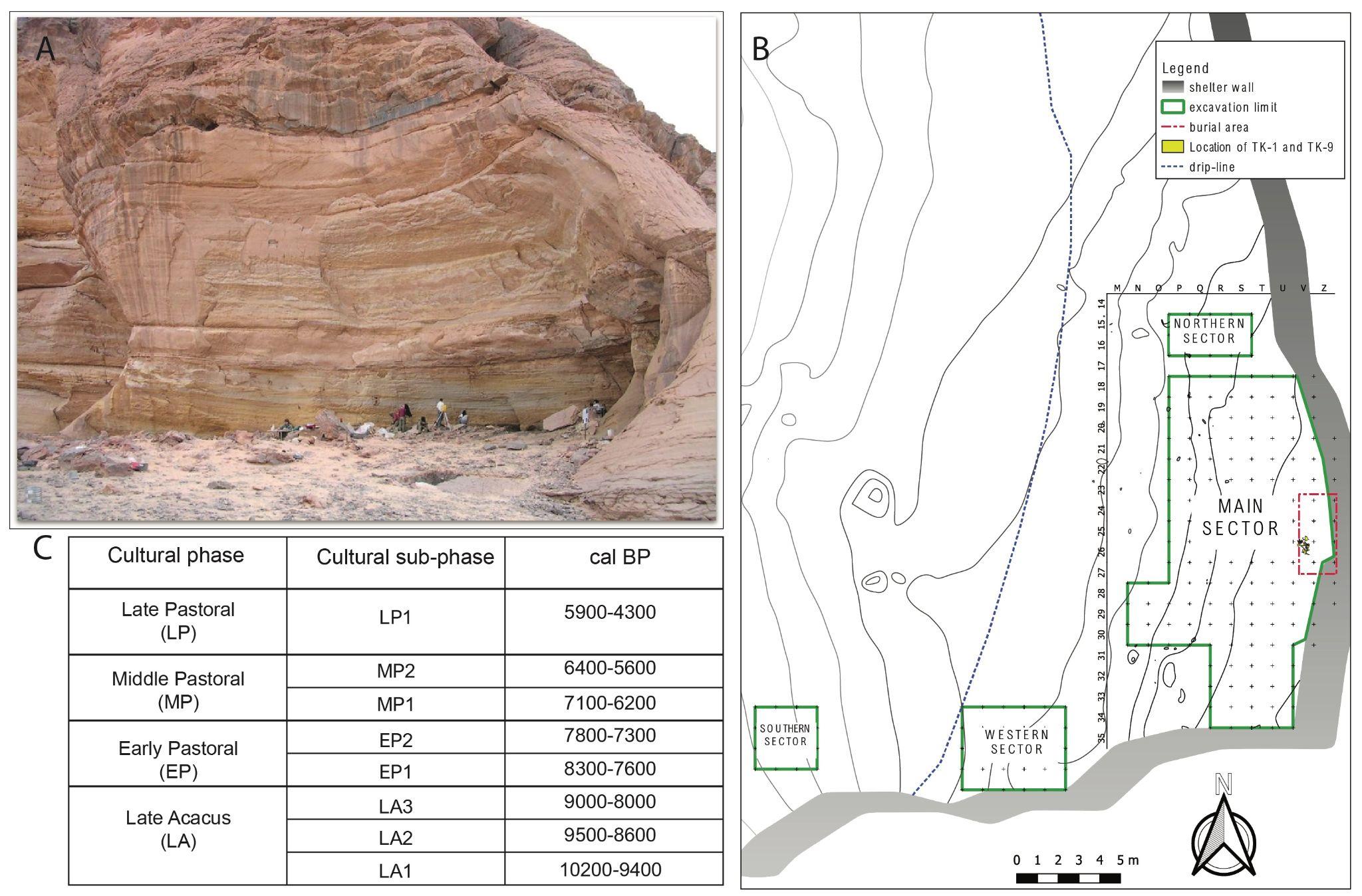
**

**Supplementary Figure 1.4:** Temporal and spatial overview of the Takarkori site. **A)** The Takarkori rock shelter looking East into the main sector of the shelter (photograph from the Archive of the Archaeological Mission in the Sahara, Sapienza University of Rome. Copyright: S. di Lernia); **B)** Excavation layout with sectors TK-MS, TK-NS, TK-SS, and TK-WS, showing areas of interest and depth of occupation; **C)** Site chronology spanning 4,000 years, detailing the shift from Early Holocene habitation to Pastoral Neolithic herding practices and burial customs (modified after[^13,14^](https://paperpile.com/c/AGF0JC/1h7J+X7Bm)). The calibrated dates (95.4%) express the maximum chronological range, therefore overlaps are statistically possible. OxCal online version 4.4 and IntCal20 were used as calibration curve[^22,23^](https://paperpile.com/c/AGF0JC/eNjj+9AsM).

### **Burial and sampled individuals**

The individuals were chosen for the apparent good state of preservation of skeletal parts, as well skin and other organic remains. Below are the main features of the RS H1 and RS H9 burials of the sampled individuals, from which the TKH001 and TKH009 samples were respectively obtained:

**RS H1** (ref [^18^](https://paperpile.com/c/AGF0JC/0xmvE))

This burial contains the remains of a woman aged between 30-40 years[^18^](https://paperpile.com/c/AGF0JC/0xmvE) (**Supp. Fig. 1.5**). The skeleton was found in a shallow pit filled with loose organic sand, indicating significant disturbance over time. The body was positioned on its left side in a north-south orientation, with both arms and the left leg retaining their articulations. However, the right leg was dislocated, and the right humerus, fibula, and some thoracic vertebrae were missing, likely due to post-depositional disturbances. Notably, a small clay figurine of a zoomorphic figure (possibly a cow) was found within the grave, suggesting an association with the burial. Six specimens were collected for aDNA analysis, including one tooth and fragments of bones and skin from different anatomical regions. Only the root of the tooth showed a degree of molecular preservation sufficient to completely reconstruct the mitochondrial genome[^24^](https://paperpile.com/c/AGF0JC/68BW) and be selected as a potential candidate for genomic analysis.The remains date to 6,090 ± 60 BP (GX-30324-AMS) (7,158–6,796 years calBP, 95.4% probability)^^[[1]](#footnote-1)^^, corresponding to the Middle Pastoral phase.

**RS H9** (ref [^18^](https://paperpile.com/c/AGF0JC/0xmvE))

This burial site contains the remains of a mature individual (30-35 years), likely a female. Only a complete and articulated pelvis and portions of the lower limbs were preserved, found beneath RS H1. The right leg was extended and the left leg was twisted beneath the pelvis, indicative of post-depositional disturbance or secondary deposition. Two fragments of fibula and a skin fragment were previously analysed for the mitochondrial genome[^24^](https://paperpile.com/c/AGF0JC/68BW), and only the bone samples resulted as suitable for the analysis of the nuclear genome. Radiocarbon dating places this burial within the Middle Pastoral period, ca. 5,600 ± 70 BP (GX-31077) (6,555–6,281 years calBP (95.4% probability).


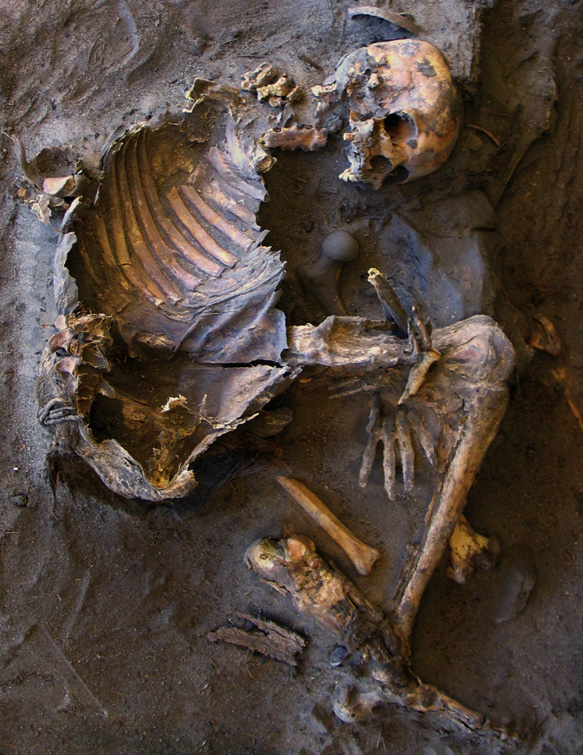


**Supplementary Figure 1.5:** Three-dimensional visual representation of RS H1 obtained with MSR orthorectification and vectorial morphing. Adapted with permission from[^18^](https://paperpile.com/c/AGF0JC/0xmvE).

# Supplementary Note 2: Ancient DNA processing and population genetic analyses

### Sampling and DNA extraction

A tooth root from individual TKH001 (TK RS H1) and two fibula fragments from TKH009 (TK RS H9) were collected for our genetic analyses. The sampling and DNA extraction procedures were carried out in a specialized ancient DNA facility at the Laboratory of Molecular Anthropology and Paleogenetics at the University of Florence, following the process described in reference[^24^](https://paperpile.com/c/AGF0JC/68BW). To monitor for potential contamination, blanks were used as negative controls throughout the experiments. The samples were prepared for DNA extraction by using a disposable-tip dentist drill to remove the surface layer, which was then followed by exposure to UV light (λ = 254 nm) for 45 minutes. 100 mg of powder was collected from the dentine of the tooth root for TKH001 and from the compact section of the fibula for TKH009. DNA was extracted using a previously published protocol[^25^](https://paperpile.com/c/AGF0JC/x6t8G).

### Double-stranded library preparation

The double-stranded library preparation for the TKH009 specimen was carried out at the Laboratory of Molecular Anthropology and Paleogenetics at the University of Florence. The sample was converted into a DNA library using 30 μl of DNA extract, following a double-stranded DNA protocol[^26^](https://paperpile.com/c/AGF0JC/wmL4Q). It was tagged with a unique combination of two indices. Negative controls were evaluated using both qPCR and the Agilent 2100 Bioanalyzer DNA 1000 chip. Following the adapter ligation process, the blanks showed a concentration 4–5 orders of magnitude lower compared to the biological sample, indicating minimal contamination in the experiment. Furthermore, only adapter-indexes dimers were detected after analyzing the indexing PCR products. The DNA library was not subjected to treatment with uracil-DNA-glycosylase (UDG) to prevent any loss of DNA molecules and reduction in library complexity[^27^](https://paperpile.com/c/AGF0JC/1rhf6).

### Single-stranded library preparation

For the TKH001 specimen, a single-stranded library preparation was performed using 30 μl of DNA extract. This method is particularly effective for capturing short fragments of highly degraded DNA molecules, thereby increasing the final genomic coverage[^28^](https://paperpile.com/c/AGF0JC/EDWGA). The experiment was carried out at the Ancient DNA Core Unit facility at the MPI-EVA in Leipzig, using an automated version of the single-stranded protocol described by[^28^](https://paperpile.com/c/AGF0JC/EDWGA) and an Agilent Technologies Bravo NGS Workstation. This library preparation did not involve any UDG treatment, similar to the earlier double-stranded library for TKH009.

### Shotgun sequencing and target enrichment of nuclear DNA

The prepared libraries were shotgun sequenced to a depth of 3-5 million reads, using a 75 bp single-read configuration on an Illumina HiSeq 4000. The output reads were demultiplexed according to their expected index combinations and filtered for a length exceeding 30 bp. These reads were subsequently aligned to the human reference genome hg19, applying a mapping quality filter (MQ) of 30. This analysis was run with the software EAGER v.1.92.55[^29^](https://paperpile.com/c/AGF0JC/3oZyA). The DNA reads demonstrated a damage pattern typical of ancient DNA (**Supp. Fig. 2.1 and Supp. Fig. 2.2**) and an endogenous DNA content (**Supp. Table 2.1**) adequate for in-solution capture. After further amplification with the IS5/IS6 primers, the double-stranded library from the TKH009 specimen was in-solution to oligonucleotide probe sets (from Agilent) for two rounds of “1240k” capture[^30^](https://paperpile.com/c/AGF0JC/ACGf), targeting 1,237,207 single nucleotide polymorphisms (SNPs).

For the TKH001 specimen, the single-stranded library underwent two rounds of capture using Twist Ancient DNA probes[^31^](https://paperpile.com/c/AGF0JC/eUQg) targeting 1.4 million SNPs, including all 1,233,013 SNPs from the 1240k panel. Additionally, it underwent two rounds of enrichment to target a set of 1,749,385 SNPs that form the “archaic ancestry” panel (Panel 4 in [^32^](https://paperpile.com/c/AGF0JC/oH7eX)). All captured libraries were sequenced to 20 million reads and subsequently demultiplexed.


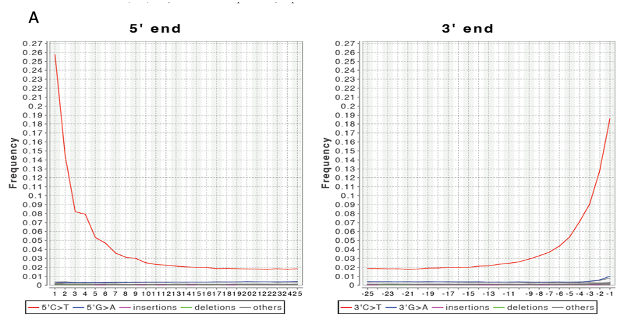


**Supplementary Figure 2.1**: DNA damage in the single-stranded TKH001 shotgun library.

**
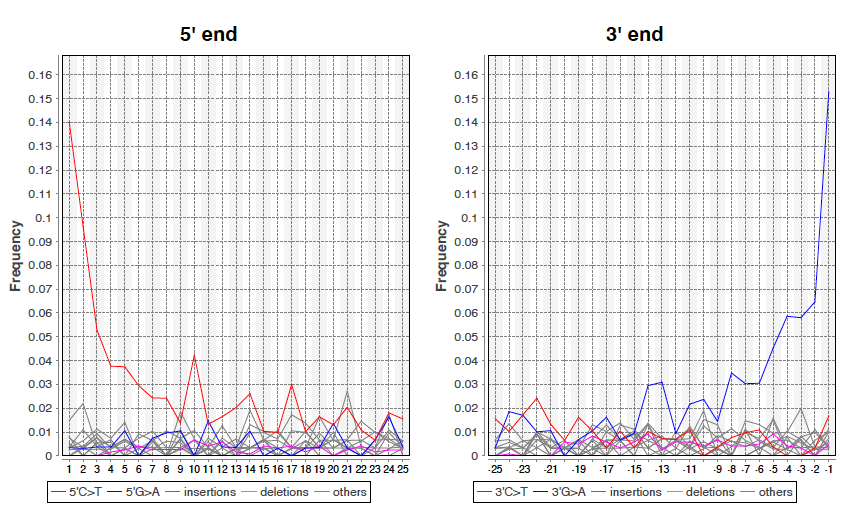

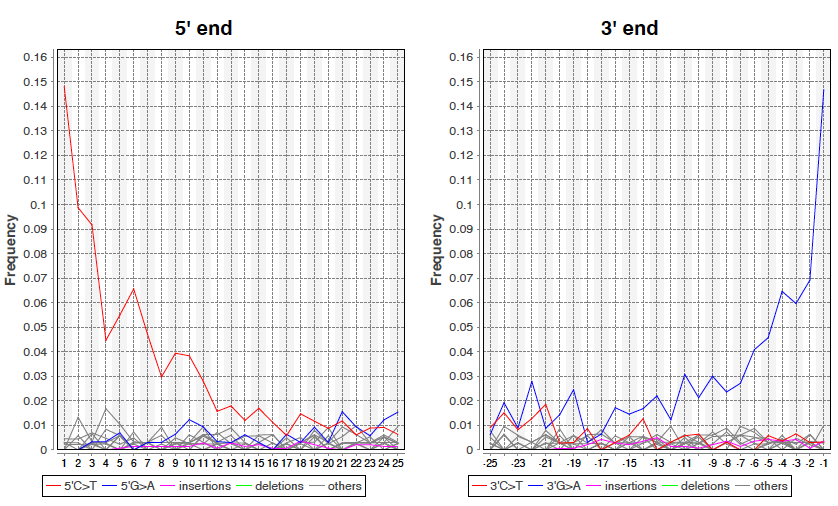
**

**Supplementary Figure 2.2**: DNA damage patterns observed in the two shotgun libraries from TKH009, prepared using a double-stranded protocol.

**Supplementary Table 2.1:** Summary of sequencing data and endogenous DNA content for each library.

| **Individual ID, sample type** | **Shotgun library ID** | **Library type** | **Sequencing setup, Read length** | **Endogenous DNA (%)** |
| --- | --- | --- | --- | --- |
| TKH001, tooth root | TKH001.A0101.SG1.1 | single-stranded | single-stranded, 75bp | 1.363 |
| TKH009, fibula fragment | XXX001.A18276.SG1.1 | double-stranded | paired-end, 50bp | 0.085 |
| TKH009, fibula fragment | XXX001.A18277.SG1.1 | double-stranded | paired-end, 50bp | 0.249 |

## Population genetic analyses

### Analyzing enriched libraries

The resultant Fastq files were aligned to the human genome hg19 using BWA software[^33^](https://paperpile.com/c/AGF0JC/XM78f) v.7.12 as implemented in EAGER. For this process, we applied an MQ quality filter of 25, a seedlength of 16500, and a -n setting of 0.01. Adapters and duplicates were identified and removed with AdapterRemoval[^34^](https://paperpile.com/c/AGF0JC/DK0o) v.2.3.l and DeDup v.0.12.1, respectively. Damage calculation was performed using DamageProfiler v.1.1[^35^](https://paperpile.com/c/AGF0JC/m1s4).

### Spurious alignment estimate

To maximize the retrieval of usable reads from our samples, we used a sample-specific minimum length cutoff. This cutoff, applied during the EAGER run, removes shorter sequences more likely to be spurious. This technique is based on the Spurious Alignment Estimate (SpAl) method introduced by[^36^](https://paperpile.com/c/AGF0JC/n4sZ). The specific length cutoffs applied to each library are listed in **Supp. Table 2.2.** These cutoffs are expected to lower the probability for spurious alignment to a genome-wide average of only 1-10%.

**Supplementary Table 2.2:** 1240K captured libraries with respective library setup and length cutoff used.

| **1240k captured library** | **Library setup** | **Length cutoff used** |
| --- | --- | --- |
| TKH001 | single-stranded | 30 bp |
| TKH009-1 | double-stranded | 26 bp |
| TKH009-2 | double-stranded | 24 bp |

### Ancient DNA contamination

We evaluated nuclear contamination in single-stranded libraries with the software AuthentiCT[^37^](https://paperpile.com/c/AGF0JC/Snl0k) v.1.0. This tool estimates the degree of contamination from modern DNA in ancient DNA samples based on post-mortem damage patterns. Congruently with Vai et al.[^24^](https://paperpile.com/c/AGF0JC/68BW), who ran Schmutzi on the mtDNA captured double-stranded libraries of both the TKH001 and TKH009 individuals, we find no indications of possible contamination of the nuclear genome (**Supp. Table 2.3**).

Runs of homozygosity (ROH) on autosomes, which are effectively haploid, offer a method for estimating ancient DNA contamination, similar to the approach based on the male X chromosome. This technique applies to individuals of both sexes. Long ROHs are typically uncommon but are more frequent in descendants from closely related individuals or in populations with small effective sizes, like typical in hunter-gatherers. In these cases, the individuals often possess a sufficient number of detectable ROHs to robustly estimate autosomal contamination. This approach was implemented in the 'hapCon_ROH' software (<https://haproh.readthedocs.io/en/latest/hapROH_with_contamination.html>) and is described in detail in Supplementary Section 2 in Posth et al[^38^](https://paperpile.com/c/AGF0JC/Nutc). We ran HapCon_ROH on TKH001 data, and estimated its contamination to be 1.7% (0.9-2.6%), based on a total inferred ROH of 34.5cM.

**Supplementary Table 2.3:** Contamination estimates for each library

| **Individual ID** | **Library setup** | **AuthentiCT estimate±SE** | **Schmutzi estimate (%), first iteration (low - high)**[^24^](https://paperpile.com/c/AGF0JC/68BW) | **Schmutzi estimate (%), final iteration (low - high)** | **hapCon_ROH(%), (95%CI)** |
| --- | --- | --- | --- | --- | --- |
| TKH001 | ssLib | 1% ± 2.72% | NA | NA | 1.7 (0.9-2.6) |
| TKH009 | dbLib | NA | 0 (0-0.005) | 0 (0.01-0.02) | Not sufficient data |
|  | dbLib | NA | 0 (0-0.005) | 0 (0.01-0.02) |  |

###

### Genetic sex determination

Based on the misincorporation pattern of the double-stranded library, two base pairs were trimmed from both ends of all reads after alignment in the double-stranded 1240k-captured sequence to reduce damage-induced bias. This was followed by a comparison of the relative coverages of the X and Y chromosomes to that of the autosome, yielding the X- and Y-rates, respectively. Samples were classified as 'male' if their X to autosomal coverage ratio was ≤ 0.75 and Y to autosomal coverage ratio was ≥ 0.25. Conversely, 'female' classification was applied when X to autosomal ratio was > 0.75 and Y to autosomal ratio was < 0.25. The high X-rates (0.789 for TKH001 and 0.803 for TKH009) and low Y-rates (0.042 for TKH001 and 0.056 for TKH009) suggest that both these individuals had two X chromosomes and no Y chromosome, i.e. both individuals were genetically females, a finding consistent with the anthropological sex assessment of the skull (H1) and pelvis (H1 and H9).

### Genotyping

The double-stranded 1240k-captured sequences from TKH009 were genotyped for the previously mentioned panel of 1,237,207 SNPs. For this process, we separately genotyped the untrimmed and 2bp-trimmed BAM files using the mpileup command in Samtools v.1.3, followed by pileupCaller from SequenceTools v.1.4.0.2 (<https://github.com/stschiff/sequenceTools>). This software treats the human genome as pseudo-haploid and calls one read at random. We then combined the untrimmed and trimmed genotypes, keeping only the transversions from the untrimmed genotype and transitions from the trimmed genotype to fully utilize information from the trimmed ends. The merged TKH009 libraries produced 22,484 SNPs on the 1240k panel and 11,945 SNPs on the HO panel.

For the single-stranded TKH001 library captured with Twist probes, the sequences were genotyped on the sites of the 1240k panel, resulting in 881,765 SNPs and 572,130 SNPs on the HO panel (**Supp. Table 2.4**). We used the mpileup command from Samtools v.1.3 and pileupCaller in the single-stranded mode, which helps eliminate damage by disregarding reads aligning to the forward strand at C/T polymorphisms and to the reverse strand for G/A polymorphisms.

The TKH001 and TKH009 libraries were merged after observing a very similar projection in the PCA, where both individuals formed a tight cluster with overlapping 95% confidence interval ellipses (as detailed in the Data Validation below). Additionally, each of the TKH001 and TKH009 datasets produced nearly consistent results on the *f3-*outgroup test, supporting their grouping into one entity for subsequent analyses (**Supp. Fig. 2.3**).

**Supplementary Figure 2.3:** Outgroup-$f$_3_(Chimp; X1, X2) statistics, where X represents relevant ancient populations from Africa and the Near East. Populations are clustered based on shared genetic drift, with TKH001 and TKH009 showing nearly consistent results.
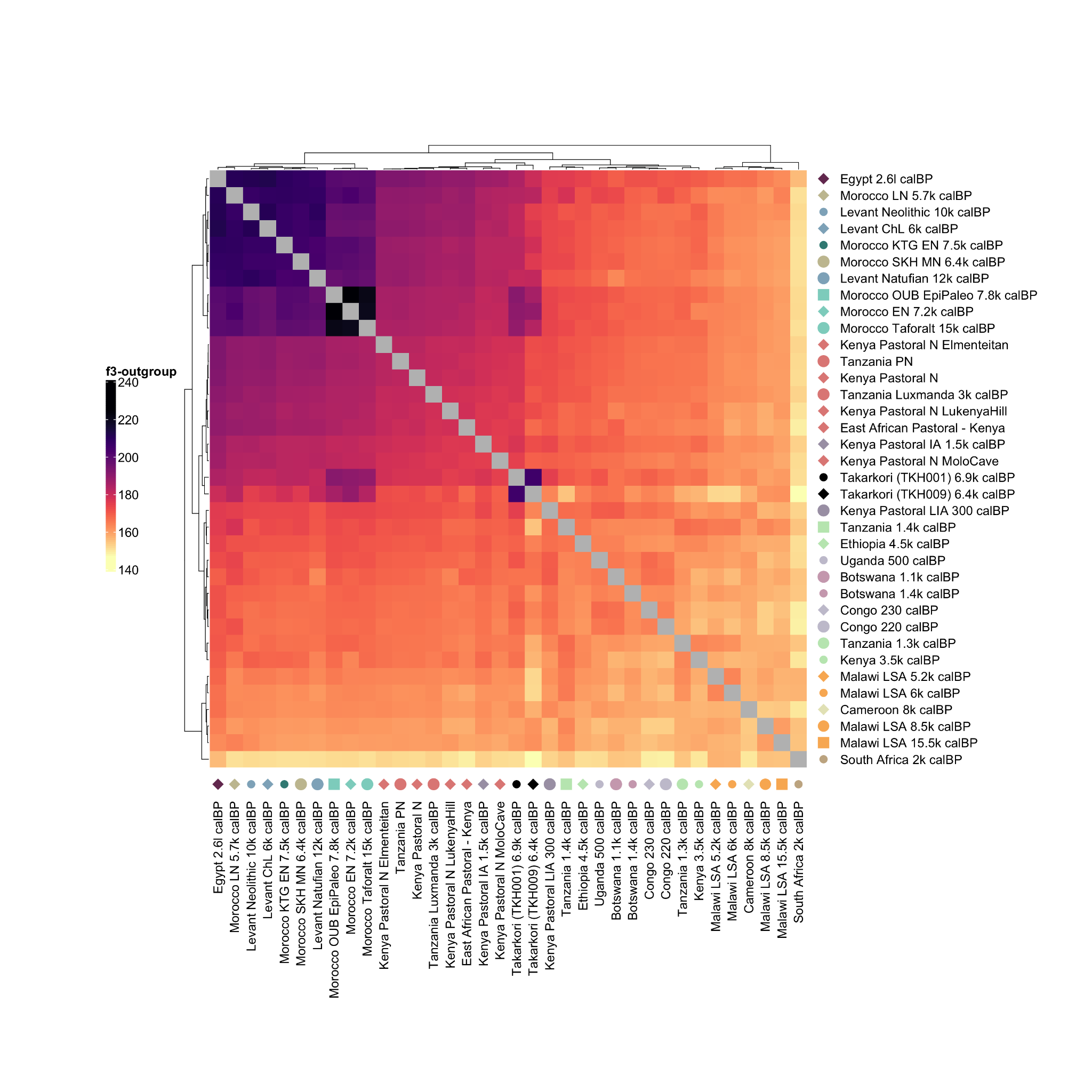


### Data validation

To explore and mitigate potential artifacts resulting from possible post-mortem damage or contemporary contamination, we generated additional filtered genotype datasets for the TKH001 sample:

1. Data using only the single-stranded library

2. Data restricted to transversions only in the single-stranded library

3. Data filtered with PMDtools v.0.6, applying a PMD score restriction of 3[^39^](https://paperpile.com/c/AGF0JC/P4f5U)

The resulting SNP coverage from each version is outlined in **Supp. Table 2.4.**

**Supplementary Table 2.4**: SNPs coverage obtained from each version of genotyped data.

| **Data** | **Total SNPs typed on 1240k panel** | **Total SNPs typed on HO panel** |
| --- | --- | --- |
| TKH001_ssLib | 881,765 | 572,130 |
| TKH001_transversion | 227,736 | 97,131 |
| TKH001_PMD_filtered | 440,569 | 222,387 |

#### 95% Confidence interval ellipse for PCA

For each genotype dataset, along with the genotype of TKH009, ellipses with 95% confidence intervals (CI) were calculated in the PCA projection. The 95% confidence ellipse is a statistical tool implemented in EIGENSOFT v8.0, which helps visualize the spread and correlation of the data in two principal component dimensions. We found that all data versions above overlap (**Supp. Fig. 2.4**).

**Supplementary Figure 2.4:** PCA of TKH001 and TKH009 samples. The plot includes 95% confidence interval ellipses for data from the single-stranded library, transversions only, and PMD-filtered data for TKH001, along with the merged libraries for TKH009. Overlapping ellipses indicate consistent clustering across various Tarkakori datasets.
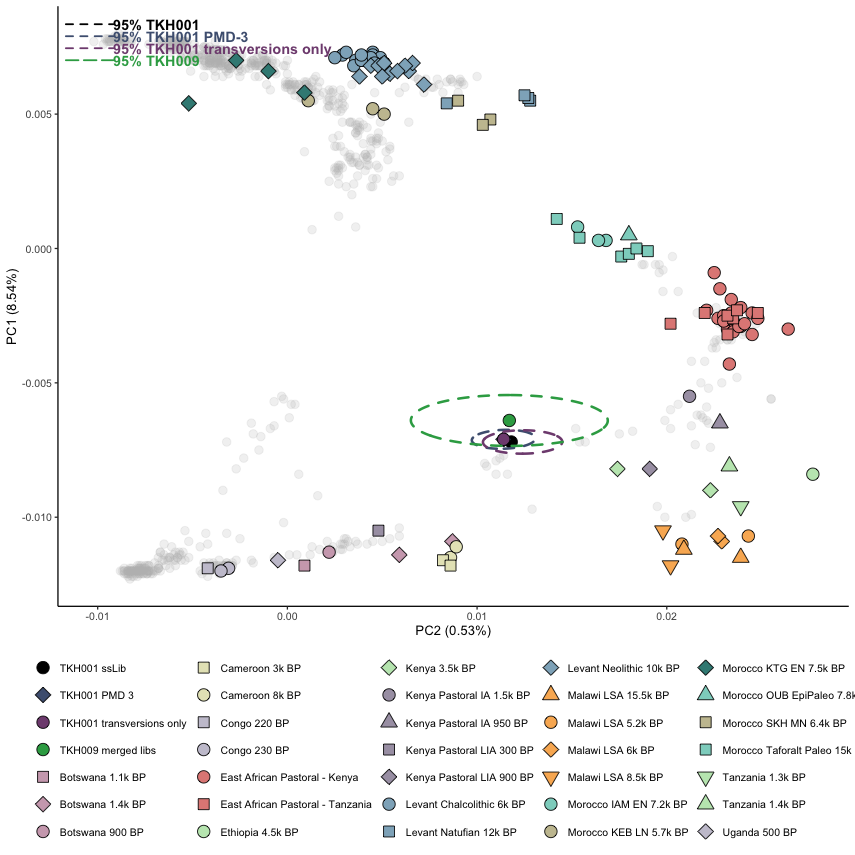


#### PCA without projection

A PCA was performed using 572,130 SNPs, limited to sites where the TKH001 individual Twist was covered. In this analysis, TKH001 was not projected but included directly in the computing populations group. Although directly included in the computation, TKH001 maintained the same placement as the previous projection-based analysis (**Supp. Fig. 2.5**), indicating consistent clustering patterns across both methods.

###

**Supplementary Figure 2.5: A)** PCA constructed on present-day populations in West Africa, the Sahel, East Africa, the Near East, and Southern Europe, based on 572,130 SNPs, including Takarkori (TKH001_twist) in the computation (black square). **B)** Geographic locations of Takarkori and relevant populations included in the analysis.
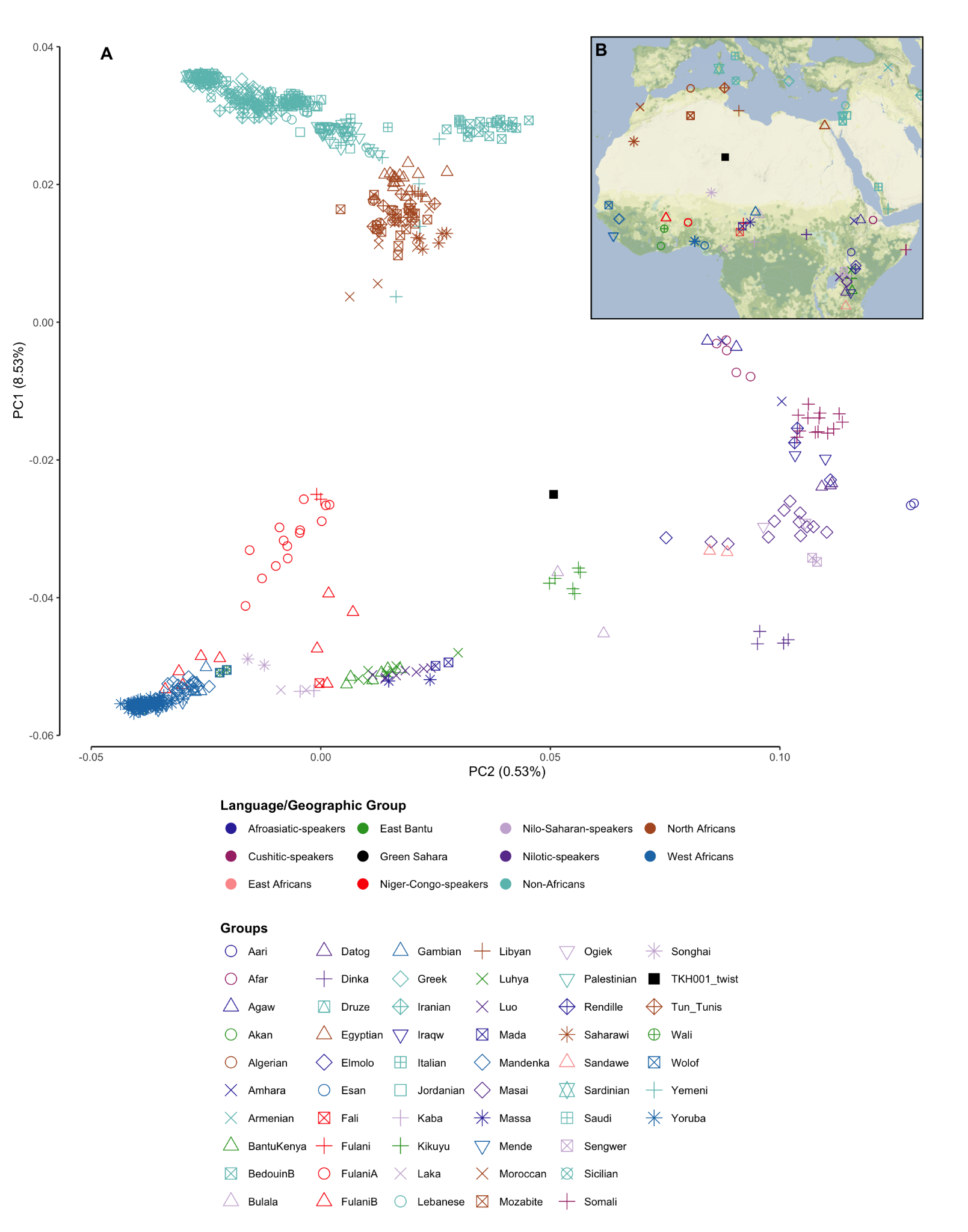


### Comparative dataset

We utilized the Allen Ancient DNA Resource (AADR)[^40^](https://paperpile.com/c/AGF0JC/5Zb2) v.54.1 in our study to generate a comparative dataset. In addition, we incorporated data from different groups of modern and ancient populations:

1. Modern populations from Fan et al. 2019[^41^](https://paperpile.com/c/AGF0JC/WM0m), retrieved from AADR v.51.1,
2. Modern populations as published in Lazaridis et al. 2014[^42^](https://paperpile.com/c/AGF0JC/PuJPM),
3. Modern populations from Pickrell et al. 2012[^43^](https://paperpile.com/c/AGF0JC/tur2).
4. Modern populations from D’Atanasio et al. 2023[^44^](https://paperpile.com/c/AGF0JC/4zFs)
5. Ancient groups from Simoes et al. 2023[^45^](https://paperpile.com/c/AGF0JC/mk0Ju)

#### Comprehensive SNPs dataset

The merging of these datasets resulted in 597,573 overlapped SNPs on the HO panel, which we refer to throughout as the “Comprehensive SNPs dataset”. The merging and retrieval of metadata, including 14C dating, geographical latitudes and longitudes for groups/individuals, and publication information were performed using trident CLI software v. 1.1.11.0 and the janno R package from the Poseidon framework (<http://www.poseidon-adna.org/#/>). **Supp. Data 1** lists the specific populations included in the analysis, their original publication sources, and the population labels used in our analysis. **All analyses in this study that include modern data are based on this Comprehensive SNP dataset.**

#### Reduced SNP dataset

To expand the dataset with modern North African data, we included 64 Tunisians and 59 Moroccans sampled from Marrakesh, Casablanca, Rabat, Fez, Nador, Oujda, Quarzazate, as described in Lucas-Sánchez et al. 2023[^46^](https://paperpile.com/c/AGF0JC/6Loh). This dataset, genotyped on HO panel, had a final count of 425,777 SNPs after quality control. However, upon merging this data with the previously mentioned datasets, only 410,367 overlapping SNPs remained, which we refer to as the “Reduced SNP Dataset”. **Due to this limited SNP overlap, we restricted the analysis of this dataset to PCA and Outgroup-*f*_3_ statistics, as discussed in the subsequent sections.**

#### The 184K SNP dataset

We sought to expand the dataset with modern populations from the Sahel/Savannah belt by including 327 individuals from 14 populations in present-day Senegal, Guinea, Chad, and Sudan, as described in Fortes-Lima et al. 2022[^47^](https://paperpile.com/c/AGF0JC/MuoE). This dataset was genotyped using the H3Africa array. However, after merging this data with the previously mentioned datasets, only 184,024 overlapping SNPs remained, which we refer to as the “184K SNP Dataset.” **Due to the limited SNP overlap, we restricted the analysis of this dataset to PCA and Outgroup-f3 statistics, as discussed in the subsequent sections.**

###

### Principal component analysis

Principal component analysis (PCA) was performed using smartpca v.16000 from the EIGENSOFT package v.8.0 (<https://github.com/DReichLab/EIG>). Initially, PCA was performed with 'lsqmode' and 'newshrink' enabled.

**PCA with Comprehensive SNP Dataset**

We constructed the principal components with the Comprehensive SNP Dataset using two sets: 1) a set of 795 present-day individuals from across the African continent, the Near East, and Southern Europe (**Supp.** **Data 2**), and 2) a set limited to African populations from West Africa, the Sahel, and East Africa, while retaining the Near Eastern and Southern European populations (**Supp. Data 3**). After constructing the PCs in both cases, we projected the relevant ancient genomes, including the Takarkori individuals, onto these axes. The African-wide PCA is illustrated in **Extended Data Fig. 2 and Supp. Fig. 2.6**, and the PCA for the northern half of Africa is shown in **Fig. 2A, Extended Data Fig. 1.**

**PCA with Reduced SNP Dataset**

Likewise, we constructed the PCA using the Reduced SNP Dataset with the same two sets of modern populations as mentioned above, but also including 123 individuals from Lucas-Sánchez et al. 2023[^46^](https://paperpile.com/c/AGF0JC/6Loh). The results of these PCAs are shown in **Supp. Fig. 2.7 and 2.8**. The overall shape of the African-wide PCA remained a similar triangle-like pattern as observed with the Comprehensive SNP Dataset. Noticeably, we observe that the Moroccan individuals from Ouarzazate (Mrc_Ouarzazate) and some Tunisian individuals cluster along the West-African to Near East cline, particularly extending from FulaniA to the North-African groups.

However, when focusing on the half-African PCA (**Supp. Fig. 2.8**), the separation becomes less distinct along both PC1 and PC2. The distinction between African and non-African populations along PC1 is less pronounced, and the separation between West/Sahelian and East African populations along PC2 is more blurred compared to the results from using the Comprehensive SNP Dataset (**Extended Data Fig. 1)**. Similar observation obtained when calculating the half-African PCA, but without the 123 individuals from Lucas-Sánchez et al. 2023[^46^](https://paperpile.com/c/AGF0JC/6Loh) (**Supp. Fig. 2.9**). The broader separation between different African regions (West, East, North) is slightly less distinct compared to the first PCA constructed with the Comprehensive SNP Dataset.

**PCA with the 184K SNP Dataset**

The African-wide and half-African PCAs constructed with this dataset are presented in **Supp. Fig. 2.10** and **Supp. Fig. 2.11**. The overall shape of the African-wide PCA retained a similar triangle-like pattern as observed with the Comprehensive SNP Dataset. The half-African PCA reflects the geographic distribution of the populations, with PC1 separating African from non-African populations and PC2 differentiating within Africa, particularly separating Sahel/West African populations from East African populations (Afroasiatic, Nilotic, and Nilo-Saharan speakers). Both Takarkori individuals maintained an intermediate position between Sahel/West African and East African populations, similar to what was observed with the Comprehensive SNP Dataset PCA.

**
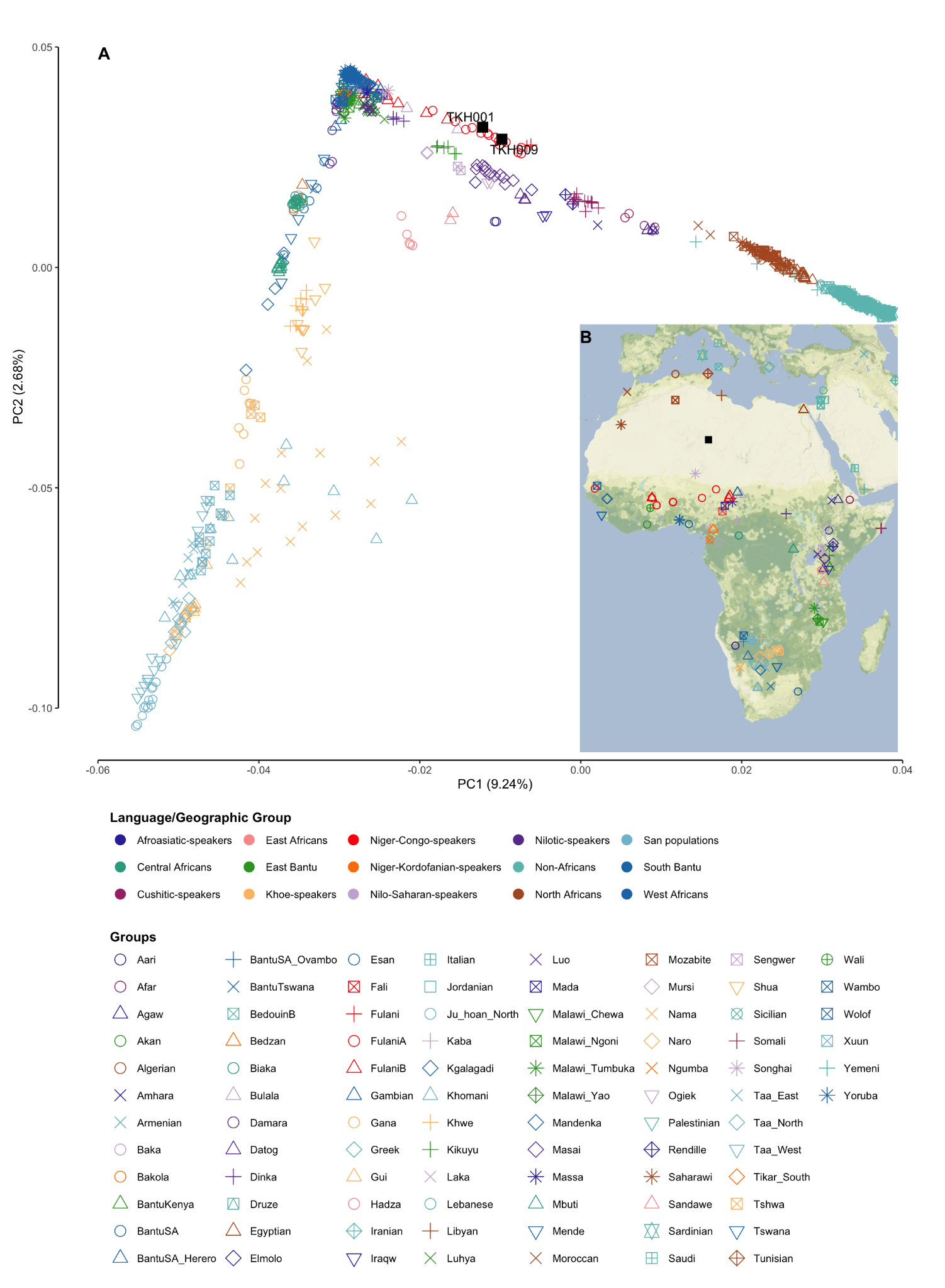
Supplementary Figure 2.6: A)** PCA constructed on present-day populations in Africa, the Near East, and Southern Europe based on the Comprehensive SNP dataset. Takarkori individuals projected and highlighted in black squares. **B)** Geographic locations of Takarkori individuals and relevant present-day populations included in our analysis.

**
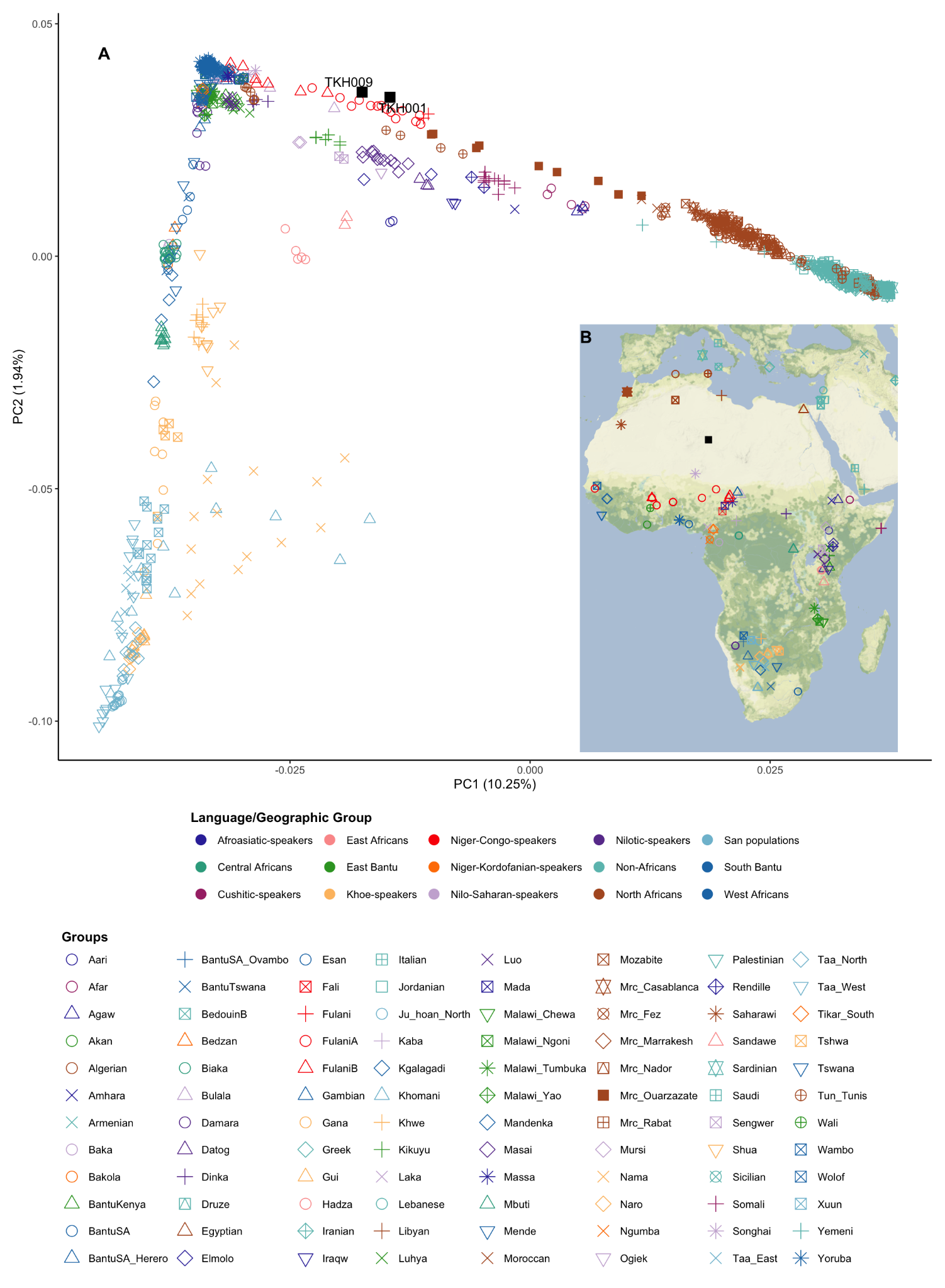
Supplementary Figure 2.7: A)** PCA constructed on present-day populations in Africa, the Near East, and Southern Europe based on the Reduced SNP dataset including the individuals from Lucas-Sánchez et al. 2023[^46^](https://paperpile.com/c/AGF0JC/6Loh) (prefixes “Mrc” for Moroccan and “Tun” for Tunisian). Takarkori individuals projected and highlighted in black squares. **B)** Geographic locations of Takarkori individuals and relevant present-day populations included in our analysis.

**Supplementary Figure 2.8: A)** PCA constructed on present-day populations in West Africa, the Sahel, and East Africa, as well as the Near East and Southern Europe, based on the Reduced SNP dataset including the individuals from Lucas-Sánchez et al. 2023[^46^](https://paperpile.com/c/AGF0JC/6Loh) (prefixes “Mrc” for Moroccan and “Tun” for Tunisian). Takarkori individuals are projected and highlighted in black squares. **B)** Geographic locations of Takarkori individuals and relevant present-day populations included in our analysis.
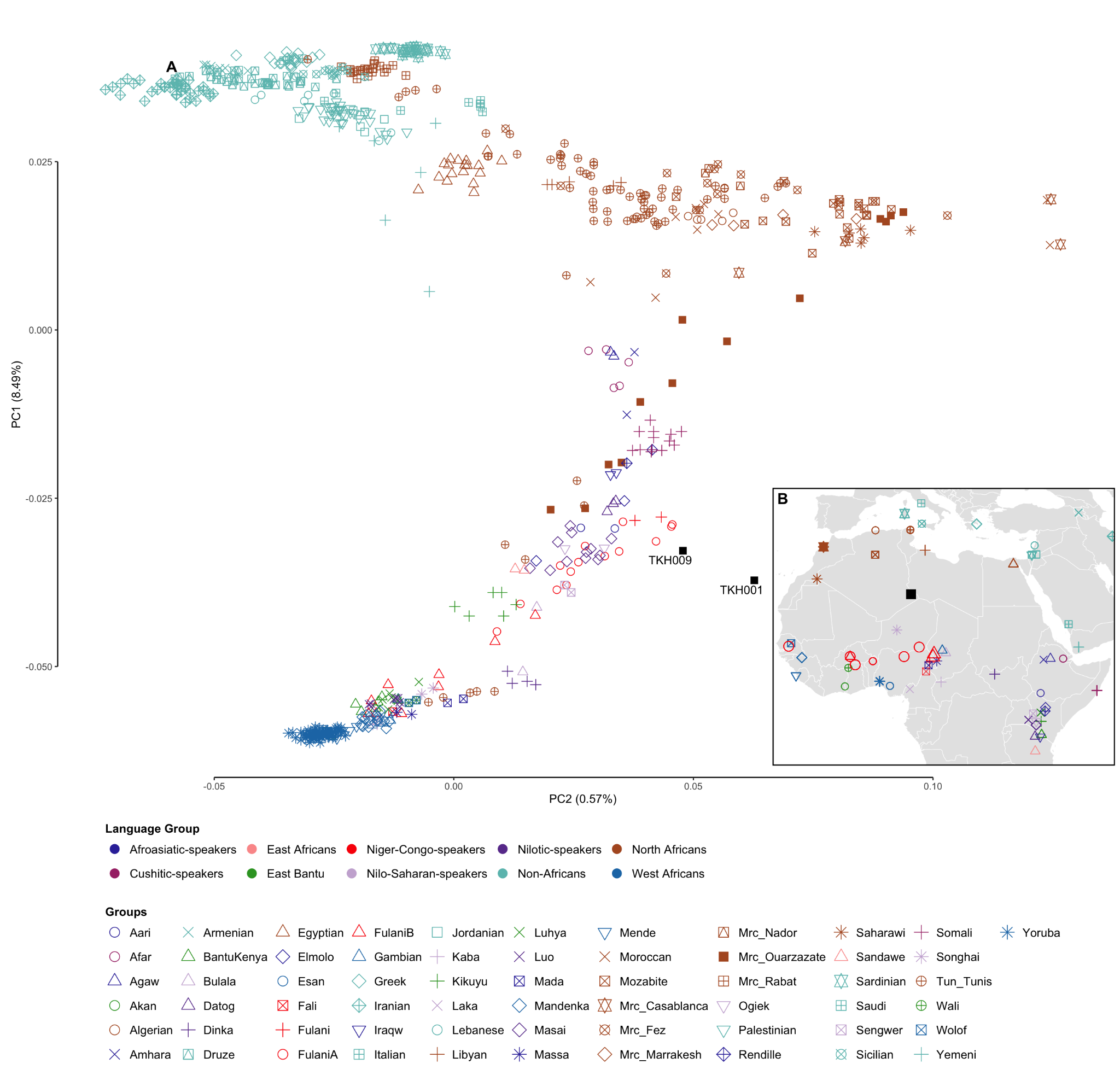


**Supplementary Figure 2.9:** PCA constructed on present-day populations in West Africa, the Sahel, and East Africa, as well as the Near East and Southern Europe, based on the Reduced SNP dataset including the individuals from Lucas-Sánchez et al. 2023[^46^](https://paperpile.com/c/AGF0JC/6Loh) (prefixes “Mrc” for Moroccan and “Tun” for Tunisian). Takarkori individuals are projected and highlighted in black squares.
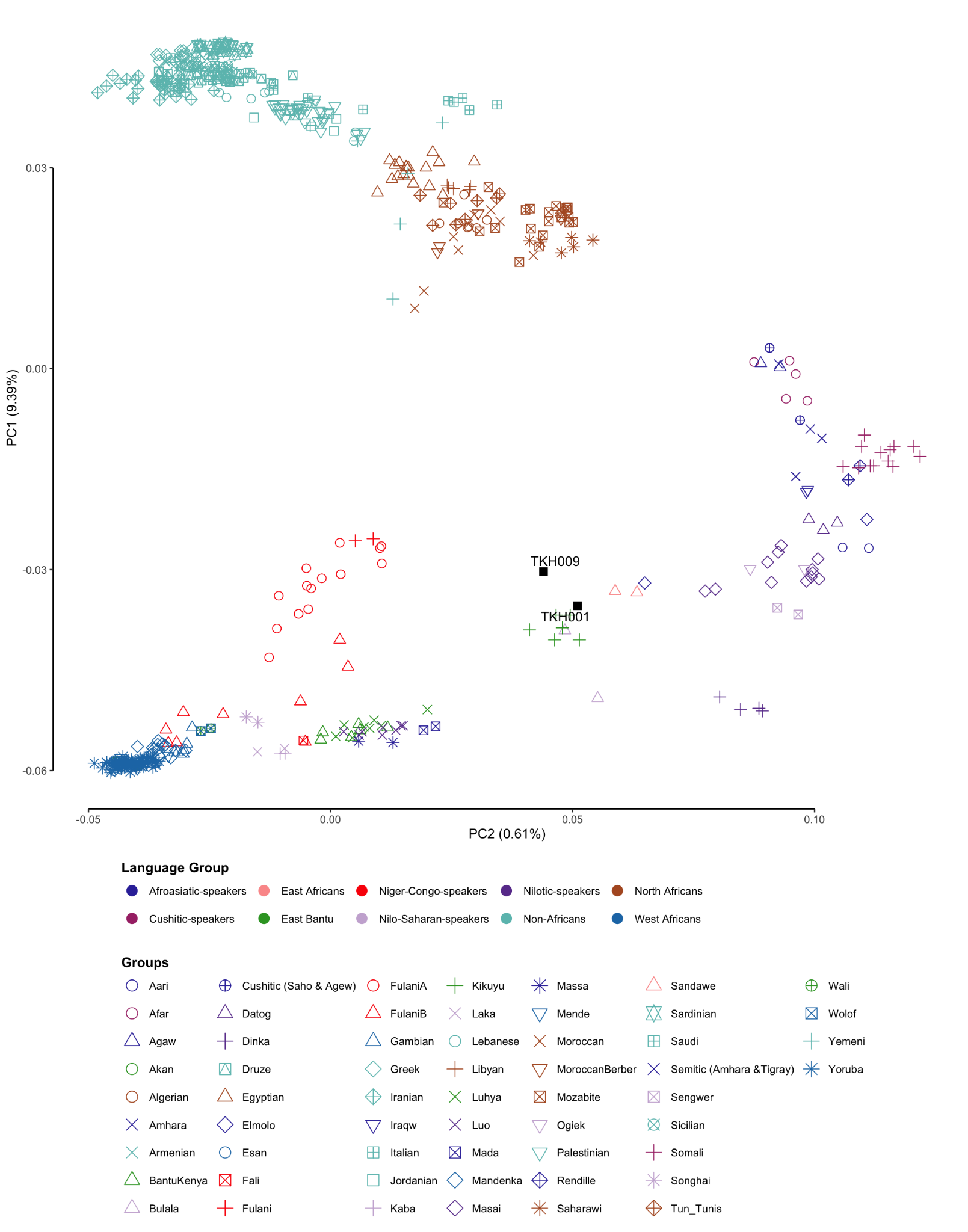


**Supplementary Figure 2.10: A)** PCA constructed on present-day populations in Africa, the Near East, and Southern Europe based on the Reduced SNP dataset including the individuals from Fortes-Lima et al. 2022[^47^](https://paperpile.com/c/AGF0JC/MuoE). Takarkori individuals projected and highlighted in black squares. **B)** Geographic locations of Takarkori individuals and relevant present-day populations included in our analysis.
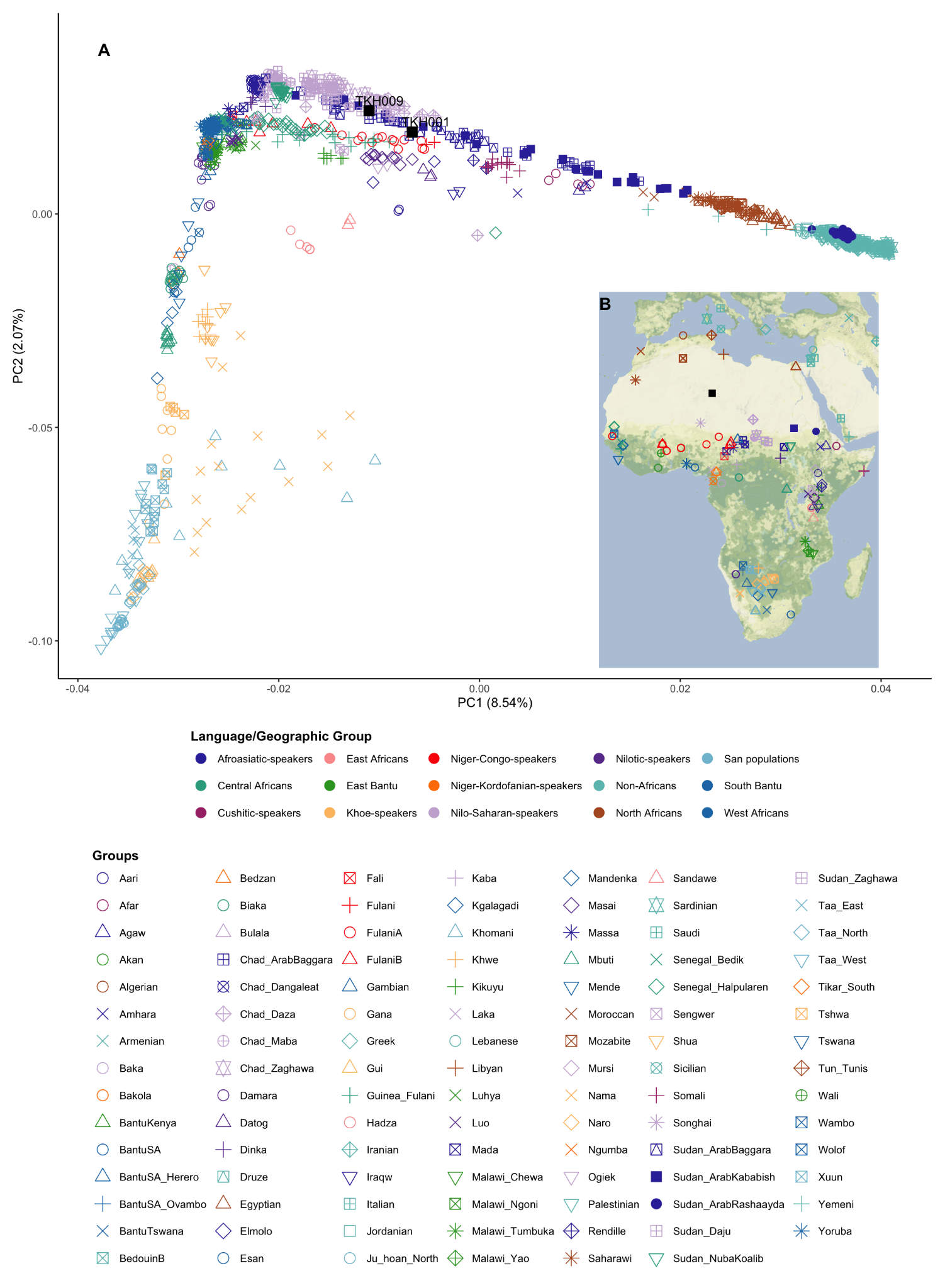


**Supplementary Figure 2.11:** PCA constructed on present-day populations in West Africa, the Sahel, and East Africa, as well as the Near East and Southern Europe, based on the 184K SNP dataset including the individuals from Fortes-Lima et al. 2022[^47^](https://paperpile.com/c/AGF0JC/MuoE). Takarkori individuals are projected and highlighted in black squares.
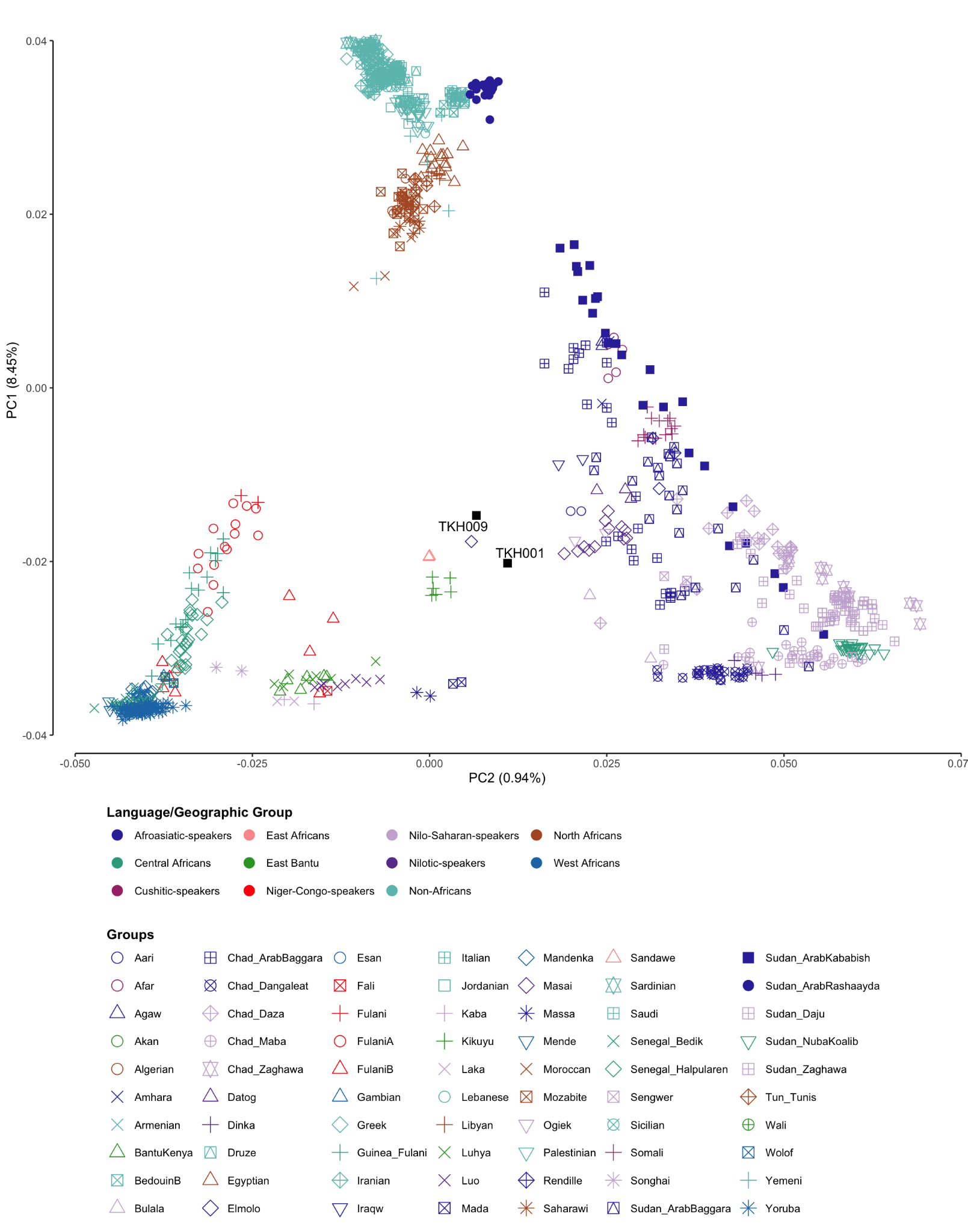


### *F*-statistics

All *f*_3_- and *f*_4_-statistics were calculated with ADMIXTOOLS[^48^](https://paperpile.com/c/AGF0JC/JjjJ) package v.5.0 (<https://github.com/DReichLab/AdmixTools> ) using *qp3pop* v.420 and *qpDstat* v.721, respectively.

#### **Outgroup-*f*_3_**

##### **Affinity with ancient populations**

Unless stated otherwise, the following analyses are based on the 1240k dataset. The Outgroup-*f*_3_ test, as depicted in Fig. 3A, was conducted using the genomes of South Africa 2000 calBP as an outgroup, with the inbreed option enabled for qp3pop. This analysis was intended to identify populations that share genetic drift with Takarkori. The top 70 outgroup-*f*_3_ signals between Takarkori and ancient populations worldwide are shown in **Supp. Fig. 2.12**.

Additionally, we conducted the same test but replaced Takarkori with Taforalt to identify populations that share genetic drift with Taforalt. The top 70 outgroup-*f*_3_ signals between Taforalt and ancient populations are presented in **Supp. Fig. 2.13**. In the first outgroup-*f*_3_ test (South Africa 2000 calBP; X, Takarkori), Taforalt and the related later Epipaleolithic and Neolithic Moroccan groups show the highest values. In contrast, Takarkori displays an intermediate *f3* signal towards Taforalt and these Moroccan groups in the second outgroup-*f*_3_(South Africa 2000 calBP; X, Taforalt) **(Supp. Fig. 2.13)**, immediately following the Eurasian groups. This finding is expected, as Taforalt carries a majority amount (around 60%) of Eurasian ancestry, leading to a higher affinity with Eurasian-admixed groups.

The full pairwise clustering matrix displayed in **Extended Data Fig. 3** is based on the outgroup-*f*_3_ test (Chimpanzee, X1, X2), where X represents ancient populations from Africa and the Near East. Here, we utilized the Chimpanzee because it serves as a valid outgroup for all tested populations. The *qp3pop* program was executed with the 'outgroupmode' option activated. This setting is used to establish a flat denominator of 0.01. This is necessary because the built-in heterozygosity normalization in *qp3pop* fails when the outgroup is a haploid genome, as is the case with the Chimpanzee. The signals produced by this test are qualitatively similar to the above-mentioned outgroup-*f_3_* test (South Africa 2000 calBP; X, Takarkori).

Next, we sought to quantitatively explore whether Takarkori could be part of a broader ancient genetic gradient across Africa or if it is a uniquely distinct group. To do this, we created a scatter plot showing the correlation between the geographic distance from Takarkori and the *f3* values for published ancient African populations. We obtained a negative correlation (correlation coefficient: -0.617), suggesting a gradient in genetic similarity to Takarkori that decreases with increasing distance **(Supp. Fig. 2.14)**. However, this scatter plot should be interpreted with caution, as Levantine-related ancestry may be driving this correlation. For example, the Cameroon Shum Laka individuals (Ancient Central African foragers) are at an equal distance from Takarkori as the Ancient North African populations, yet they share the same *f3* values as the Ancient East African foragers, despite being 1,000 km further away.

When rerunning the correlation analysis with ancient Eurasian-admixed groups from Morocco, Egypt and East Africa removed (**Supp. Fig. 2.15**), we indeed do not observe any significant correlation - suggesting that Tarkakori is not part of a genetic continuum. This pattern is also apparent in present-day genomes, which reveal that the Sahara forms an area of genetic discontinuity[^49^](https://paperpile.com/c/AGF0JC/iJ7E).

**
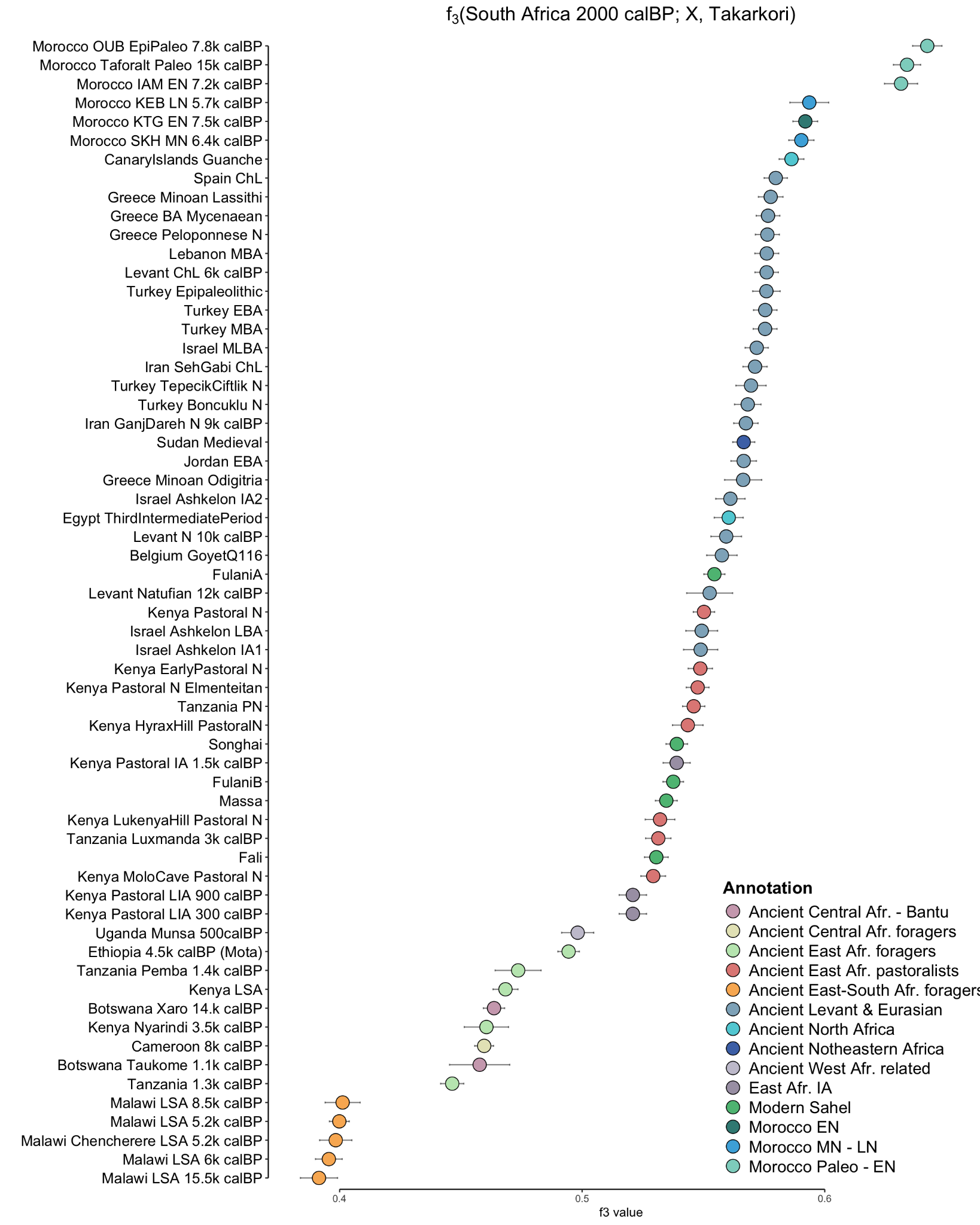
Supplementary Figure 2.12:** The top 70 outgroup-*f*_3_ signals between Takarkori and various ancient populations, using the South African 2000 calBP genome as an outgroup. The horizontal bars represent ± 3 SE estimated by 5 cM block jackknifing.

**
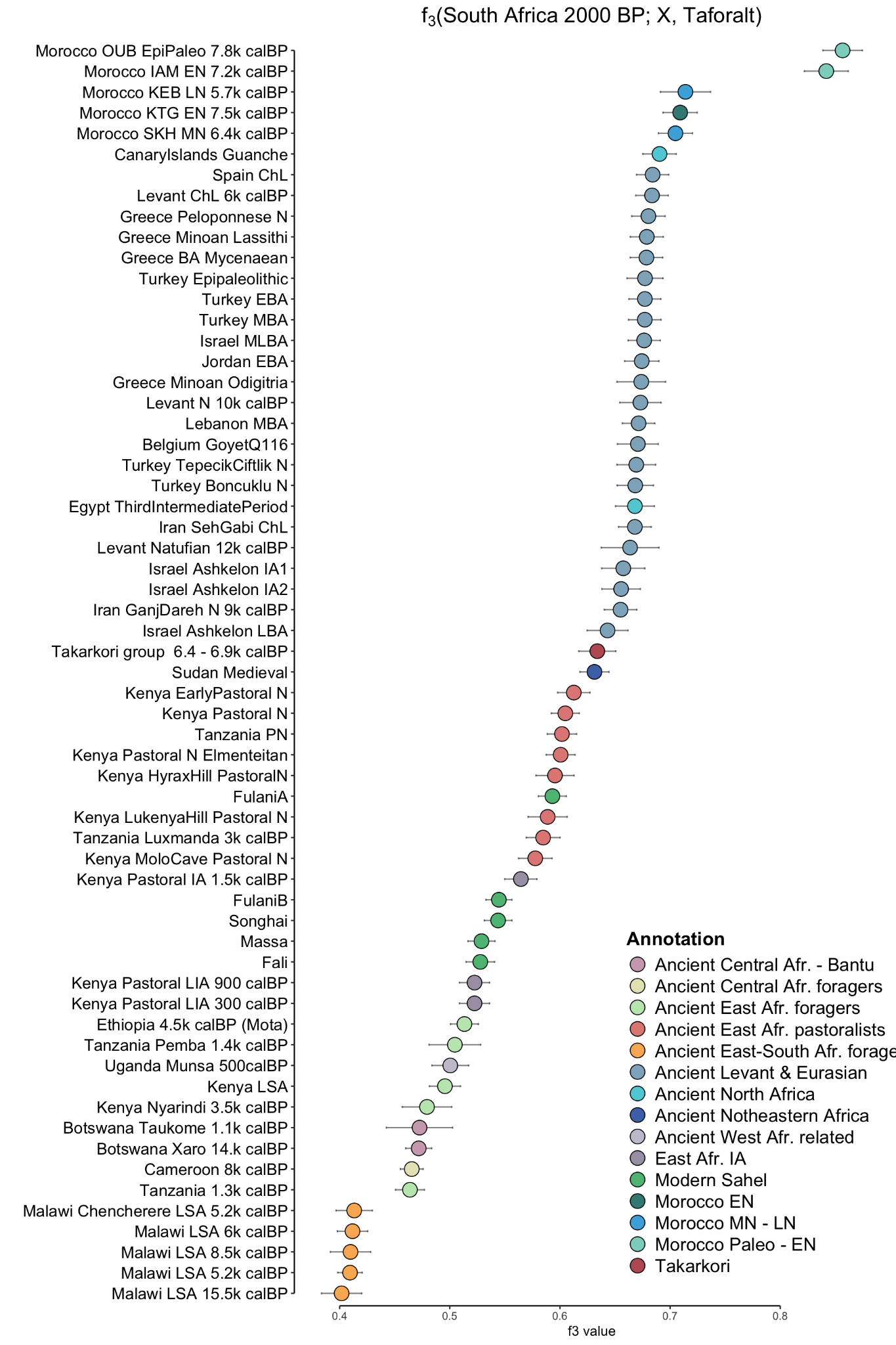
**

**Supplementary Figure 2.13:** The top 70 outgroup-*f*_3_ signals between Taforalt and various ancient populations, using the South African 2000 calBP genome as an outgroup. The horizontal bars represent ± 3 SE estimated by 5 cM block jackknifing.

**
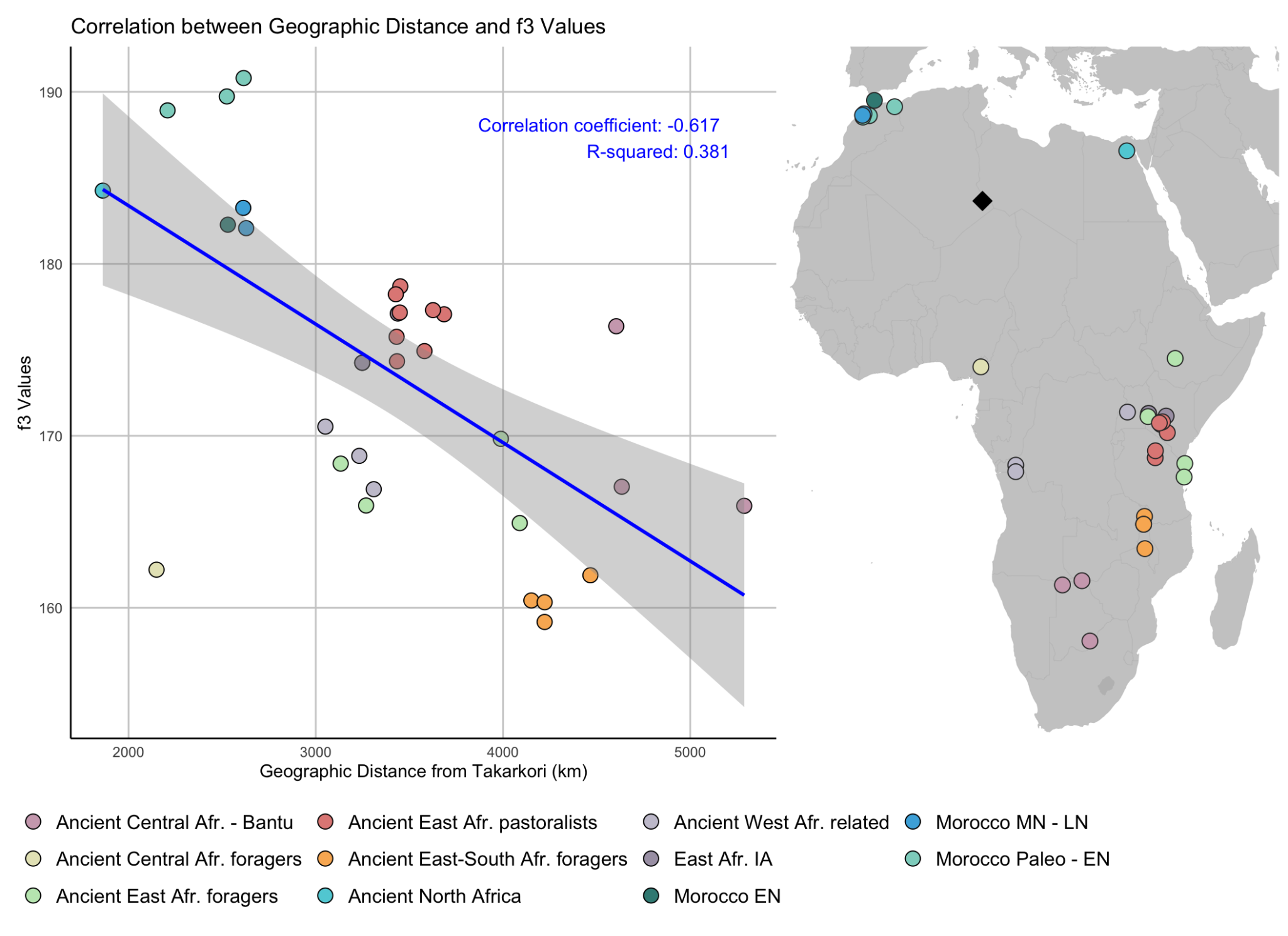
Supplementary Figure 2.14:** Scatter plot showing the relationship between geographic distance from Takarkori and *f3* values for ancient African populations. The plot indicates a negative correlation (correlation coefficient: -0.617), with a gradient in genetic similarity decreasing with geographic distance. Grey area represents the 95% confidence interval for the regression line.

**
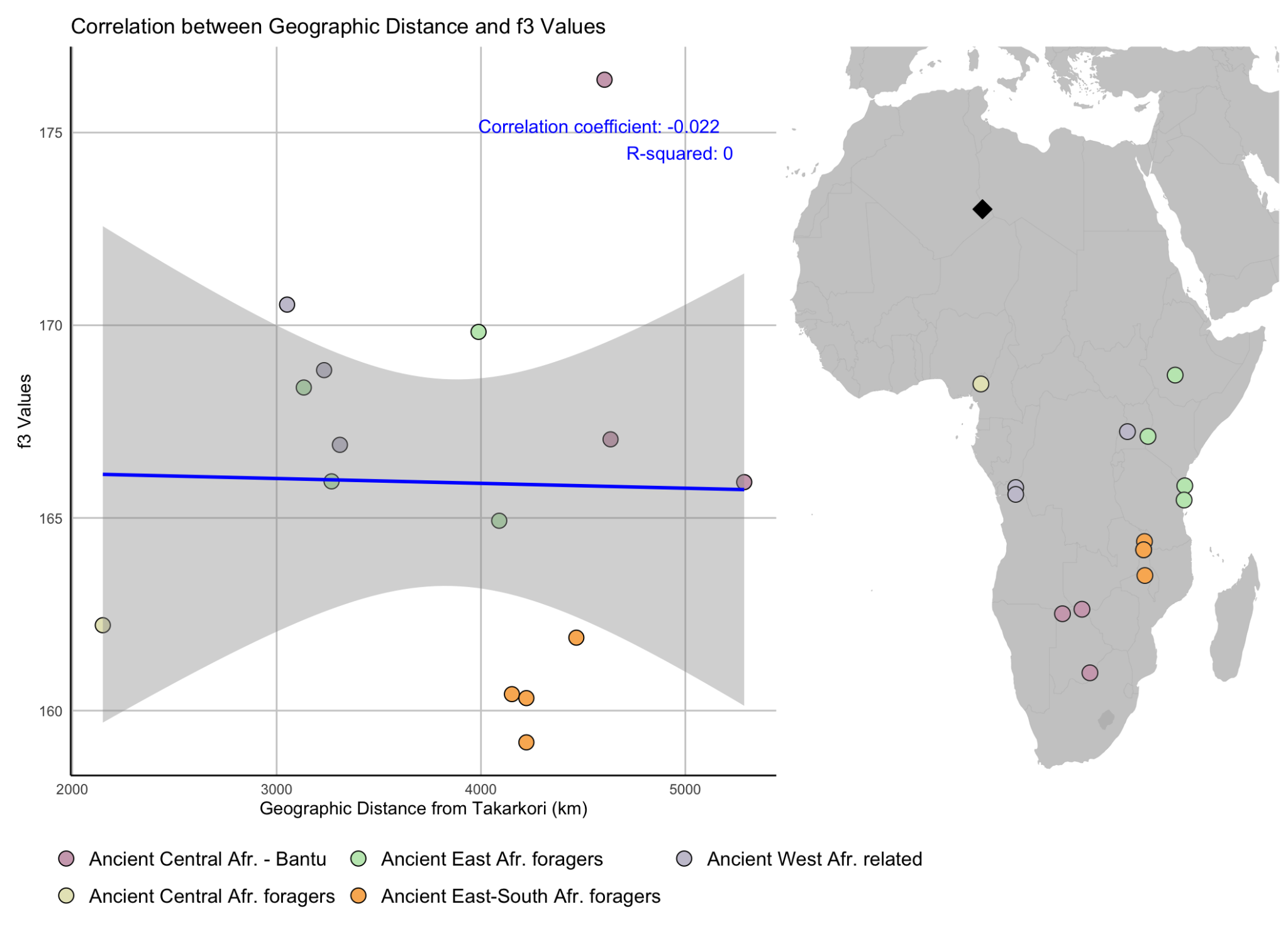
Supplementary Figure 2.15:** Scatter plot showing the relationship between geographic distance from Takarkori and *f3* values for ancient African populations without Levantine-related ancestry. The initial negative correlation (correlation coefficient: -0.617, see **Supp. Fig. 2.14**) drops to -0.022 when excluding groups with Levantine-related ancestry, indicating that geographic distance alone does not explain the genetic similarities. Grey area represents the 95% confidence interval for the regression line.

##### **Affinity with present-day populations**

Next, we investigated the ancestral legacy of Takarkori in present-day populations by applying the outgroup-*f*_3_ test (Chimpanzee; Present-day groups, Takarkori), focusing on populations from Africa and the Near East (**Extended Data Fig. 4**). Our findings indicate that some Sahelian Fulani groups (designated as FulaniA) show an increased affinity to Takarkori, although their affinity to Taforalt is stronger. This finding aligns with D’Atanasio et al.[^44^](https://paperpile.com/c/AGF0JC/4zFs), who identified a non-sub-Saharan component in the Fulani group similar to that found in the Taforalt and later Neolithic groups. Other Sahelian groups (FulaniB, Songhai, Massa, Fali, Wolof) as well as North African and Levantine groups, exhibit overlapping *f*_3_ signals, suggesting a nearly equal amount of shared genetic drift with Takarkori. The results are displayed in **Supp. Fig. 2.16.**

We performed Pairwise Outgroup-*f3* (Chimp; x, x) analyses using both the Reduced SNP dataset (**Supp. Fig. 2.17**) and the 184K SNP Dataset (**Supp. Fig. 2.18**). In both cases, we did not observe distinguished *f3* signals between Takarkori and present-day groups, including Moroccan and Tunisian individuals from Lucas-Sánchez et al. 2023[^46^](https://paperpile.com/c/AGF0JC/6Loh), or those from the Sahel/Savannah belt described in Fortes-Lima et al. 2022[^47^](https://paperpile.com/c/AGF0JC/MuoE). Additionally, the FulaniA group did not retain the higher *f*3 signal with Takarkori seen with the Comprehensive SNP dataset, suggesting that the reduced SNP coverage in these datasets may be less effective in capturing genetic drift.

**Supplementary Figure 2.16: Shared genetic drift with Takarkori genomes.** **A)** Outgroup-$f$_3_ statistics of the form $f$_3_(Takarkori, X; Chimpanzee), where X represents modern groups, mapped at their geographical positions in circle shapes, with ancient Taforalt represented by diamonds, and the Sahelian Fulani group by triangles. The gradient from blue to green indicates genetic proximity to Takarkori, with blueish colors representing closer genetic relationships. **B)** The statistics and their associated three SEs for the top 40 signals are presented.
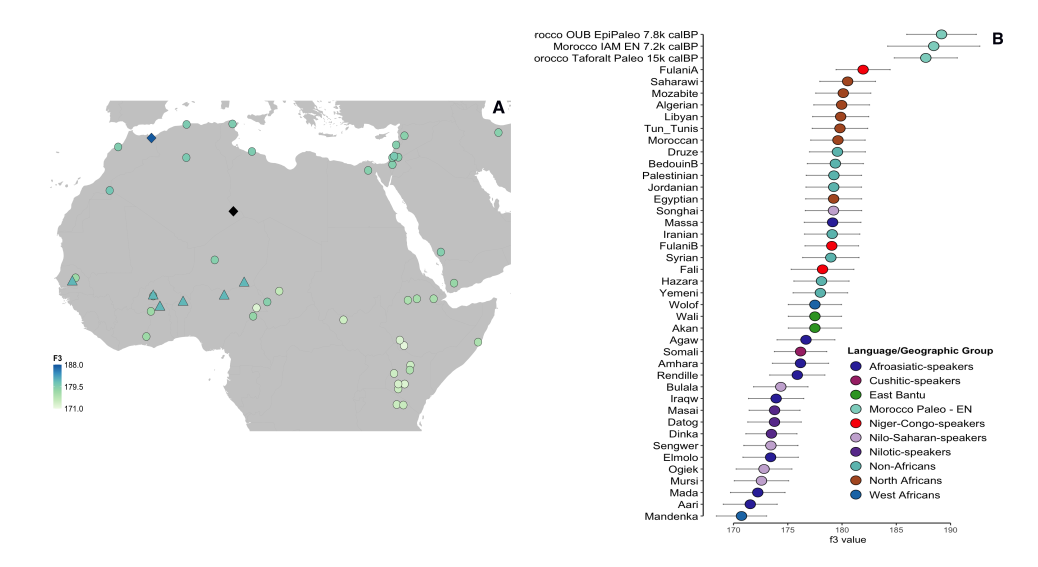


**Supplementary Figure 2.17**: Pairwise clustering matrix derived from Outgroup-$f$_3_(Chimp; X1, X2) statistics, where X represents relevant ancient populations from Africa and the Near East, based on the Reduced SNP dataset. The Takarkori group does not exhibit distinct shared genetic drift with any present-day groups.
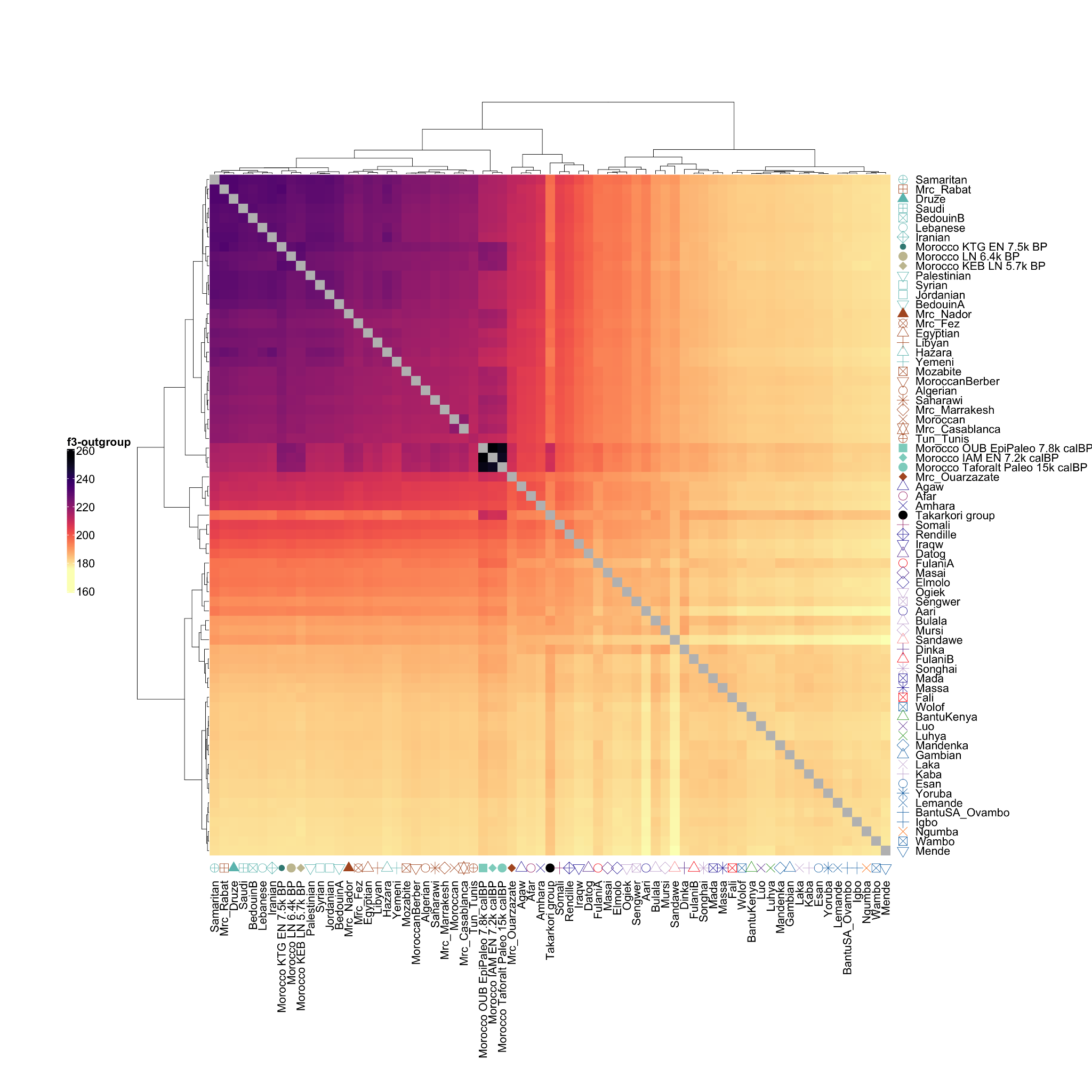


**Supplementary Figure 2.18:** Pairwise clustering matrix derived from Outgroup-$f$_3_(Chimp; X1, X2) statistics, where X represents relevant ancient populations from Africa and the Near East, based on the 184K SNP dataset. The Takarkori group does not exhibit distinct shared genetic drift with any present-day groups.
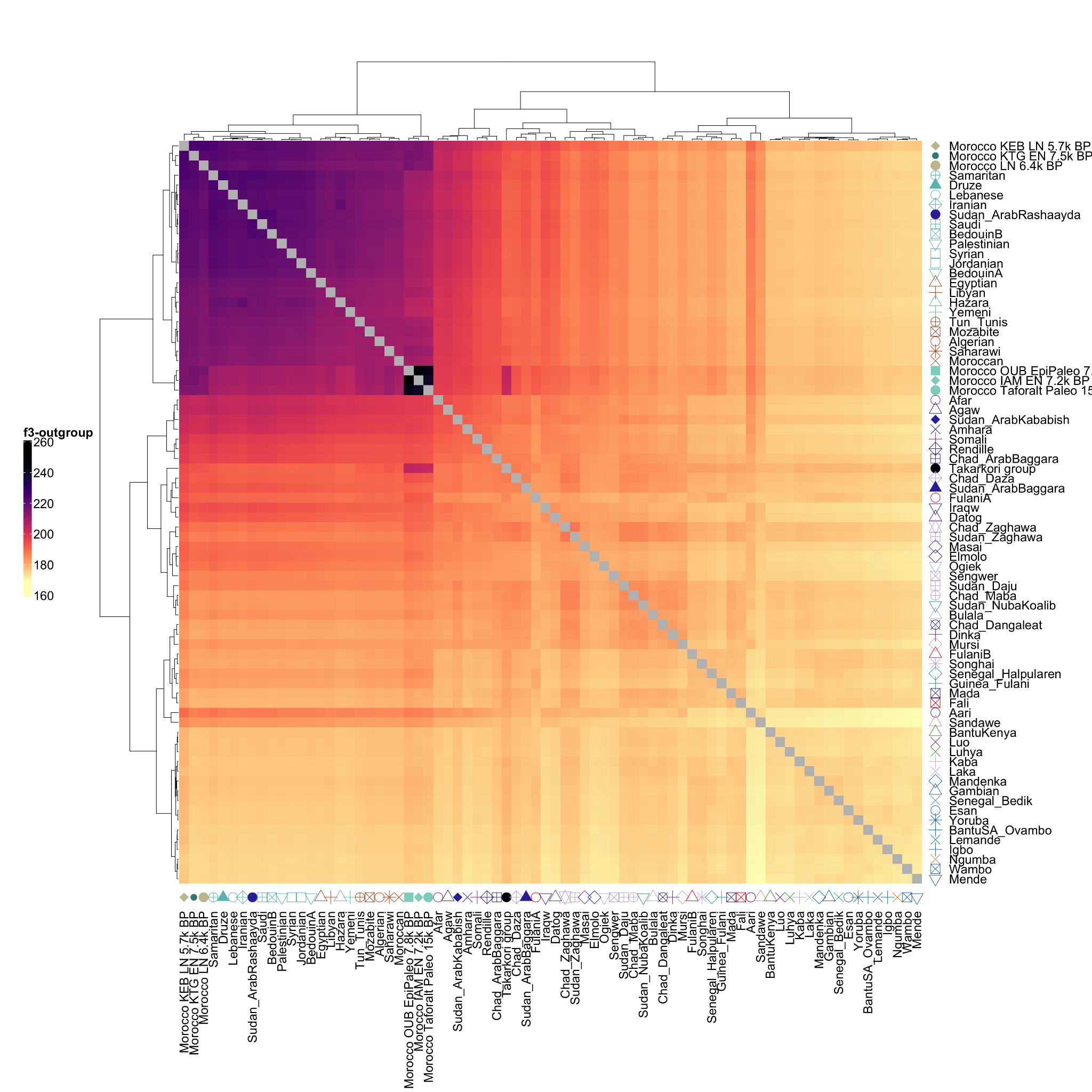


#### ***f_4_-*statistics**

Given the strong genetic drift shared between Takarkori and Taforalt in the outgroup *f*_3_-statistics, we further examined whether Takarkori genomes share more alleles with other human groups compared to Taforalt. Using the Comprehensive HO dataset, we calculated the *f*_4_(Chimpanzee, X; Takarkori, Taforalt) statistic, where X represents ancient and present-day populations from Africa and Eurasia (**Supp. Fig. 2.19, Supp. Data 4**). The results showed significantly positive values for Eurasian and Eurasian-admixed African groups, indicating that Taforalt shares more alleles with these groups than Takarkori. In contrast, all sub-Saharan African groups are equally related between Takarkori and Taforalt.

Previously, we showed that in the Outgroup-*f_3_*(Chimp; X1, X2) statistics, where X represents relevant modern populations from Africa and the Near East, Sahelian Fulani groups (designated as FulaniA) exhibit increased affinity to Takarkori, though to a lesser extent than their affinity to Taforalt (**Extended Data Fig. 4, Supp. Fig. 2.13**). To further confirm Takarkori-like ancestry in the Fulani, we conducted an *f_4_* analysis, *f_4_*(Chimpanzee, X; Masai/Datog/Iraqw, Taforalt), using Masai/Datog/Iraqw as baseline references due to their similar amount of OoA ancestry as Takarkori (**Supp. Fig. 2.20.1 - 2.20.3**). The results indicated that the Fulani have Takarkori-like ancestry, indistinguishable from other Sahelian groups.

**
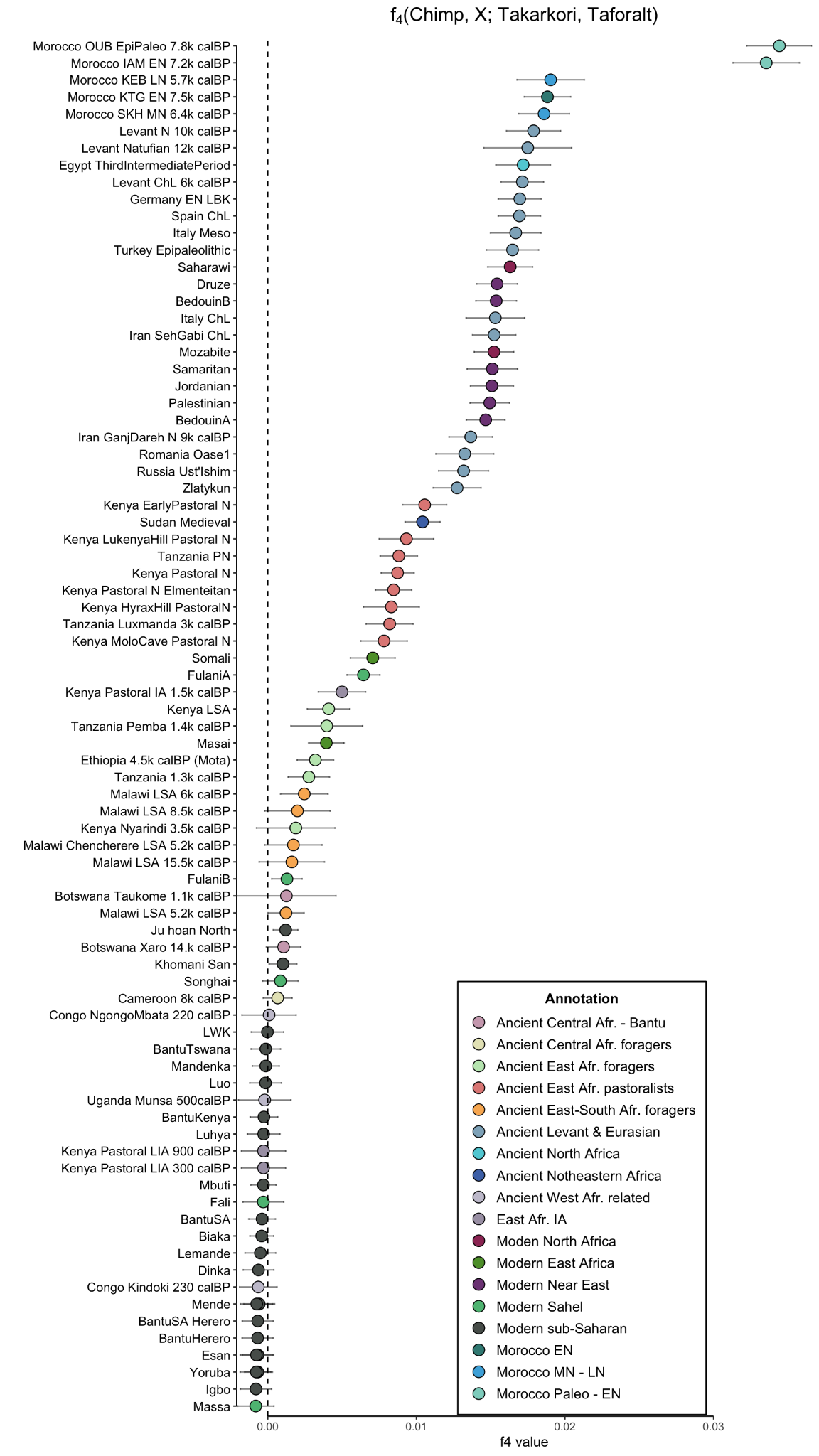
**

**Supplementary Figure 2.19:** No group shares significant extra affinity with Takarkori genomes in comparison to Taforalt, as measured by *f_4-_s*tatistics in the from $f$*_4_*(Chimpanzee, X; Takarkori, Taforalt). The error bars represent ± 3 SE estimated by 5 cM block jackknifing.

**Supplementary Figure 2.20.1:** *f_4_*(Chimp, X; Masai, Takarkori) test used to detect Takarkori-like ancestry across present-day populations from Africa, while controlling for shared OoA drift. Positive f4 values indicate populations with greater genetic affinity to Takarkori. Populations are color-coded by language or geographic group. The error bars represent ± 3 SE estimated by 5 cM block jackknifing.
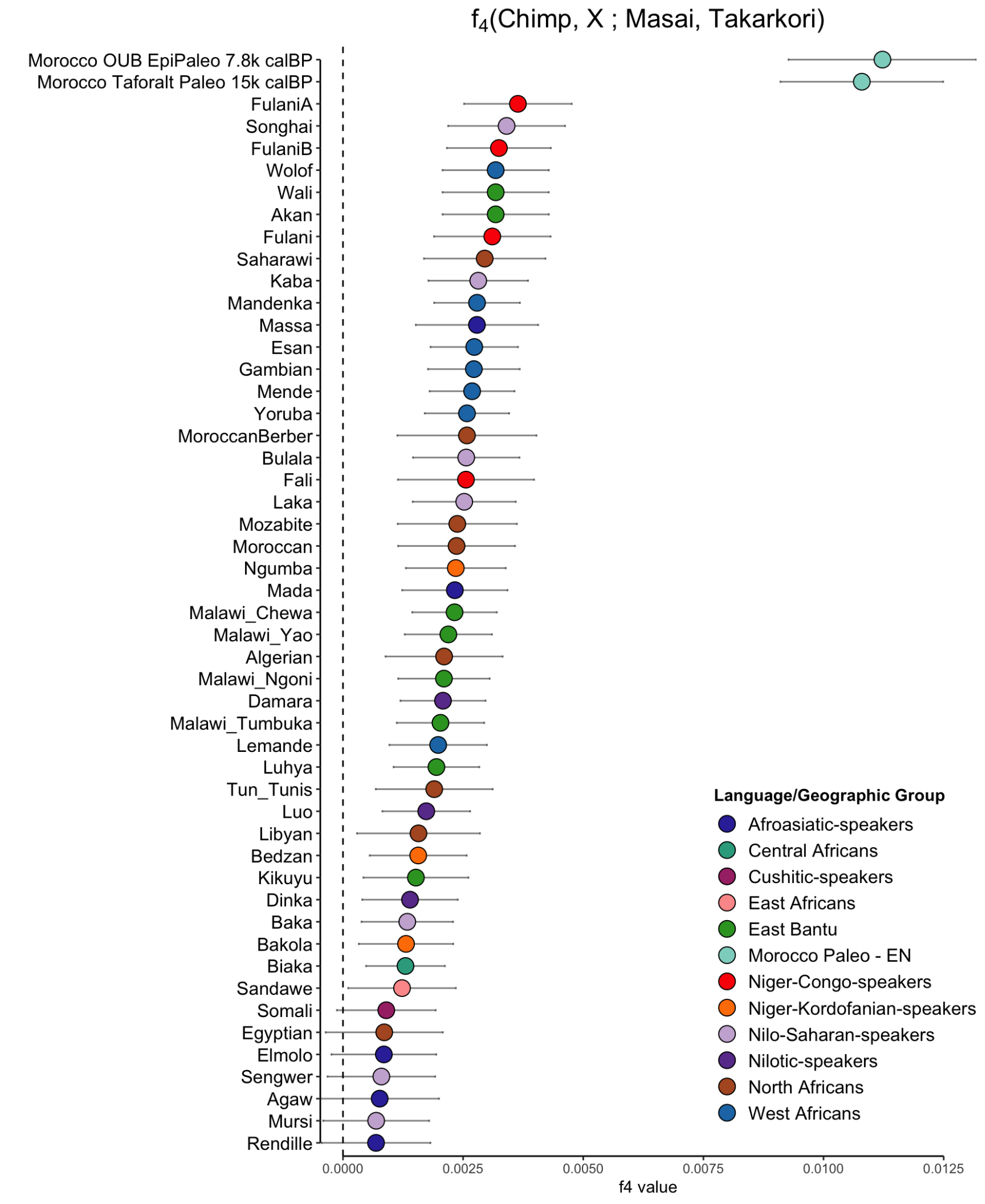


**Supplementary Figure 2.20.2:** *f_4_*(Chimp, X; Iraqw, Takarkori) test used to detect Takarkori-like ancestry across present-day populations from Africa, while controlling for shared OoA drift. Positive f4 values indicate populations with greater genetic affinity to Takarkori. Populations are color-coded by language or geographic group. The error bars represent ± 3 SE estimated by 5 cM block jackknifing.
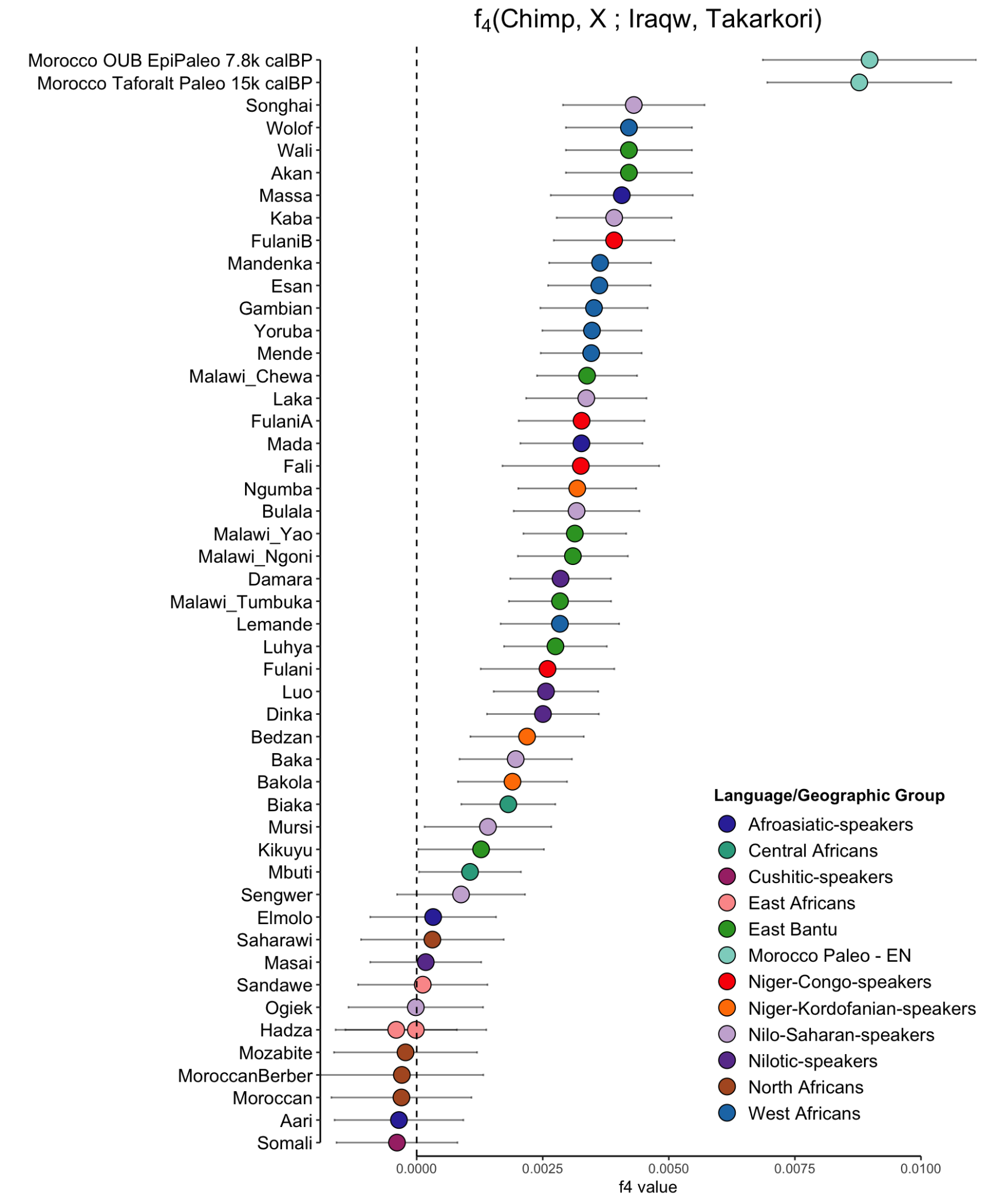


**Supplementary Figure 2.20.3:** *f_4_*(Chimp, X; Datog, Takarkori) test used to detect Takarkori-like ancestry across present-day populations from Africa, while controlling for shared OoA drift. Positive f4 values indicate populations with greater genetic affinity to Takarkori. Populations are color-coded by language or geographic group. The error bars represent ± 3 SE estimated by 5 cM block jackknifing.
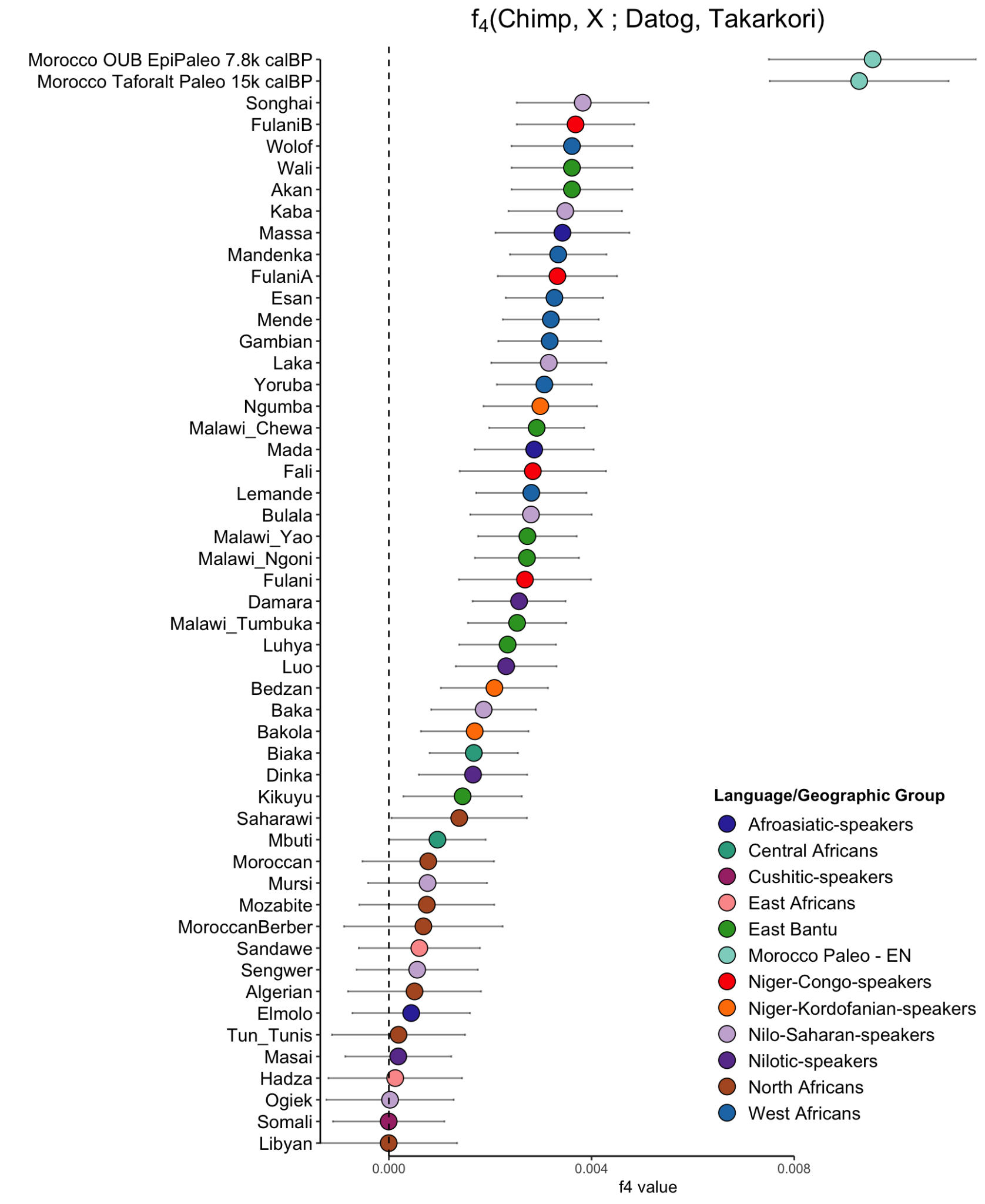


#### ***f*_4_-statistics** **based on SNPs ascertained in African populations**

Ascertainment bias results from the process used to select SNPs for microarray chips. By design, these chips only analyze sites that are known to be variable in at least one population. The Affymetrix Human Origins 1 Array[^48^](https://paperpile.com/c/AGF0JC/JjjJ) that we utilized in our study—part of the 1240k panel—comprises 13 distinct SNP sets. Each of these sets was identified as heterozygous based on a single genome sequence from one of 11 different populations (**Supp. Table 2.5**). Conducting separate analyses of the data from these different SNP panels can provide valuable insights into potential population genetic biases[^50^](https://paperpile.com/c/AGF0JC/53Lk). In this study, we applied this strategy to test if the Takarkori and Taforalt populations have additional sub-Saharan ancestry. This was accomplished by performing *f_4_*-statistics using Mbuti and Yoruba ascertained SNPs, separately. The results are illustrated in **Supp. Fig. 2.21** and **Supp. Fig. 2.22**. In both analyses, we did not observe any significant sub-Saharan affinity in either the Takarkori or Taforalt populations.

|  |
| --- |

**Supplementary Table 2.5**: Summary of SNP count within populations targeted by the Affymetrix Human Origins 1 Array design.

| **Panel no.** | **Population** | **Sample ID** | **No. SNPs per population** |
| --- | --- | --- | --- |
| 1 | French | HGDP00521 | 111,970 |
| 2 | Han Chinese | HGDP00778 | 78,253 |
| 3 | Papuan1 | HGDP00542 | 48,531 |
| 4 | San | HGDP01029 | 163,313 |
| 5 | Yoruba | HGDP00927 | 124,115 |
| 6 | Mbuti Pygmies | HGDP00456 | 12,162 |
| 7 | Karitiana | HGDP00998 | 2,635 |
| 8 | Sardinian | HGDP00665 | 12,922 |
| 9 | Melanesian | HGDP00491 | 14,988 |
| 10 | Cambodian | HGDP00711 | 16,987 |
| 11 | Mongolian | HGDP01224 | 10,757 |
| 12 | Papuan2 | HGDP00551 | 12,117 |
| 13 | Denisova-San | Den-HGDP01029 | 151,435 |

**Supplementary Figure 2.21:** No significant excess sub-Saharan affinity is observed in the genomes of Takarkori or Taforalt, as measured by f_4_-statistics in the form $f$_4_(Chimpanzee, X; Takarkori, Taforalt), using the Mbuti ascertained SNPs set. The error bars represent ± 3 SE estimated by 5 cM block jackknifing.
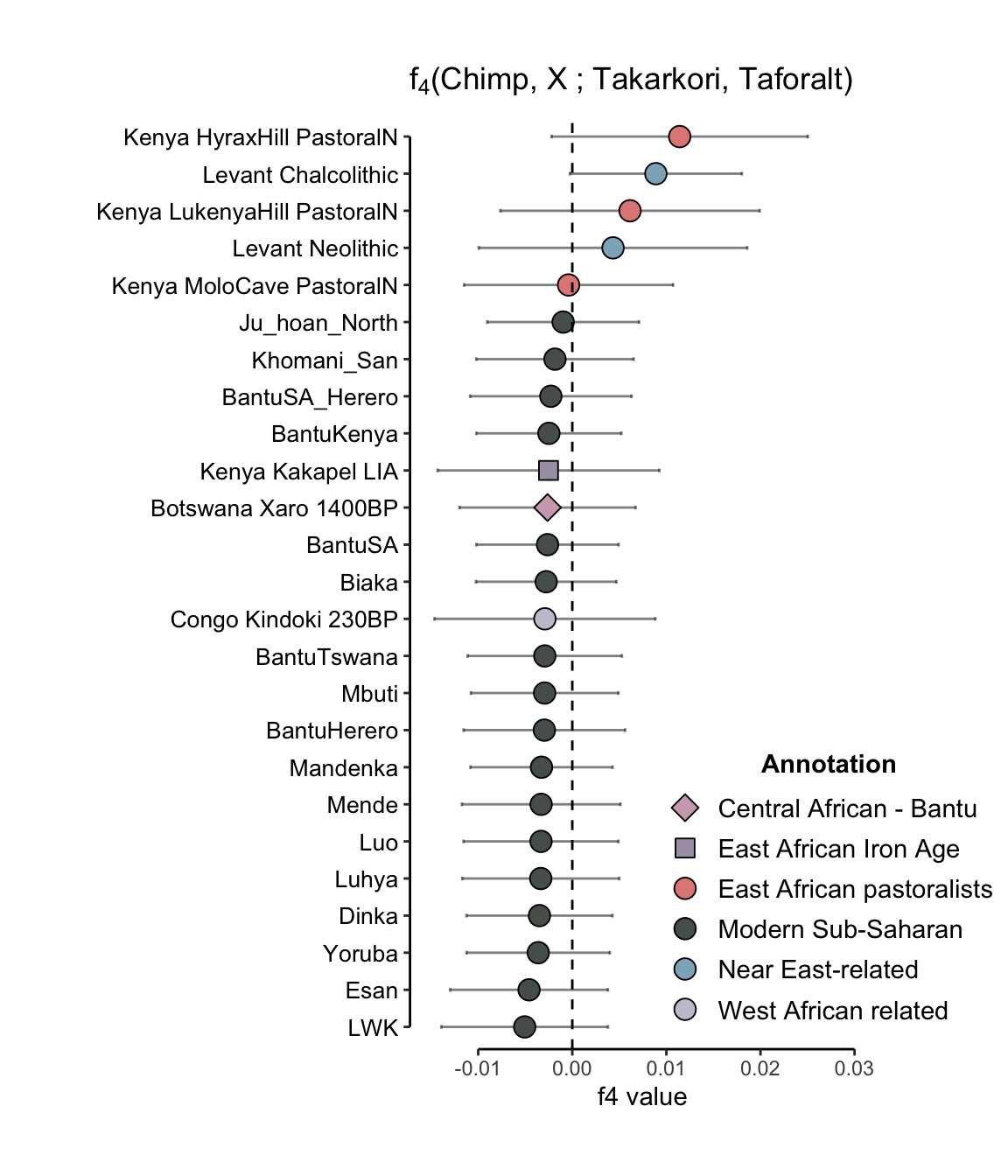


###
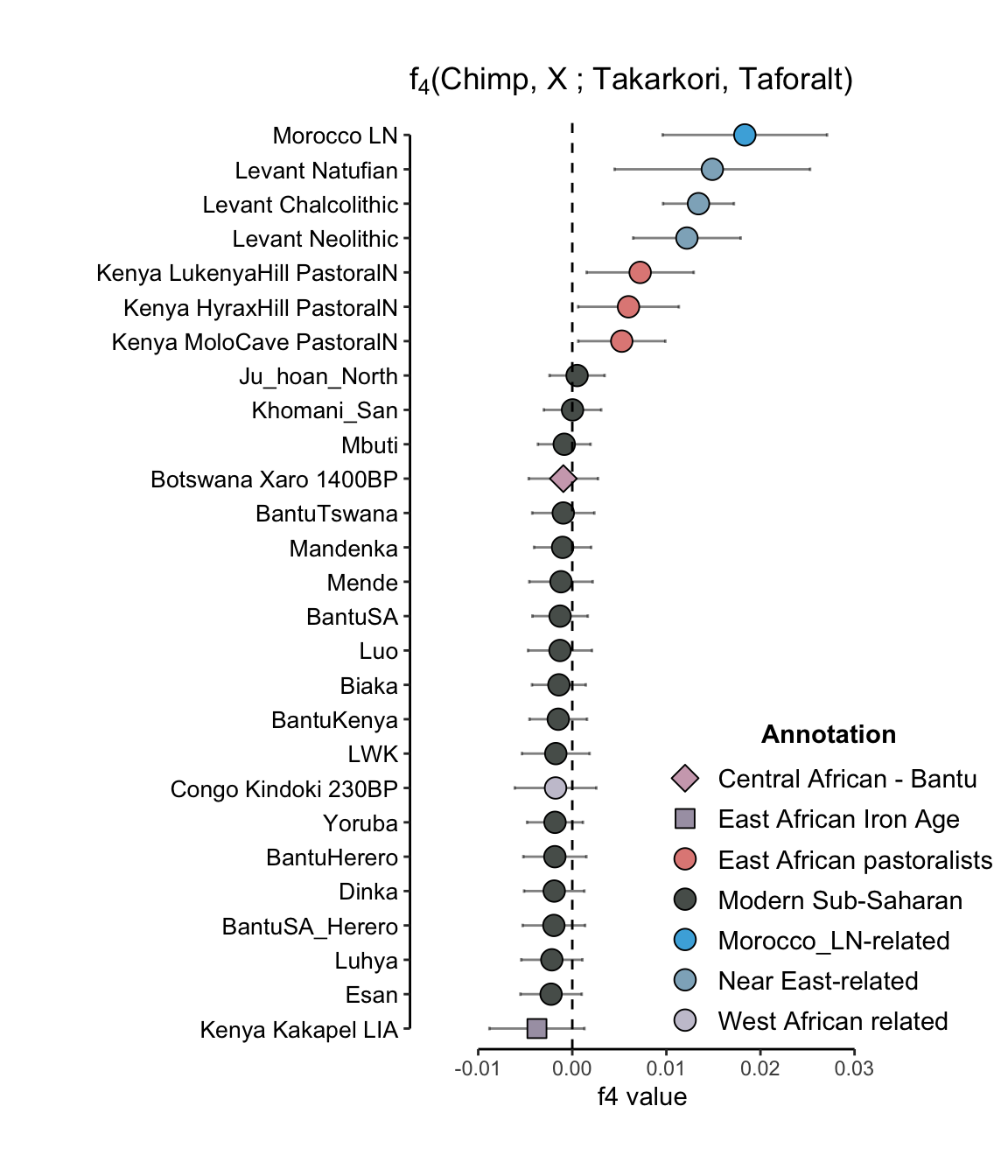


**Supplementary Figure 2.22:** No significant excess sub-Saharan affinity is observed in the genomes of Takarkori or Taforalt, as measured by f_4_-statistics in the form $f$*_4_*(Chimpanzee, X; Takarkori, Taforalt), using the Yoruba ascertained SNPs set. The error bars represent ± 3 SE estimated by 5 cM block jackknifing.

### qpAdm modeling for Taforalt

To model the Taforalt group as a two-way admixture between Natufian and various sources of African ancestry, we utilized the *qpAdm*[*^51^*](https://paperpile.com/c/AGF0JC/VxTvs) (v810) implemented in *AdmixTools* package v.5.1 (<https://github.com/DReichLab/AdmixTools>). For each run, we created a fixed outgroup set that included Onge, Han, Papuan, Ust’-Ishim, Kostenki14, MA-1, and Iran_N. Alongside these, we also included all the African ancestry sources with the exception of the one being tested as the source in that specific run. This rotating scheme, with a different African source population in each run, allowed us to test multiple admixture scenarios for the Taforalt group.

In our analysis, the rotating scheme revealed that Takarkori best fits the model as an African source for the two-way admixture, attaining a P-value > 0.05, indicative of a good model fit. Conversely, all the other tested sub-Saharan African populations—Yoruba, Dinka, Mota, Cameroon_ShumLaka_hc, Botswana_Xaro_EIA, and Tanzania_Zanzibar_1300calBP—did not fit well in the model as their P-values <10e-34. Results are detailed in **Supp. Table 2.6**.

**Supplementary Table 2.6**: Results of the qpAdm analysis modeling Taforalt group as a two-way admixture between Natufian and various African populations. Takarkori yielded the best model fit with a P-value > 0.05. Coefficients for African and Natufian admixture (CoefAFR and CoefNat), along with their standard errors (SE), are reported.

| **African source** | **P-value** | **Coef_Nat_** | **Coef_AFR_** | **SE** |
| --- | --- | --- | --- | --- |
| Takarkori | 0.229 | 0.608 | 0.392 | 0.018 |
| Yoruba | 1.37x10^-35^ | 0.739 | 0.261 | 0.017 |
| Dinka | 2.84x10^-34^ | 0.706 | 0.294 | 0.018 |
| Mota | 5.92x10^-42^ | 0.608 | 0.304 | 0.020 |
| Cameroon ShumLaka | 1.99x10^-40^ | 0.741 | 0.259 | 0.016 |
| Botswana Xaro EIA | 3.32x10^-40^ | 0.737 | 0.263 | 0.016 |
| Tanzania Zanzibar 1.3k calBP | 2.21x10^-43^ | 0.718 | 0.282 | 0.019 |

###

To rule out that the inclusion of Takarkori in the reference set disrupts the other models due to shared genetic affinity of Takarkori with Taforalt, we re-ran the above-mentioned models using the same reference set but excluded Takarkori as an outgroup in our rotating scheme. However, we still could not find a model with a P-value greater than 0.05. The models using Yoruba and Dinka resulted in borderline P-values of approximately 0.011, which is more than an order of magnitude less than for Takarkori as source (p=0.220) We list our results in **Supp. Table 2.7**.

**Supplementary Table 2.7**: Results of the qpAdm analysis modeling the Taforalt group as a two-way admixture between Natufian and various African populations, using a reference set that excludes Takarkori. No African source yielded a model fit with a P-value > 0.05. Coefficients for African and Natufian admixture (CoefAFR and CoefNat), along with their standard errors (SE), are reported.

| **African source** | **P-value** | **Coef_Nat_** | **Coef_AFR_** | **SE** |
| --- | --- | --- | --- | --- |
| Yoruba | 0.011 | 0.704 | 0.296 | 0.013 |
| Dinka | 0.011 | 0.670 | 0.330 | 0.013 |
| Mota | 7.18x10^-06^ | 0.643 | 0.357 | 0.015 |
| Cameroon ShumLaka | 0.009 | 0.684 | 0.316 | 0.013 |
| Botswana Xaro EIA | 9.37x10^-05^ | 0.686 | 0.314 | 0.013 |
| Tanzania Zanzibar 1.3k calBP | 5.40x10^-11^ | 0.676 | 0.324 | 0.015 |

###

### Admixture graph analysis

To investigate the ancestral relationship of Takarkori with other populations, we employed the `find_graphs()` function from ADMIXTOOLS2[^52^](https://paperpile.com/c/AGF0JC/4RDtT). This function is designed to identify admixture graphs consistent with observed *f*-statistics. The process begins with a randomly generated admixture graph, which is then iteratively refined through a combination of random and targeted modifications. The goal is to minimize the residuals of *f*-statistics (max |$f$_4, expected_ – $f$_4, observed_|) to identify the graphs that best fit the data.

For our analysis, we designated the Chimpanzee as a valid outgroup applicable to all groups. Guided by the results from *f3-* and *f4-*statistics, we included specific representatives from key genetic clusters: the Morocco_Iberomaurusian group for North Africa, Ethiopia_4500BP.SG for sub-Saharan Africa, and both Israel_Natufian_published and Iran_Ganj_Dareh_Neolithic for Eurasia.

Since each find_graphs() run can generate different fitted graphs, even with fixed parameters, and may get stuck in local optima, we iterated the run 20 times to mitigate this limitation, with each run beginning with a different random graph. This approach enhances the likelihood of finding graphs close to the global optimum. Furthermore, we did not set any constraints during the runs, allowing for a comprehensive exploration of the data.

The set of graphs produced was filtered to include only those with an absolute Z-score < 3. In this filtering stage, prior knowledge from qpAdm results was considered, allowing both the Takarkori and Taforalt groups to be admixed. We finally obtained three models with comparable Z-scores, each exhibiting common characteristics (**Supp. Fig. 2.23 - 2.25**). Specifically, the divergence of the Mota, Out-of-Africa, and North African ghost populations occurred with very little intervening genetic drift, suggesting that the underlying splits occurred close in time. The North African ghost and Out-of-Africa populations independently branched off from Mota. Moreover, the Takarkori group contained between 73 and 93 percent of this North African ghost ancestry, substantially more than Morocco_Iberomaurusian.

The recent study by[^53^](https://paperpile.com/c/AGF0JC/wtT3) highlights that while SNP panels are effective for examining relationships among non-African populations and one African outgroup, their use in co-modeling multiple sub-Saharan African and archaic human groups (like Neanderthals and Denisovans) often leads to incorrect rejection of true demographic histories and acceptance of inaccurate models. However, we believe that this bias does not strongly apply in our study as we investigate mostly non sub-Saharan and non-archaic branches.


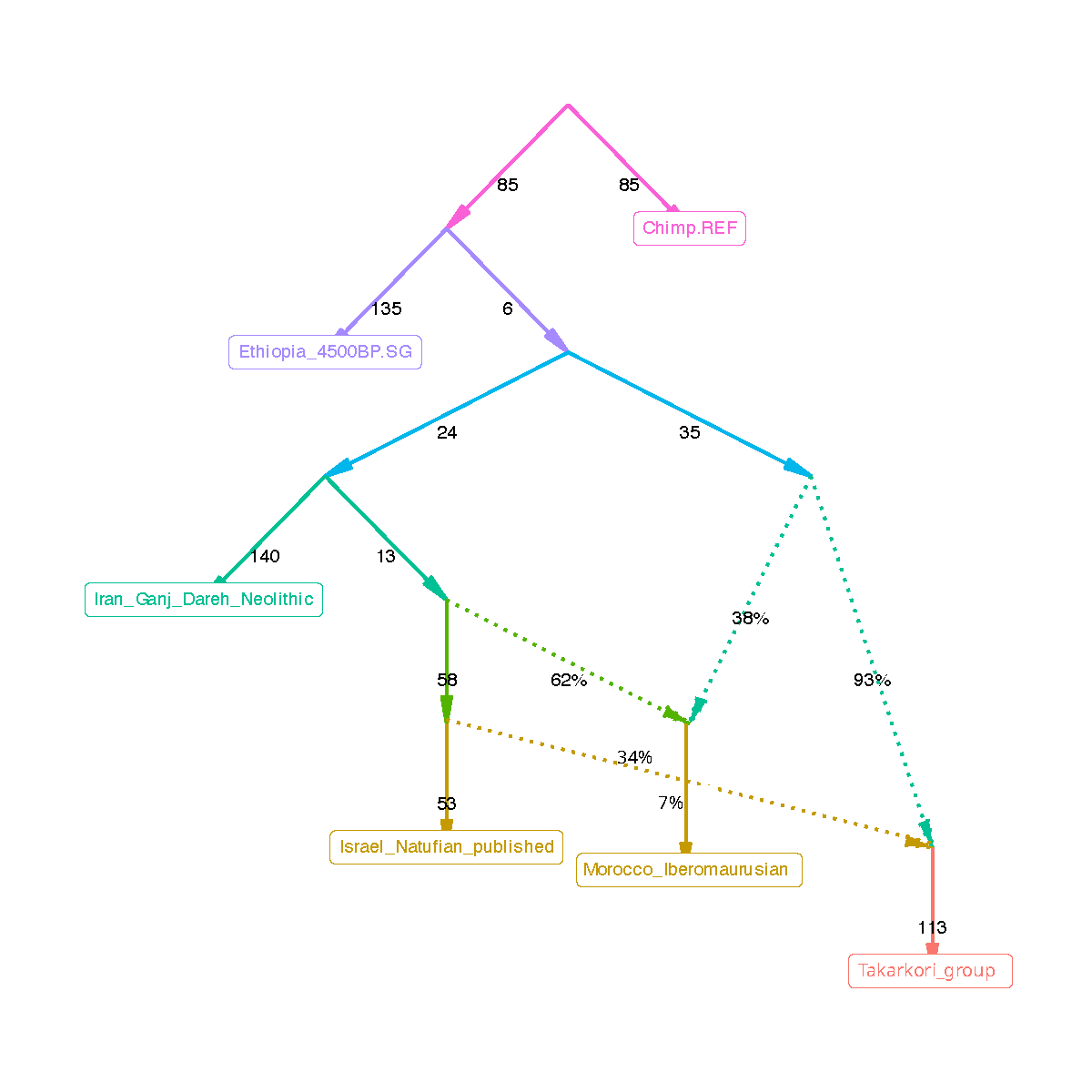


**Supplementary Figure 2.23:** Primary admixture graph modeling Takarkori's ancestral relationship with relevant populations. The model fits with *f*-statistics residuals Z-score (max |$f$_4, expected_ – $f$_4, observed_|) = 0.27 SE. This graph suggests that Takarkori traces most of its ancestry (93%) to a hitherto unknown North African population, and the remaining (7%) is derived from an ancient Levantine source (Natufian-like ancestry). The admixture graph matrix for this model is available in Supp. Data 6 (model 1).

**Supplementary Figure 2.24:** Alternative admixture graph modeling Takarkori's ancestral relationship with relevant populations. The model fits with f-statistics residuals Z-score (max |$f$_4, expected_ – $f$_4, observed_|) = 0.28 SE. This graph suggests that Takarkori traces around 73% of its ancestry to a hitherto unknown North African population, and the remaining (27%) is derived from an ancient Levantine source (Natufian-like ancestry). The admixture graph matrix for this model is available in Supp. Data 6 (model 2).
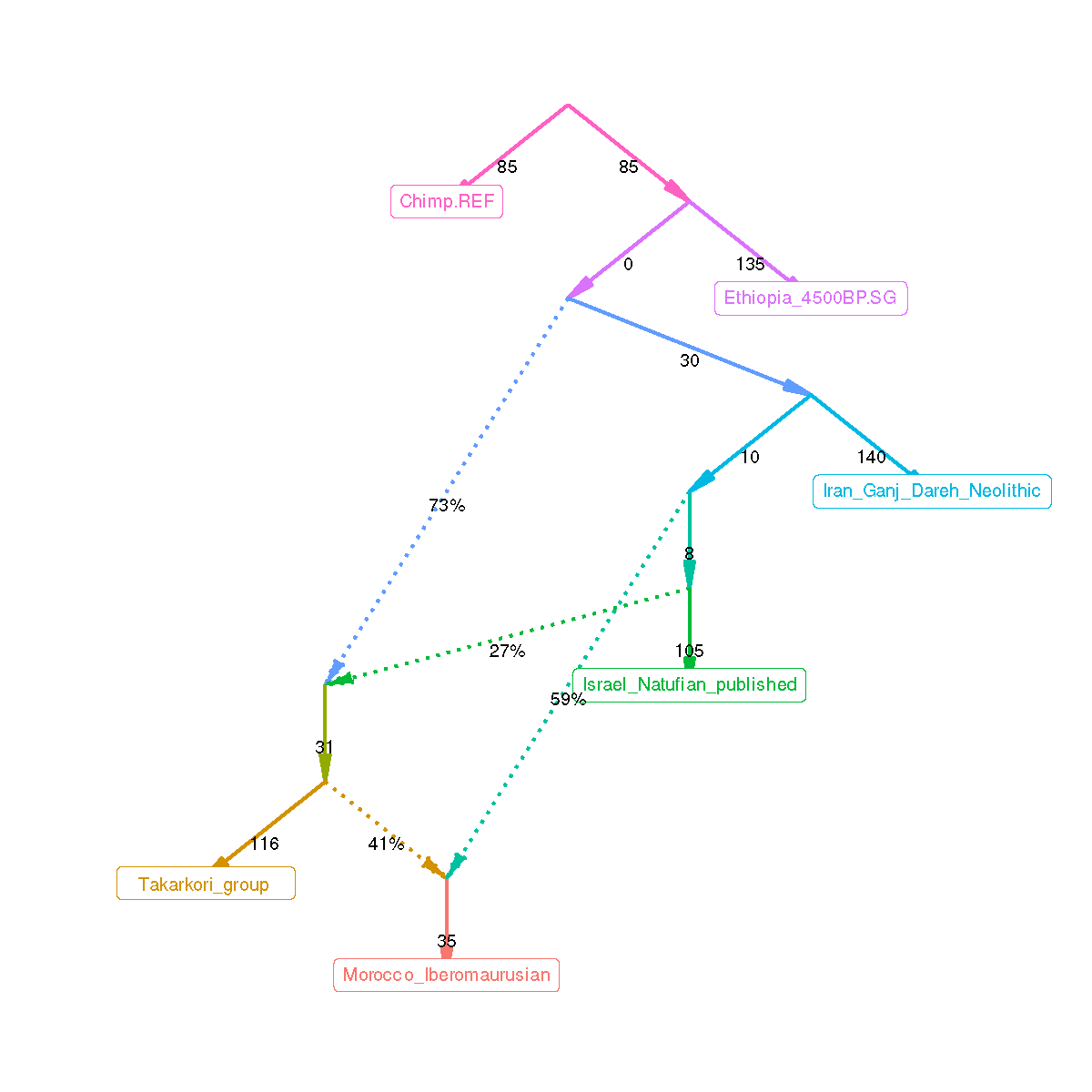


####


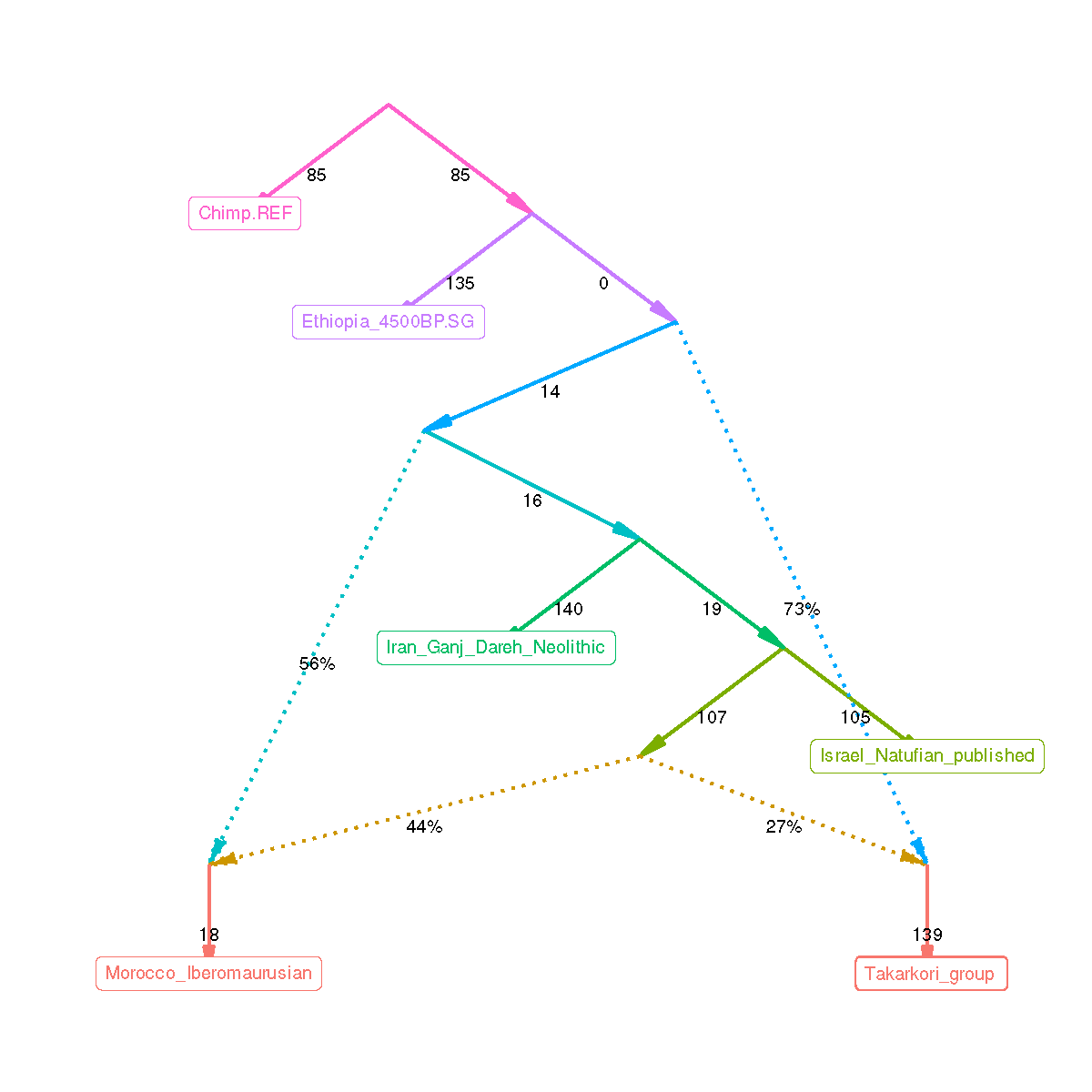


**Supplementary Figure 2.25:** Alternative admixture graph modeling Takarkori's ancestral relationship with relevant populations. The model fits with *f*-statistics residuals Z-score (max |$f$_4, expected_ – $f$_4, observed_|) = 1.23 SE. This graph suggests that Takarkori traces around 73% of its ancestry to a hitherto unknown North African population, and the remaining (27%) is derived from an ancient Levantine source (Natufian-like ancestry). The admixture graph matrix for this model is available in Supp. Data 6 (model 3).

### DATES

We applied the DATES[^54^](https://paperpile.com/c/AGF0JC/32GQ) method (version 753) to detect recent admixture events in low-coverage ancient genome data, using specific parameters (binsize = 0.001, fit range = 0.0045 to 1 in Morgan units). DATES works by first estimating the global admixture proportion in an individual, then calculating the genotype residuals and correcting for allele coding differences. The method analyzes how the correlation between SNP pairs decays with genetic distance, fitting an exponential decay curve to estimate admixture dates. It also combines SNP data across individuals to increase accuracy. Admixture dates are expressed in generations, assuming 28 years per generation.

To estimate Levantine admixture in Takarkori, using sub-Saharan populations (Yoruba, Mbuti, Dinka, ancient groups from Ethiopia and Tanzania) as well as Eurasian populations from Israel (Natufian), Jordan (PPNB), and Iran (Ganj Dareh) in a two-source model. The inferred admixture decay pattern of linkage disequilibrium showed less pronounced curvature with large standard errors, suggesting very ancient admixture events (**Supp. Fig. 2.26.1 - 2.26.3**).

**Supplementary Figure 2.26.1:** Admixture decay curves for Takarkori individuals with sub-Saharan and Eurasian populations, showing the decay of linkage disequilibrium over genetic distance. These decay curves are fitted to estimate the timing of admixture events.
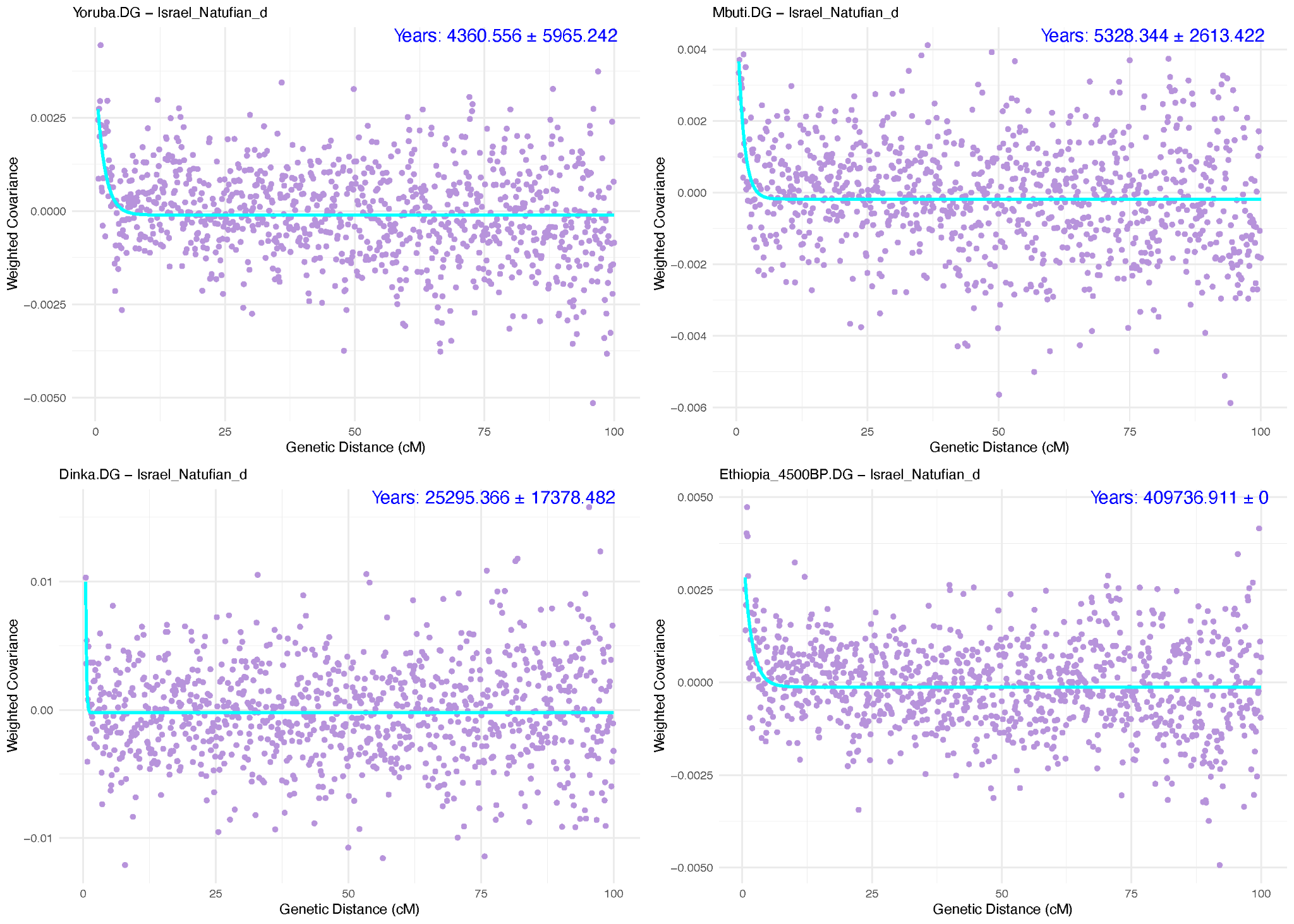


**Supplementary Figure 2.26.2:** Admixture decay curves for Takarkori individuals with sub-Saharan and Eurasian populations, showing the decay of linkage disequilibrium over genetic distance. These decay curves are fitted to estimate the timing of admixture events.
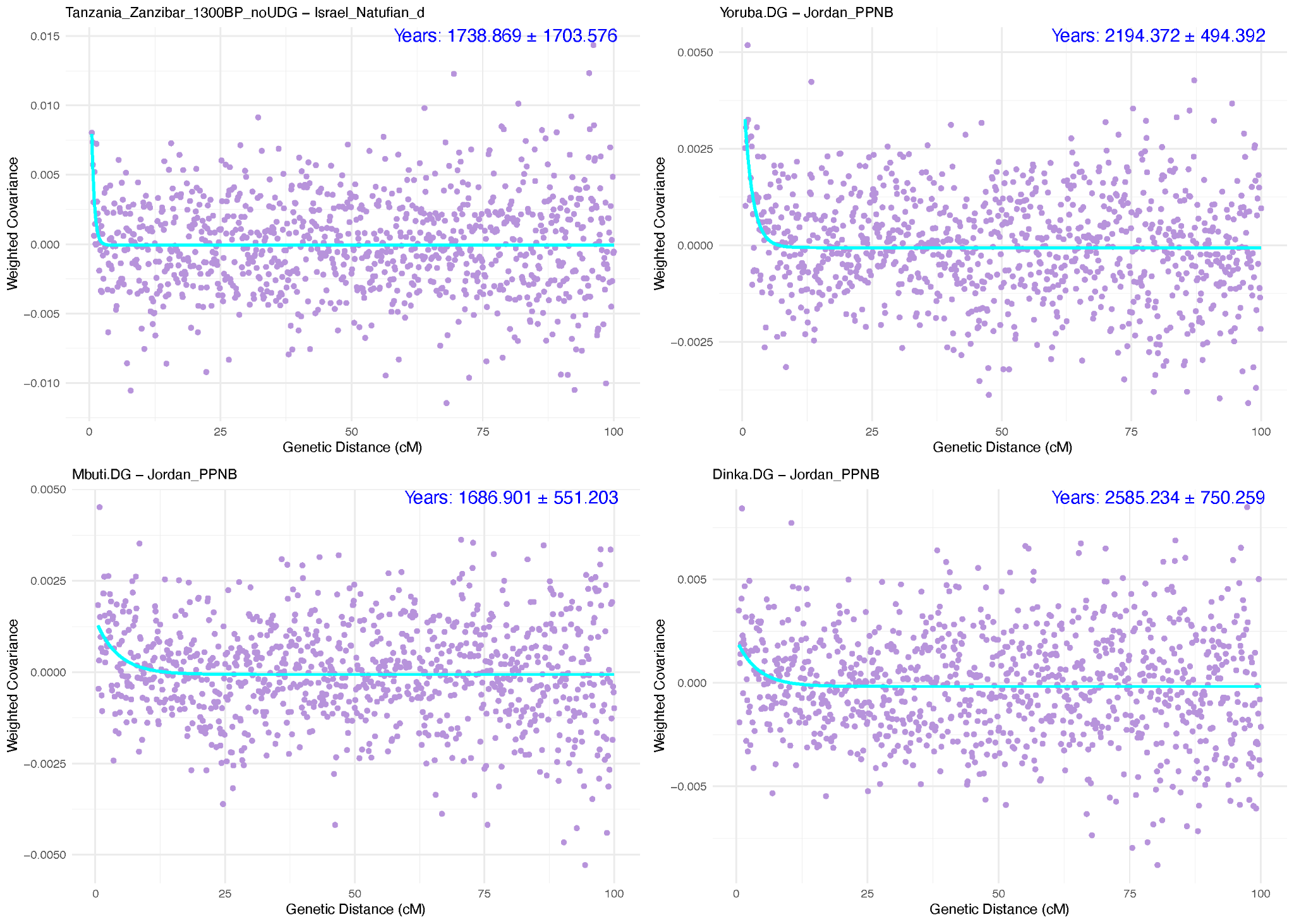


**Supplementary Figure 2.26.3:** Admixture decay curves for Takarkori individuals with sub-Saharan and Eurasian populations, showing the decay of linkage disequilibrium over genetic distance. These decay curves are fitted to estimate the timing of admixture events.
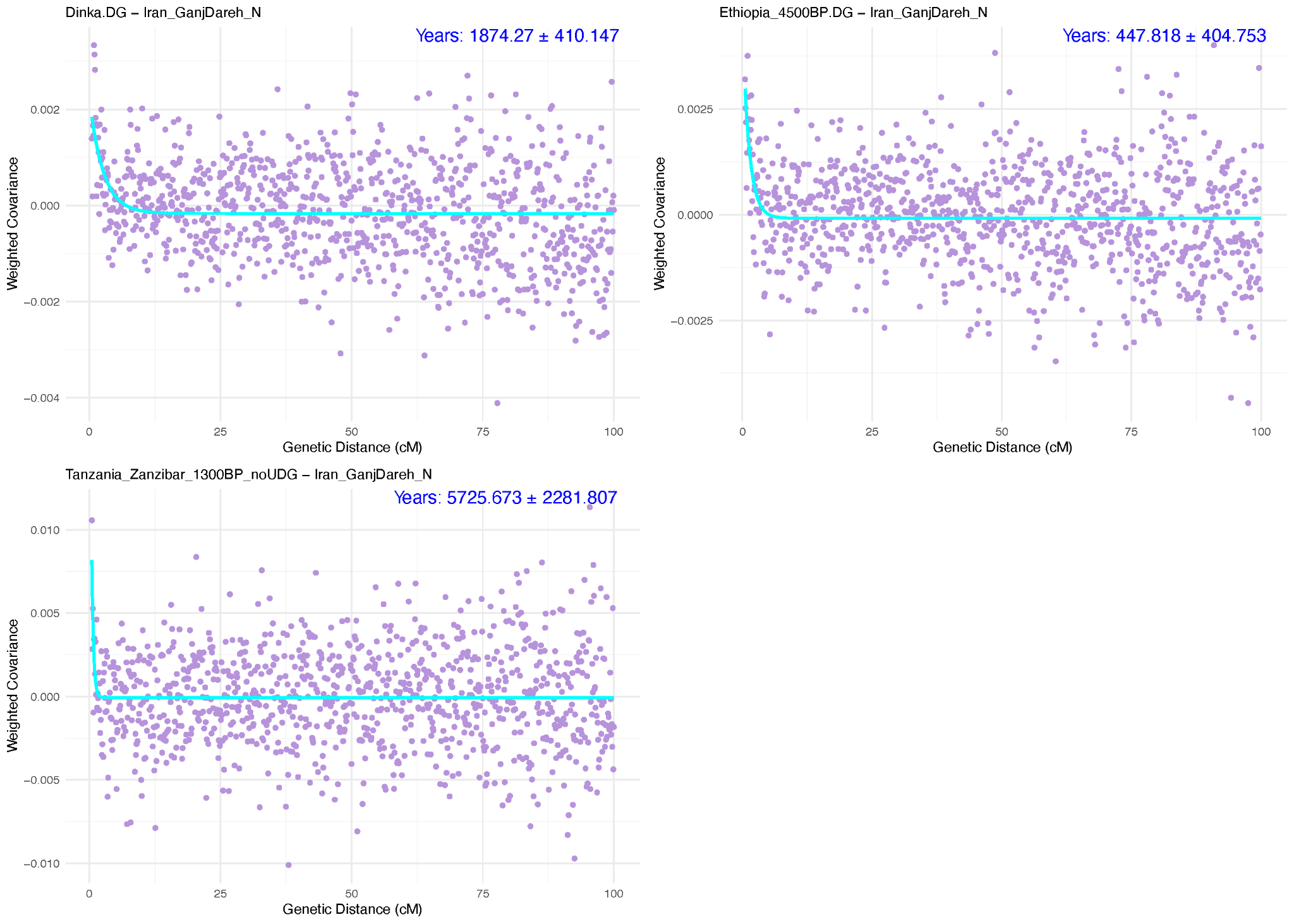


### Estimation of effective population size *N_e_* with hapROH

We estimated the effective population size *N*_e_ for a TKH001 using hapROH[^55^](https://paperpile.com/c/AGF0JC/GhPx) version 3.0. We first inferred ROH longer than 4 cM in TKH001, using hapROH’s recommended default settings and standard reference panel (the 1000G dataset). This analysis revealed five short and intermediate length ROH segments TKH001, of which two are 4-8 cM long and three 8-12cM (**Supp. Fig. 2.27**).

Using its maximum likelihood tool with default parameters and fitting inferred ROH between 4 to 16 cM long to theoretically predicted ROH in that length range yielded an *N*_e_ of 1,020, with a 95% confidence interval of 470 to 2,860, indicating a moderately sized population (**Supp. Fig. 2.28**). This estimate, intermediate between typical values for Eurasian Neolithic individuals and Hunter Gatherers[^55^](https://paperpile.com/c/AGF0JC/GhPx), suggests that the Takarkori population had enough members to prevent inbreeding of relatively close kin, consistent with a semi-nomadic pastoralist society. However, we stress that there is substantial sampling uncertainty when estimating *N*_e_ based on ROH of only a single individual, whose parents might be particularly in- or outbred.

**Supplementary Figure 2.27:** Inferred ROH segments >4 cM for TKH001. We depict their genomic location on each autosome (n=22). The Y-axis marks genomic map length measured in centimorgan.
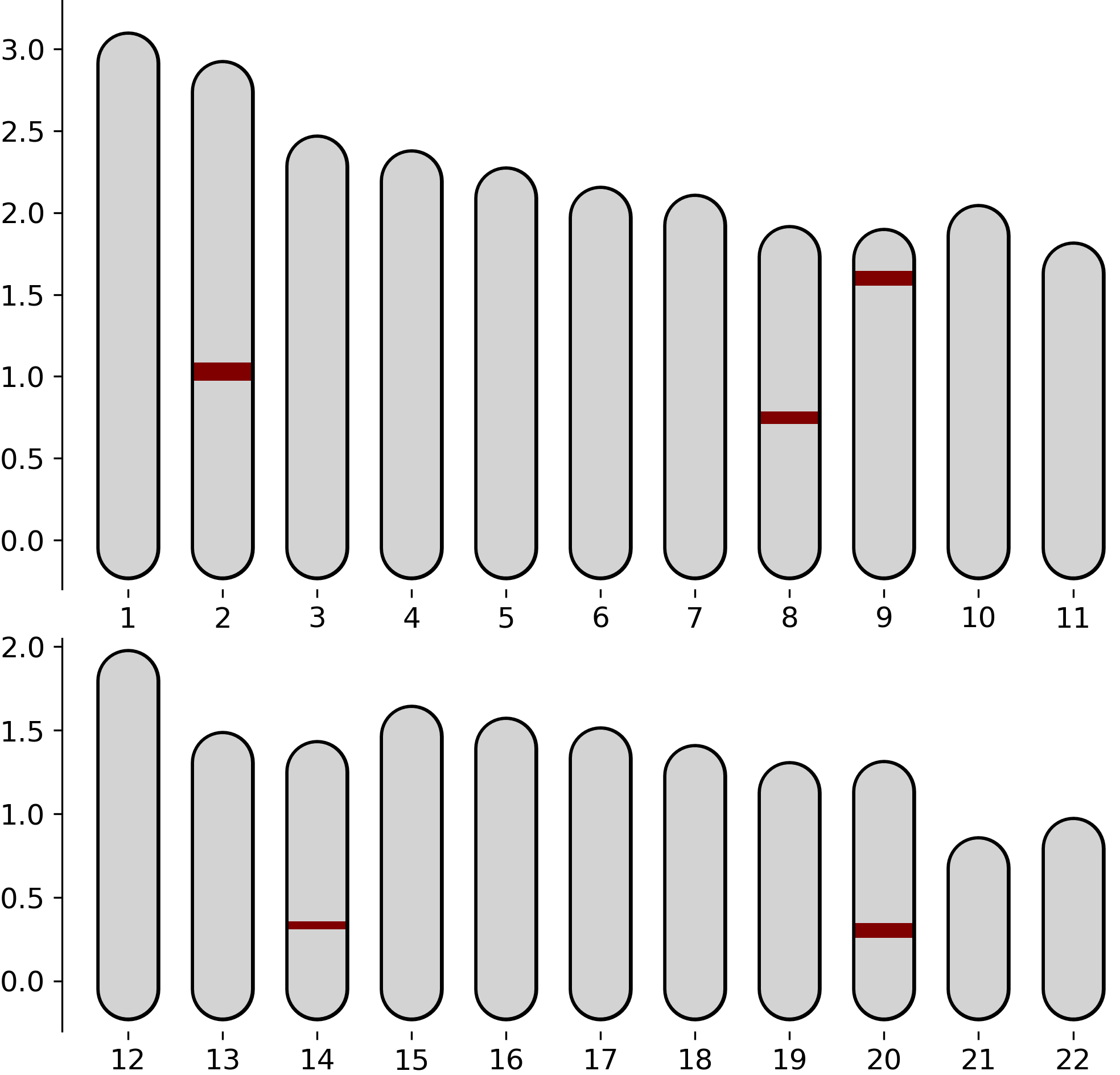


**Supplementary Figure 2.28:** Inferred ROH segments >4 cM for the Takarkori individual inferred with *hapROH*, compared to expected ROH distributions under scenarios of recent inbreeding loops (close-kin mating on the level of 1st, 2nd or 3rd cousin) and under scenarios of various small population sizes.
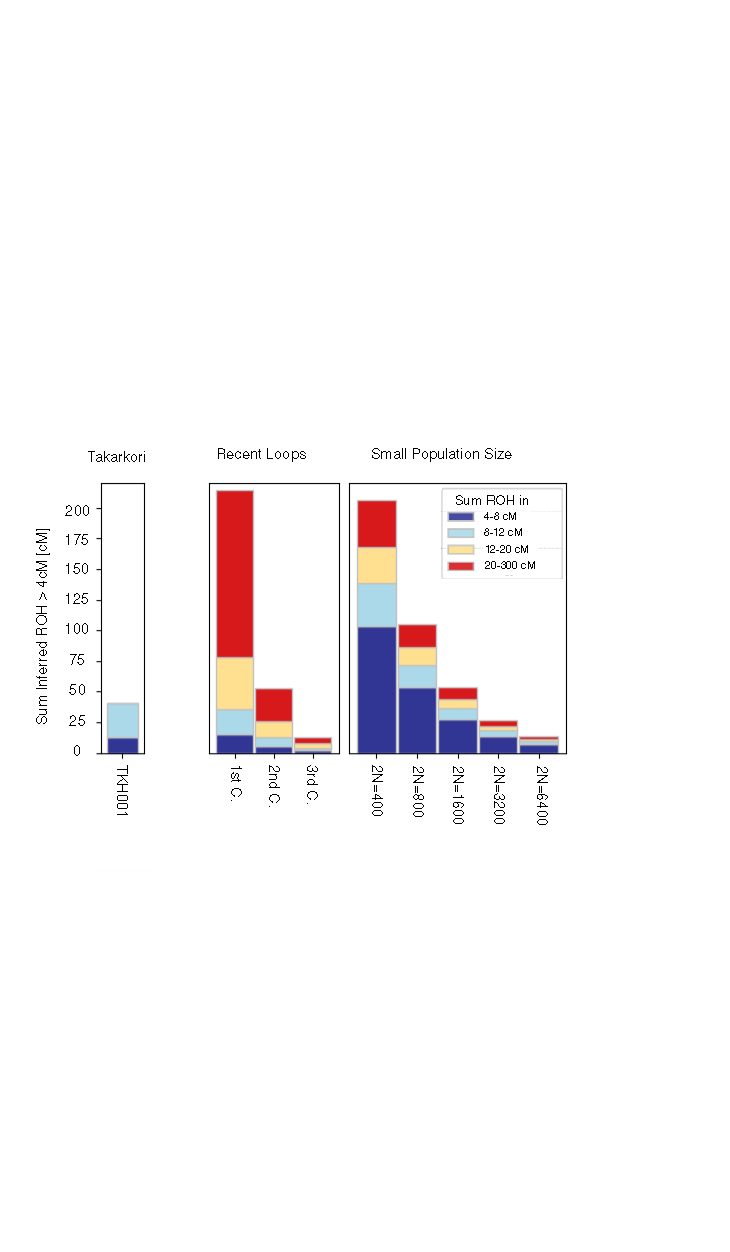


### ADMIXTURE

We conducted an unsupervised genetic clustering of global populations using ADMIXTURE version 1.3.0[^56^](https://paperpile.com/c/AGF0JC/gtzp). For this analysis, modern and ancient groups (Supp. Data 1) were subsetted from the HO-based Comprehensive SNP dataset into PLINK format using Trident Forge, which is part of the Poseidon framework[^57^](https://paperpile.com/c/AGF0JC/HBm1). The modern group dataset was transposed and converted to a pseudohaploid format to reduce the impact of artificial genetic drift in haploid ancient individuals on our clustering results. This was followed by linkage disequilibrium (LD) pruning with PLINK version 1.9[^58^](https://paperpile.com/c/AGF0JC/QTvI) using a window size of 200 SNPs, a step size of 25 SNPs, and an r² threshold of 0.4 (--indep-pairwise 200 25 0.4). Variants with a genotype missingness greater than 10% were excluded, leaving 406,330 SNPs to be analyzed. The pruned modern and ancient datasets were then merged into a final dataset. We ran five replicates with different random seeds for each K value, ranging from 2 to 9, and selected the replicate with the highest log-likelihood value. The most optimal level of cluster breakdown, as indicated by the lowest CV error, was K=7 (**Supp. Fig. 2.29**). Finally, the admixture results were visualized using the software AdmixturePlotter (<https://github.com/TCLamnidis/AdmixturePlotter>) (**Supp. Fig. 2.30**).

**Supplementary Figure 2.29:** Cross-validation (CV) error values as a function of the number of clusters (K) in ADMIXTURE analyses. For each K value, five replicates with random seeds were performed. The lowest CV error was observed for K=7.
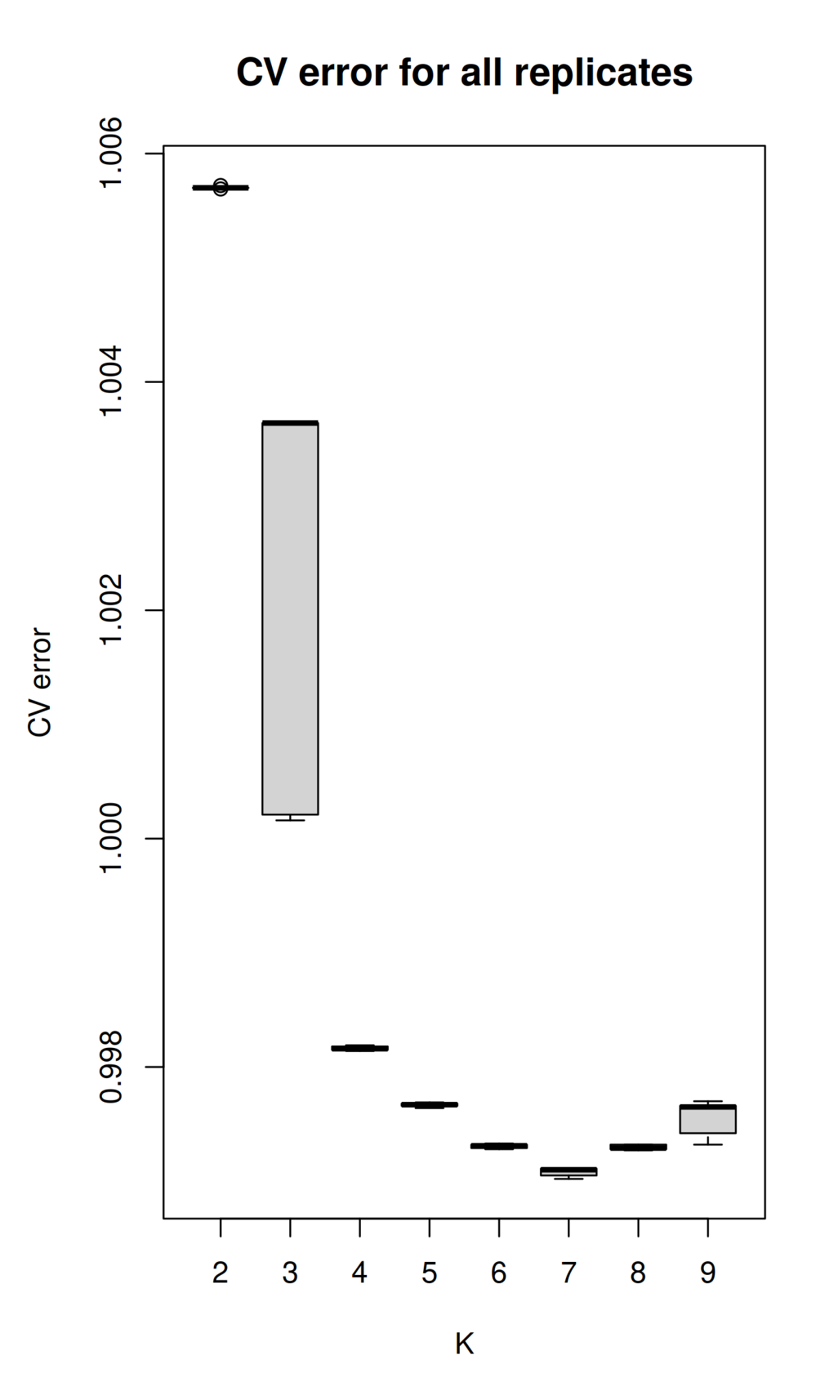


###
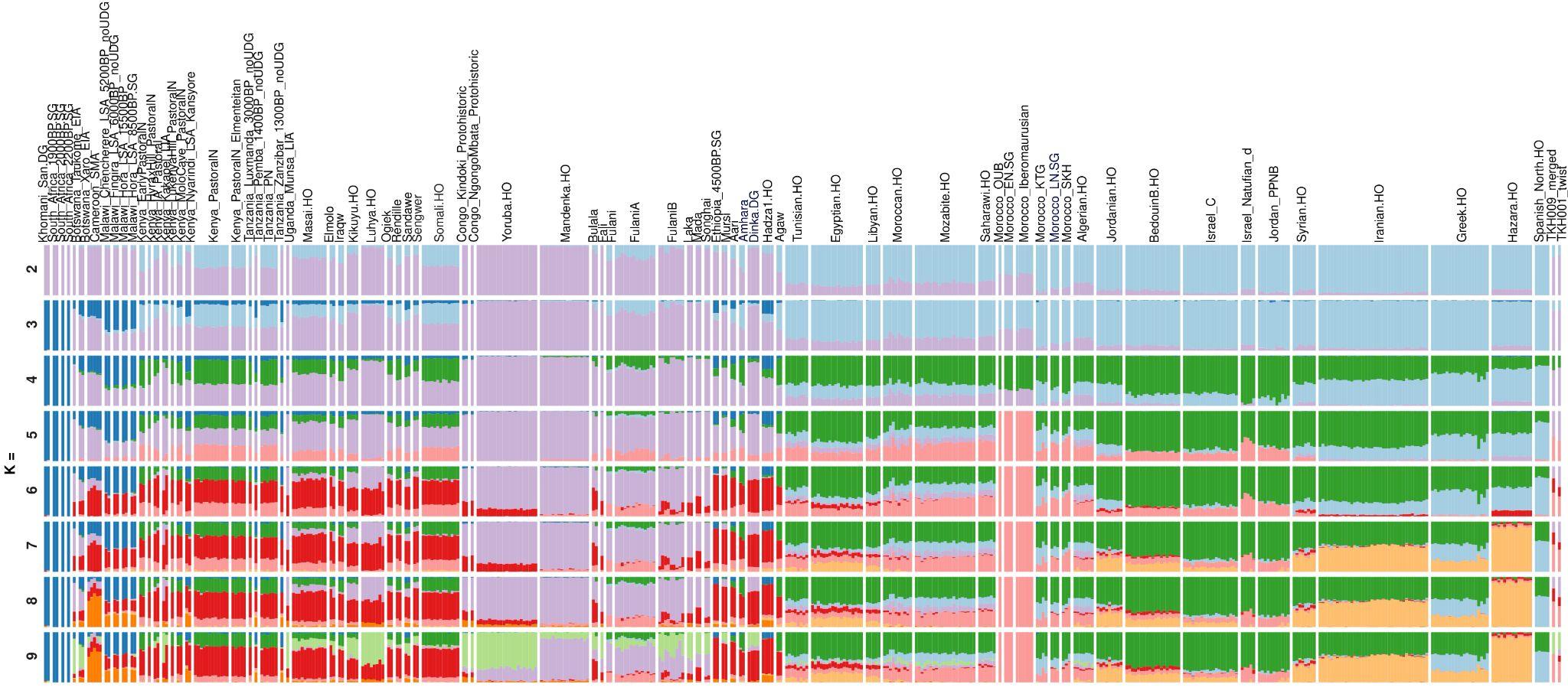


**Supplementary Figure 2.30:** Unsupervised ADMIXTURE results of modern and ancient populations, displayed for a few informative K values. The optimal K, determined by the lowest cross-validation error, is K=7. The Takarkori sample (TKH001_twist), a single sample from a deep ancestry population, was insufficient to model this component as a separate ancestry.

###

### Admixfrog

To infer Neanderthal ancestry, we used admixfrog version 0.7.1[^59^](https://paperpile.com/c/AGF0JC/0ACkh) on the single-stranded Takarkori library, belonging to the TKH001 individual, and the Taforalt libraries[^60^](https://paperpile.com/c/AGF0JC/L1AMf). These libraries underwent capture using the Archaic Admixture SNP panel, specifically Panel 4 as detailed in Fu, Hajdinjak et al. (2015)[^61^](https://paperpile.com/c/AGF0JC/YipHZ). For comparison, we included the following ancient high quality shotgun sequencing data from the Allen Ancient Genome Diversity Project/John Templeton Ancient DNA Atlas: ShumLakaI10871[^62^](https://paperpile.com/c/AGF0JC/SlHRu) and ShumLakaI10873[^62^](https://paperpile.com/c/AGF0JC/SlHRu), Mota[^63^](https://paperpile.com/c/AGF0JC/uFsyu), Jordan_AinGhazal_I1699[^51^](https://paperpile.com/c/AGF0JC/VxTvs), Iran_GanjDareh_I1290[^51^](https://paperpile.com/c/AGF0JC/VxTvs), Iran_GanjDareh_I1947[^51^](https://paperpile.com/c/AGF0JC/VxTvs) and Afanasievo_I6714[^64^](https://paperpile.com/c/AGF0JC/ADGUf), the recently published low-quality genomes of SKH001, SKH002, SKH003 and IAM004 from Neolithic Morocco[^45^](https://paperpile.com/c/AGF0JC/mk0Ju). Furthermore, we included the high coverage genome of the late Mesolithic individual from Bad Dürrenberg (Germany_DBD001)[^65^](https://paperpile.com/c/AGF0JC/zFdXo), the high coverage genome of LBK380, an ancient farmer who lived around 7,000 years ago in present day Stuttgart (LBK), and the high coverage genome of an individual who died approximately 8,000 years calBP in the Loschbour rockshelter in Luxembourg[^42^](https://paperpile.com/c/AGF0JC/PuJPM) (Loschbour). Additionally, we included present-day sub-Saharan genomes from the Dinka, Mandenka, Mbuti, San and Yoruba populations, as sourced from the Human Genome Diversity Project (HGDP) - specifically, Panel B in Prüfer et al. 2014[^66^](https://paperpile.com/c/AGF0JC/PK23O) in our analysis.

We subsetted all genomes to positions covered in the archaic admixture capture array, applying a filter for mapping quality of 25 and minimum length of 30 base pairs. We used AuthentiCT[^37^](https://paperpile.com/c/AGF0JC/Snl0k) v.1.0 to estimate contamination in the new data produced by this study’s archaic admixture captures. Due to low SNP coverage and high levels of contamination, we excluded Taforalt9, Taforalt12 and Taforalt15 from further analysis (**Supp. Table 2.8**). In addition, to make them more comparable with the Takarkori genome, we subsetted all high coverage genomes included in this analysis for comparison to the positions covered in the Takarkori genome.

**Supplementary Table 2.8 :** Coverage and contamination estimated per specimen after filtering.

| **Specimen** | **SNP coverage** | **Contamination (mean)** | **Contamination (st. err.)** |
| --- | --- | --- | --- |
| Takarkori | 475,501 | 0.001 | 0.00912 |
| Taforalt9 | 77,026 | 0.171916 | 0.007671 |
| Taforalt10 | 537,939 | 0.001 | 0.004344 |
| Taforalt11 | 106,9669 | 0.001 | 0.004162 |
| Taforalt12 | 113,275 | 0.007385 | 0.006289 |
| Taforalt13 | 855,909 | 0.001 | 0.003677 |
| Taforalt14 | 837,705 | 0.001 | 0.003523 |
| Taforalt15 | 41,761 | 0.105092 | 0.006584 |

Our reference panel included the three high-coverage Neanderthals[^66–68^](https://paperpile.com/c/AGF0JC/PK23O+KfGZD+QkkT1) and the high-coverage Denisovan[^69^](https://paperpile.com/c/AGF0JC/4OGDo) as the archaic references, and chimpanzee reference genome (panTro4) as the putative ancestral allele. For the African state, we used the Sub-Saharan African 1000 Genomes sequences[^70^](https://paperpile.com/c/AGF0JC/JzVfK).

Exact admixfrog command ran for Takarkori was:

admixfrog --infile TKH001.A0101_AA_l30_mq25.in.xz --ref ref_archaicadmixture.csv.xz -o TKH001.A0101_AA_l30_mq25 \

--states AFR NEA DEN --cont-id AFR --ll-tol 1e-2 --bin-size 5000 \

--est-F --est-tau --freq-F 3 --freq-contamination 3 --e0 1e-2 --est-error \

--ancestral PAN --run-penalty 0.1 --max-iter 250 --n-post-replicates 200 \

--filter-pos 50 --filter-map 0.000 --init-guess AFR

We detect twelve Neanderthal fragments that exceed a length of 0.05 cM (approximately 50kb) - a cutoff previously suggested to restrict further analysis to confidently called fragments that are very likely originating from Neanderthal admixture[^71,72^](https://paperpile.com/c/AGF0JC/6aRTU+9XYOa) (**Supp. Figure 2.31**). To further investigate these fragments, we examined the longest fragment located on chromosome 1, measuring 0.505 cM in length. As expected,Neanderthal ancestry detected in Takarkori within this region is absent from the African genomes that were used to construct the reference panel (**Supp. Figure 2.32**).

**
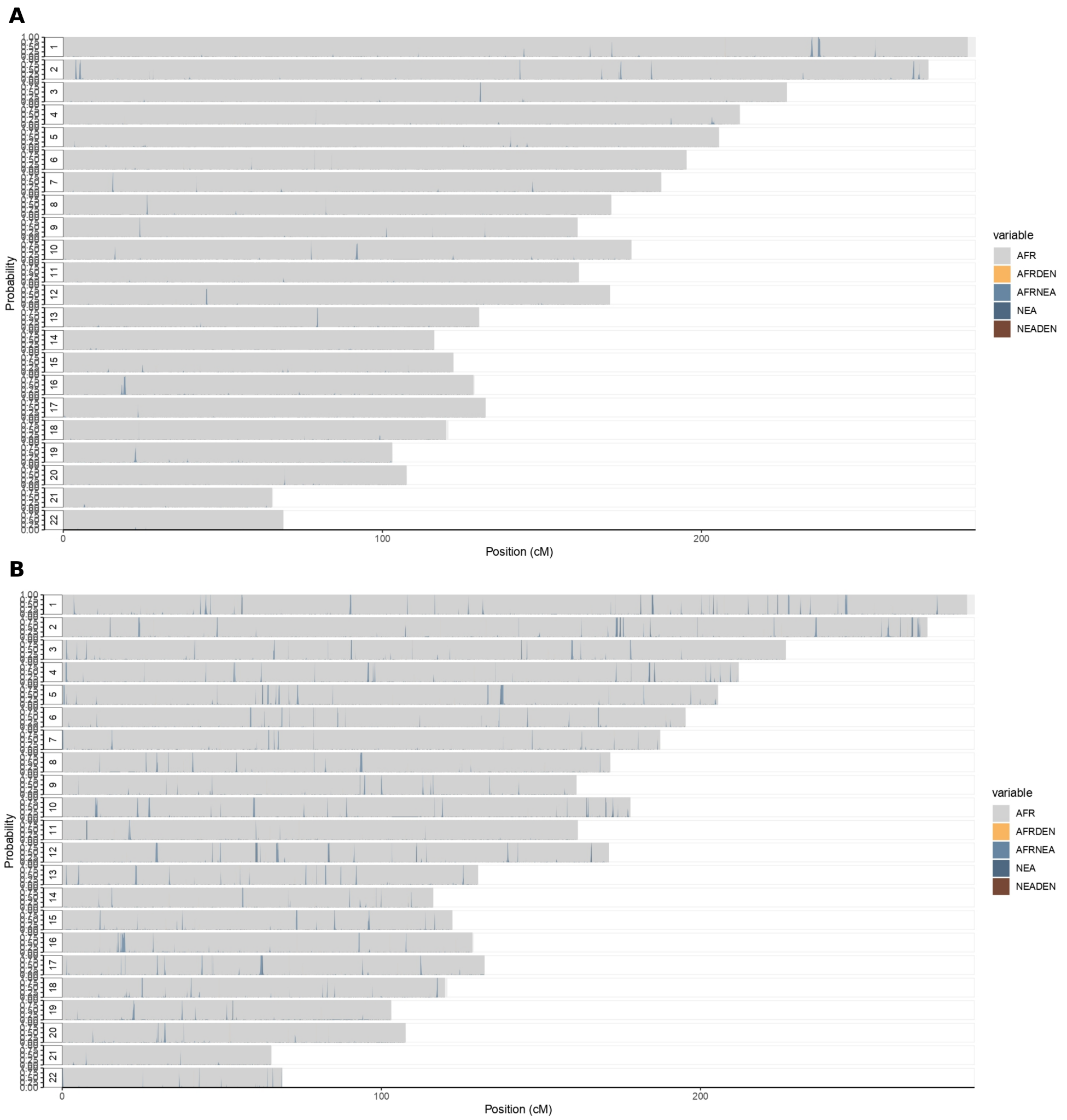
Supplementary Figure 2.31:** Detected archaic ancestry fragments in **A.** Takarkori and **B.** Taforalt11 (for comparison) on the autosomes, inferred by admixfrog. Dark and light blue regions are homozygous and heterozygous Neanderthal fragments, respectively. Grey fragments indicate African ancestry and bar height is proportional to the posterior probability of ancestry.


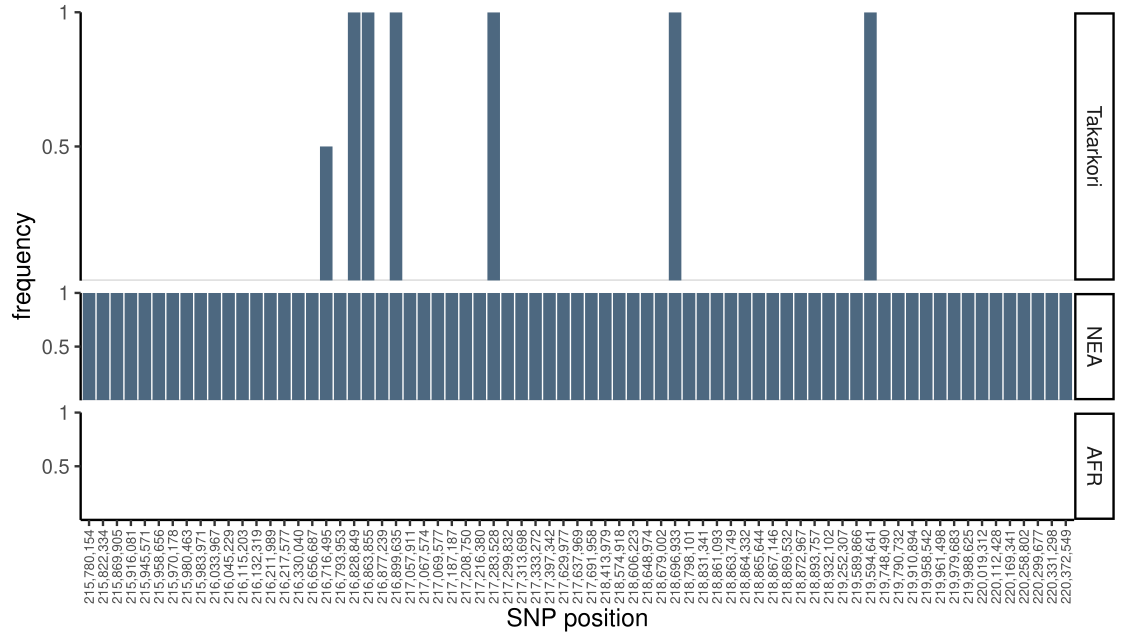


**Supplementary Figure 2.32:** Longest archaic fragment in Takarkori, on chromosome 1 in comparison to the reference panel. On the y-axis the frequency of the alternative allele in the reference population (Africans or Neanderthals in this case) at the given position is indicated, and the x-axis stands for the SNP positions.

#### **Estimating Neanderthal ancestry percentage**

Since the overall Neanderthal ancestry for Takarkori is very low, estimating the percentage of Neanderthal ancestry is difficult. For this purpose, we implemented a new algorithm in admixfrog that estimates the percentage of the genome that is in tracts of Neanderthal ancestry with a minimum length *L* (*L*=0.05cM throughout). We estimate this proportion from samples from the empirical-Bayes posterior, i.e. the probability Pr*(****Z*** *|* ***D****, θ_max_)*, where ***D*** = (D_1_, … D_n_) is the data, ***Z*** = (Z_1_, … Z_n_) is the sequence of hidden states and *θ*_max_ are the maximum-likelihood estimates of the parameters. This is not a fully-Bayesian approach because we do not take uncertainty *θ*_max_ into account. We estimate ***Z*** by simulating from the HMM, conditional on the data, using the following algorithm due to [^73^](https://paperpile.com/c/AGF0JC/aTO7H).

1. Use the stationary probabilities of the admixfrog HMM to draw *Z_0_*.
2. Calculate the transition probabilities, conditional on the data, using

$Pr(Z_{i}=z | Z_{0}, ...,Z_{i-1}, D) =\frac{\beta(Z_{i})}{\beta(Z_{i-1})}P(Z_{i}=z|Z_{i-1})P(D_{i}|Z_{i}=z)$.

Here, the $\beta(Z_{i})$ are the backwards probabilities, *P*(*Z*_i_ = *z*|*Z*_i-1_, *θ*_max_) are the transitions and *P*(*D*_i_|*Z*_i_ = z, *θ*_max_) are the emission probabilities. All three quantities are already computed in the main admixfrog algorithm and are easily available (For general background on HMMs, see e.g. [^74^](https://paperpile.com/c/AGF0JC/8Z0w9)).

1. Using these transition probabilities, we can draw the next state *Z*_i_.
2. Repeat step 2 for every bin.
3. Repeat the algorithm for all chromosome and for 200 iterations

From this output (stored in the .res-output file of admixfrog), we can estimate the proportion of Neanderthal ancestry in segments longer than *L* by counting the total number of such segments. Note that this is an estimate of the Neanderthal ancestry in fragments longer than *L*, which is expected to be an underestimate of the true proportion of Neanderthal ancestry.

We found the detectable Neanderthal ancestry in the Takarkori genome to be ~0.15%. This was less than a quarter of the Neanderthal ancestry found in Taforalt individuals (**Supp. Figure 2.33, Supp. Table 2.9**) but significantly more than the detectable Neanderthal ancestry in the other ancient and present-day African genomes.


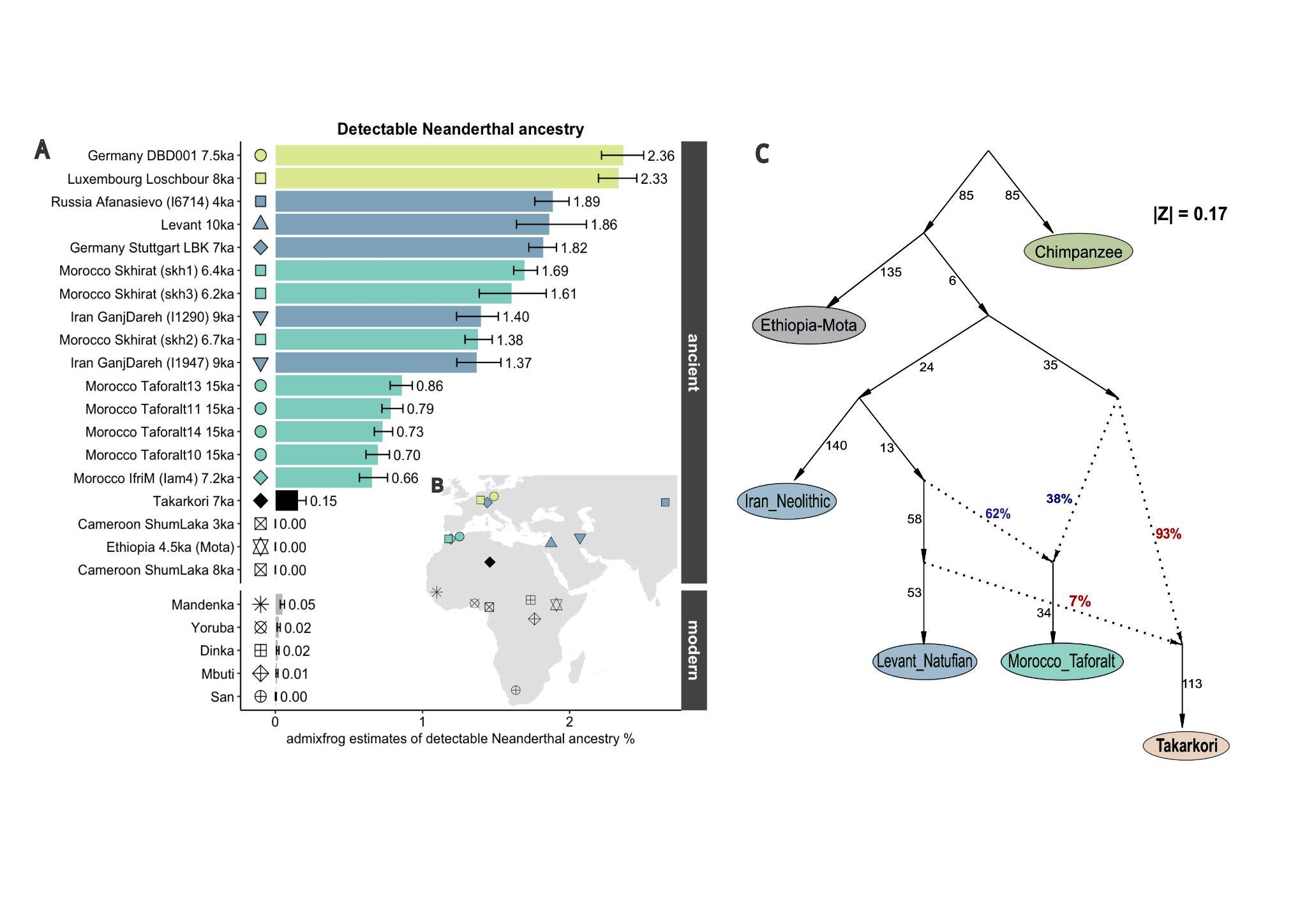


**Supplementary Figure 2.33: A)** Detectable Neanderthal ancestry in ancient groups from Africa and Eurasia, and present-day sub-Saharan African groups. Error bars represent minimum and maximum estimates from all iterations. **B)** Geographic locations of groups included in the analysis

The amount of Neanderthal ancestry we detected in the genomes of LBK and Loschbour aligns with previously published findings, obtained by f4 ratio test on 1240k data, standing at 2.0% (1.3-2.7 95% CI) and 2.2% (1.4-2.9, 95% CI) respectively[^75^](https://paperpile.com/c/AGF0JC/sqlaS). The proximity of our estimates to these earlier site-based values suggests that the length cutoff L does not lead to large-scale underestimates of Neanderthal ancestry.

We detected lower levels of overall Neanderthal ancestry in the genome of two Neolithic Iran individuals from Ganj Dareh. This is consistent with previous observation and is attributed to the comparably higher levels of the hypothesized basal Eurasian ancestry present in these genomes[^51^](https://paperpile.com/c/AGF0JC/VxTvs). Our admixfrog analysis of the four Taforalt genomes revealed Neanderthal ancestry estimates between 0.6% and 0.9%, which is roughly half of the Neanderthal ancestry estimate found in the Neolithic Levantine individual from Ain Ghazal in present-day Jordan. These estimates are in line with the results from qpAdm and qpGraph models where the Taforalt population was represented as having approximately 60-62% of Levantine-related ancestry best proxied by Natufian. Furthermore, the genome of the Jordan individual derives approximately two-thirds of its ancestry from the preceding Natufian ancestry[^51^](https://paperpile.com/c/AGF0JC/VxTvs). Consequently, the level of the detectable Neanderthal ancestry in the Natufian genome is comparable to that in Jordan, approximating 1.86%. In the case of Takarkori, our qpGraph analysis indicates ancestry received by a gene flow from the Levant, although the exact percentage remains intermediate, with an estimate of around 7%. This gene flow represents approximately 8% of the Neanderthal ancestry % found in the Jordan genome (1.86%), which corroborates our estimates using admixfrog. Furthermore, we found that the level of Neanderthal ancestry detectable in the Early Neolithic IAM4 genome was comparable to that of the Taforalt genomes. This is in line with the findings of Simoes et al., 2023[^45^](https://paperpile.com/c/AGF0JC/mk0Ju), which suggests that this population likely did not receive ancestry contributions from either Levant or Iberia.

In our analysis, we could not detect fragments of Neanderthal ancestry with a length of at least 0.05 cM in the ancient sub-Saharan African genomes from Shum Laka and Mota. Similarly, we found minimal to no evidence of such Neanderthal fragments in the present-day African populations from the sub-Saharan Africa we included in our study.

**Supplementary Table 2.9:** Neanderthal ancestry proportions per specimen plotted in Fig. 4C and Supp. Fig 2.15, with low and high limits. Number of confidently called fragments which are longer than 0.05 cM.

| **specimen** | **mean Neanderthal ancestry** | **CI low** | **CI high** | **Number of fragments >= 0.05 cM** |
| --- | --- | --- | --- | --- |
| Takarkori | 0.001531721 | 0.001113676 | 0.002087298 | 12 |
| Taforalt10 | 0.006962615 | 0.006161812 | 0.007756497 | 46 |
| Taforalt11 | 0.007859104 | 0.007245233 | 0.00867065 | 76 |
| Taforalt13 | 0.008606795 | 0.0078043 | 0.009310112 | 93 |
| Taforalt14 | 0.007277003 | 0.006729717 | 0.007974963 | 76 |
| SKH1 | 0.0169348600 | 0.0162015000 | 0.0178097700 | 352 |
| SKH2 | 0.0137741200 | 0.0128917000 | 0.0147439000 | 189 |
| SKH3 | 0.0160537900 | 0.0138582000 | 0.0184157500 | 37 |
| IAM4 | 0.0065602070 | 0.0057180760 | 0.0076271950 | 23 |
| ShumLakaI10871 | 7.13E-06 | 7.13E-06 | 7.13E-06 | 0 |
| ShumLakaI10873 | 7.84E-06 | 7.84E-06 | 7.84E-06 | 0 |
| Mota | 7.13E-06 | 7.13E-06 | 7.13E-06 | 0 |
| Jordan | 0.01861038 | 0.01639567 | 0.02114312 | 42 |
| Iran_GanjDareh_I1290 | 0.01396222 | 0.01231603 | 0.01515751 | 106 |
| Iran_GanjDareh_I1947 | 0.01368844 | 0.0123352 | 0.01533029 | 32 |
| Dinka | 0.000157488 | 7.54E-05 | 0.000250447 | 2 |
| Mandenka | 0.0004861366 | 0.000337939 | 0.0006210115 | 5 |
| Mbuti | 0.0001070319 | 4.20E-05 | 0.0001809199 | 1 |
| San | 2.58E-05 | 7.14E-06 | 6.28E-05 | 0 |
| Yoruba | 0.0002354106 | 0.0001287827 | 0.0003343915 | 2 |
| Loschbour | 0.02332816 | 0.02196333 | 0.02456486 | 302 |
| LBK | 0.01820075 | 0.01722777 | 0.01910679 | 236 |
| Germany_DBD001 | 0.02364898 | 0.02216822 | 0.02503481 | 262 |
| Afanasievo_I6714 | 0.01886194 | 0.01763672 | 0.01996284 | 220 |

##

### BEAST analysis of mitochondrial genomes

The study by Vai et al. 2019 [^24^](https://paperpile.com/c/AGF0JC/68BW) revealed that the TKH001 individual exhibits a mitochondrial haplogroup that had not been previously identified in Africa. This haplogroup is classified under a basal branch of haplogroup N, which diverged immediately after the Paleolithic 40,000-year-old Oase 1 individual and before all present-day N-derived mtDNAs. The validation of this result was accomplished through two methods: Median Joining Network (calculated by Network v.5, [www.fluxus-engineering.com](http://www.fluxus-engineering.com)) and Bayesian phylogenetic analyses (calculated by BEAST v.1.8.0[^76^](https://paperpile.com/c/AGF0JC/wlHl). The time to the most recent common ancestor (TMRCA) for the N clade, and the branch leading to the Takarkori sequences, was inferred to be 64,103 years BP (95% HPD: 54,535–74,578) and 61,618 years BP (95% HPD: 52,517–71,405) respectively, by BEAST v2.6.7[^77^](https://paperpile.com/c/AGF0JC/9vOql). The tip date for the terminal node of TKH001 was estimated to be 12,325 years BP (95% HPD: 1–25,119).

In our work, we performed BEAST analysis for mtDNA dating utilizing the same modern genomes that Vai et al. 2019[^24^](https://paperpile.com/c/AGF0JC/68BW), complemented by 49 ancient genomes. These 49 encompass all the 42 genomes from the aforementioned Vai et al. study, with the exception of the Doonside genome from Schlebusch et al. 2017[^78^](https://paperpile.com/c/AGF0JC/0S2AU) and the I2967_Hora, I4421_Chenchere, I4422_Chenchere, and I9133_SouthAfrica genomes from Skoglund et al. 2017[^79^](https://paperpile.com/c/AGF0JC/2Ziet). Instead, we added six individuals from Bacho Kiro, Bulgaria[^80,81^](https://paperpile.com/c/AGF0JC/nfV6t+IUO6U), one from Zlatý kůň, Czechia[^75^](https://paperpile.com/c/AGF0JC/sqlaS), one from Salkhit, Mongolia[^82^](https://paperpile.com/c/AGF0JC/VH3gd), and the GoyetQ116-1[^83^](https://paperpile.com/c/AGF0JC/3kUVj) sample, all of which are more than 30,000 years old, and have a basal branch of N haplogroup. The compiled dataset of these samples is listed in **Supp. Table 2.10**.

Our findings are in line with those of Vai et al. — our BEAST analysis estimates the TMRCA for the branch containing the Takarkori individuals (TKH001 and TKH009) to be 61,343 years old (95% HPD: 54,408–69,046). Moreover, the tip date for the shared node of TKH001 and TKH009 is approximated to be 12,938 years old (95% HPD: 4,988–21,238). By including additional mtDNA sequences from Upper Paleolithic individuals, we observed a narrowing of the confidence intervals for the TMRCA estimate of the Takarkori branch within the N clade. The corresponding phylogenetic tree is illustrated in **Supp. Fig. 2.34**. This figure was generated using the open-source FigTree v1.4.4 software (<http://tree.bio.ed.ac.uk/software/figtree/>).

##
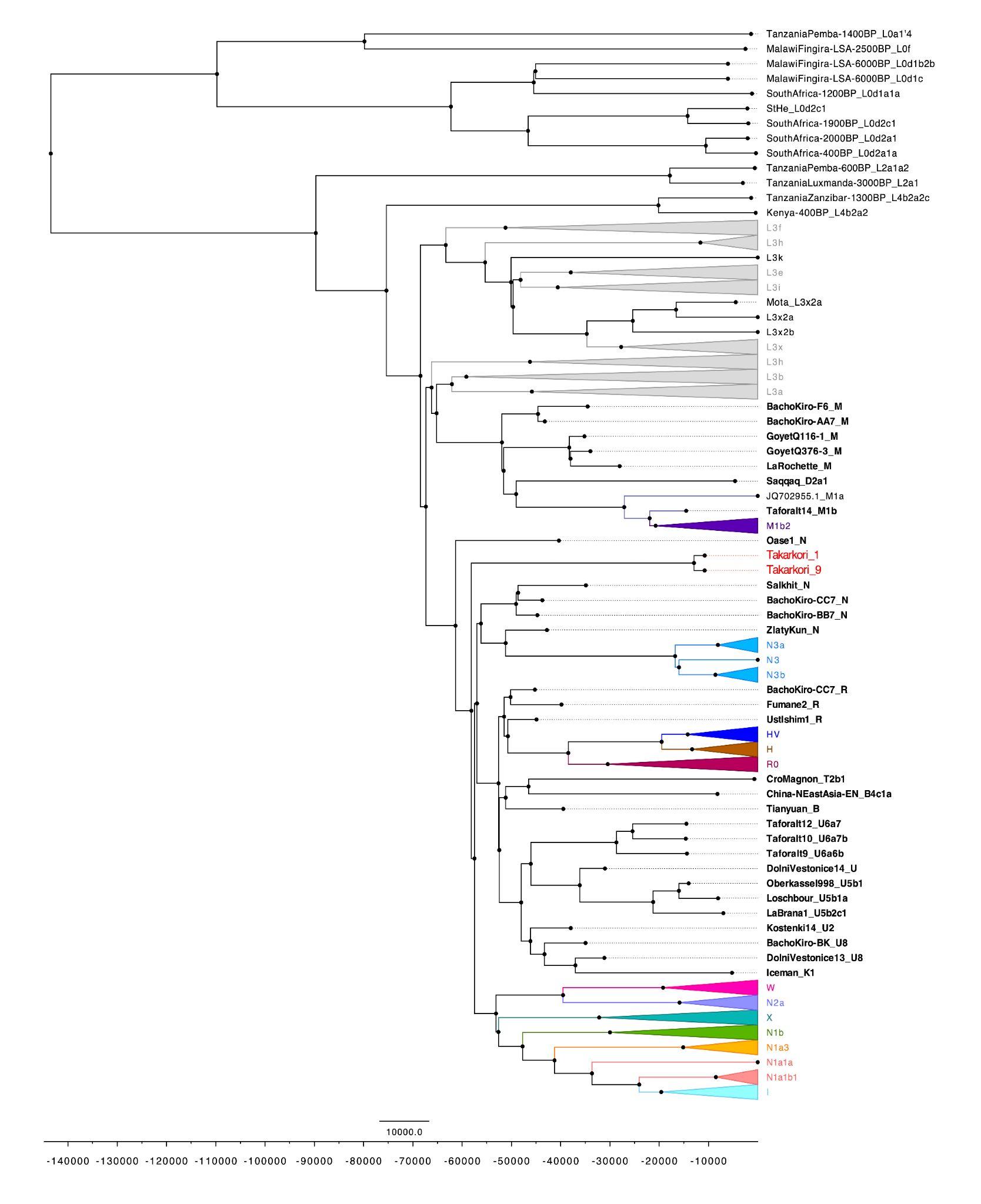


**Supplementary Figure 2.34:** Phylogenetic tree constructed for the Takarkori samples TKH001 and TKH009, in conjunction with 209 published complete genomes from both ancient and modern samples. The major mitochondrial lineages and sub-lineages for the N macrohaplogroup are differentiated by distinct colors.

**Supplementary Table 2.10:** Dataset of comparative samples used in the mtDNA dating analysis

| **label** | **sampleID** | **lower boundary [y BP]** | **upper boundary [y BP]** | **present in GenBank** | **publication** |
| --- | --- | --- | --- | --- | --- |
| StHe | KJ669158.1 | 1965 | 2241 | TRUE | Morris et al. (2004) |
| Iceman | EU810403.1 | 5100 | 5350 | TRUE | Ermini et al. (2008) |
| Saqqaq | EU725621.2 | 4410 | 4790 | TRUE | Gilbert et al. (2008) |
| Kostenki_14 | FN600416.1 | 37320 | 38650 | TRUE | Krause et al. (2010) |
| La_Brana_1 | JX186998.1 | 6930 | 7030 | TRUE | Sanchez-Quinto et al. (2012) |
| Tianyuan | KC417443.1 | 38830 | 40120 | TRUE | Fu et al. (2013) |
| BS11 | KC521454.1 | 8040 | 8320 | TRUE | Fu, Mittnik et al. (2013) |
| CroMagnon | KC521456.1 | 651 | 729 | TRUE | Fu, Mittnik et al. (2013) |
| DolniVestonice13 | KC521459.1 | 31030 | 31240 | TRUE | Fu, Mittnik et al. (2013) |
| DolniVestonice14 | KC521458.1 | 30860 | 31150 | TRUE | Fu, Mittnik et al. (2013) |
| Loschbour | KC521455.1 | 7927 | 8181 | TRUE | Fu, Mittnik et al. (2013) |
| Oberkassel998 | KC521457.1 | 13870 | 14170 | TRUE | Fu, Mittnik et al. (2013) |
| UstIshim1 | UstIshim1 | 43210 | 46880 | FALSE | Fu et al. (2014) |
| Fumane2 | KP718913.1 | 38500 | 41110 | TRUE | Benazzi et al. (2015) |
| Oase1 | Oase1 | 39410 | 41470 | FALSE | Fu et al. (2015) |
| Mota | Mota | 4418 | 4524 | FALSE | Llorente et al. (2015) |
| GoyetQ116-1 | KU534952.1 | 34720 | 35630 | TRUE | Posth et al. (2016) |
| GoyetQ376-3 | KU534953.1 | 33450 | 34550 | TRUE | Posth et al. (2016) |
| LaRochette | KU534951.1 | 26910 | 29000 | TRUE | Posth et al. (2016) |
| Hohlenstein-Stadel | KY751400.1 |  |  | TRUE | Posth et al. (2017) |
| Ballito_Bay_A | baa001 | 1831 | 1986 | FALSE | Schlebusch et al. (2017) |
| Ballito_Bay_B | bab001 | 1932 | 2149 | FALSE | Schlebusch et al. (2017) |
| Champagne_Castle | cha001 | 282 | 448 | FALSE | Schlebusch et al. (2017) |
| Eland_Cave | ela001 | 453 | 533 | FALSE | Schlebusch et al. (2017) |
| Mfonfosi | mfo001 | 308 | 448 | FALSE | Schlebusch et al. (2017) |
| Newcastle | new001 | 327 | 508 | FALSE | Schlebusch et al. (2017) |
| I0589_Zanzibar | I0589_merged | 1303 | 1370 | FALSE | Skoglund et al. (2017) |
| I0595_Kenya | I0595_merged | 322 | 496 | FALSE | Skoglund et al. (2017) |
| I1048_Pemba | I1048_merged | 1307 | 1421 | FALSE | Skoglund et al. (2017) |
| I2298_Pemba | I2298 | 544 | 639 | FALSE | Skoglund et al. (2017) |
| I3726_Luxmanda | I3726 | 2890 | 3141 | FALSE | Skoglund et al. (2017) |
| I4426_Fingira | I4426 | 2330 | 2676 | FALSE | Skoglund et al. (2017) |
| I4427_Fingira | I4427 | 5913 | 6175 | FALSE | Skoglund et al. (2017) |
| I4468_Fingira | I4468 | 5923 | 6177 | FALSE | Skoglund et al. (2017) |
| I9134_South_Africa | I9134 | 1069 | 1282 | FALSE | Skoglund et al. (2017) |
| TAF009 | MG936619.1 | 13908 | 14805 | TRUE | van der Loosdrecht et al. (2018) |
| TAF010 | MG936620.1 | 14132 | 15077 | TRUE | van der Loosdrecht et al. (2018) |
| TAF012 | MG936622.1 | 14049 | 14890 | TRUE | van der Loosdrecht et al. (2018) |
| TAF014 | MG936624.1 | 14120 | 14956 | TRUE | van der Loosdrecht et al. (2018) |
| Salkhit | Salkhit | 34330 | 35410 | FALSE | Treviese et al. (2019) |
| TKH-1 | MF479727.1 | 0 | 20000 | TRUE | Vai et al. (2019) |
| TKH-9 | MF479728.1 | 0 | 20000 | TRUE | Vai et al. (2019) |
| BachoKiro_AA7_738 | MN706602.1 | 42580 | 43930 | TRUE | Hublin et al. (2020) |
| BachoKiro_BB7_240 | MN706603.1 | 43940 | 45550 | TRUE | Hublin et al. (2020) |
| BachoKiro_BK 1653 | MN706604.1 | 34610 | 35290 | TRUE | Hublin et al. (2020) |
| BachoKiro_CC7_2289 | MN706606.1 | 44420 | 45930 | TRUE | Hublin et al. (2020) |
| BachoKiro_CC7_335 | MN706605.1 | 42990 | 44400 | TRUE | Hublin et al. (2020) |
| BachoKiro_molar_F6_620 | MN706607.1 | 34210 | 34820 | TRUE | Hublin et al. (2020) |
| ZlatyKun | ZlatyKun | 25000 | 50000 | FALSE | Pruefer et al. (2021) |

###

#### **Technical details of BEAST analysis**

In addition to the comparative samples listed in **Supp. Table 2.10**, we selected the mtDNA genome of the Hohlenstein-Stadel Neanderthal[^84^](https://paperpile.com/c/AGF0JC/1DOex) as the outgroup. For samples for which no mtDNA sequence was publicly available on NCBI GenBank, their sequencing data were processed through a specialized pipeline (<https://github.com/alexhbnr/mitoBench-ancientMT>)[^85^](https://paperpile.com/c/AGF0JC/ZscSS). In this pipeline, the mtDNA consensus sequences were determined using the ancient DNA damage-aware genotyper snpAD v0.3.9[^86^](https://paperpile.com/c/AGF0JC/HdOaS), requiring a minimum coverage of 3-fold and mtDNA haplogroups were assigned using HaploGrep2 v2.1.19[^87^](https://paperpile.com/c/AGF0JC/yUaBe).

We prepared a multi-sequence alignment of all 216 mtDNA sequences using MAFFT v7.508[^88^](https://paperpile.com/c/AGF0JC/DwaGZ) with a maximum of 10 iterations and subsequently removed the poly-C regions located at positions 303-315 and 16,182-16,193. This alignment was used as input into BEAST v2.6.7 [^77^](https://paperpile.com/c/AGF0JC/9vOql) while providing the 95% confidence intervals of the calibrated radiocarbon dates of the ancient samples (**Supp. Table 2.10**) as uniform priors for estimating the clock rate. For ancient DNA samples without radiocarbon dates, such as Zlatý kůň, and the two individuals from the Takarkori rock shelter, we set wider uniform priors to estimate their age. To determine the most suitable model for the dating analysis, we first evaluated the fit of the substitution models using bModelTest v1.2.1[^89^](https://paperpile.com/c/AGF0JC/HzowK) while setting the clock model to a strict clock rate and the tree prior to “coalescent constant population”. We used the most appropriate substitution model, TIM(21)+I+G, subsequently for determining whether a strict or a relaxed log-normal clock and whether a “coalescent constant population” or a “coalescent Bayesian skyline” tree prior were better suited for the underlying data. Using path sampling as implemented in the BEAST package Model Selection v1.5.3 (number of steps: 30; chain length: 10 million iterations), we obtained marginal likelihood estimates for each of the combinations of the clock rate model and tree prior. We determined through Bayes factor analysis [^90^](https://paperpile.com/c/AGF0JC/gDRNN) that a strict clock rate and a “coalescent Bayesian skyline” tree prior were the most suited combination. Using this combination of models (TIM(21)+I+G, strict clock rate, “coalescent Bayesian skyline” tree prior), we ran eight independent analyses with 75 million iterations storing the trees of every 25,000th iteration. We combined the trees of all eight runs using LogCombiner, discarding the initial 10% of the trees as burn-in and generated a maximum clade credibility tree with “common ancestor” node heights using TreeAnnotator.

# References

1. [deMenocal, P., Ortiz, J., Guilderson, T. & Sarnthein, M. Coherent high- and low-latitude climate variability during the holocene warm period. *Science* **288**, 2198–2202 (2000).](http://paperpile.com/b/AGF0JC/tpNO3)

2. [Hoelzmann, P. *et al.* Palaeoenvironmental changes in the arid and sub arid belt (Sahara-Sahel-Arabian Peninsula) from 150 kyr to present. in *Past Climate Variability through Europe and Africa* (eds. Battarbee, R. W., Gasse, F. & Stickley, C. E.) 219–256 (Springer Netherlands, Dordrecht, 2004).](http://paperpile.com/b/AGF0JC/x2S0j)

3. [Kutzbach, J. E. & Street-Perrott, F. A. Milankovitch forcing of fluctuations in the level of tropical lakes from 18 to 0 kyr BP. *Nature* **317**, 130–134 (1985).](http://paperpile.com/b/AGF0JC/kyWiy)

4. [Cheddadi, R. *et al.* Early Holocene greening of the Sahara requires Mediterranean winter rainfall. *Proc. Natl. Acad. Sci. U. S. A.* **118**, (2021).](http://paperpile.com/b/AGF0JC/QbemM)

5. [Kröpelin, S. *et al.* Climate-driven ecosystem succession in the Sahara: the past 6000 years. *Science* **320**, 765–768 (2008).](http://paperpile.com/b/AGF0JC/P3dfE)

6. [Cremaschi, M. *et al.* Takarkori rock shelter (SW Libya): an archive of Holocene climate and environmental changes in the central Sahara. *Quat. Sci. Rev.* **101**, 36–60 (2014).](http://paperpile.com/b/AGF0JC/OgCT)

7. [Tierney, J. E., Pausata, F. S. R. & deMenocal, P. B. Rainfall regimes of the Green Sahara. *Sci Adv* **3**, e1601503 (2017).](http://paperpile.com/b/AGF0JC/DAO70)

8. [Van Neer, W. *et al.* Aquatic fauna from the Takarkori rock shelter reveals the Holocene central Saharan climate and palaeohydrography. *PLoS One* **15**, e0228588 (2020).](http://paperpile.com/b/AGF0JC/Yiag)

9. [Cremaschi, M. & Di Lernia, S. Holocene Climatic Changes and Cultural Dynamics in the Libyan Sahara. *African Archaeological Review* **16**, 211–238 (1999).](http://paperpile.com/b/AGF0JC/hD86q)

10. [Mercuri, A. M., Fornaciari, R., Gallinaro, M., Vanin, S. & di Lernia, S. Plant behaviour from human imprints and the cultivation of wild cereals in Holocene Sahara. *Nat Plants* **4**, 71–81 (2018).](http://paperpile.com/b/AGF0JC/j1s8t)

11. [di Lernia, S. Dismantling Dung: Delayed Use of Food Resources among Early Holocene Foragers of the Libyan Sahara. *Journal of Anthropological Archaeology* **20**, 408–441 (2001).](http://paperpile.com/b/AGF0JC/hOAVC)

12. [Dunne, J. *et al.* First dairying in green Saharan Africa in the fifth millennium BC. *Nature* **486**, 390–394 (2012).](http://paperpile.com/b/AGF0JC/bsKd)

13. [Biagetti, S. & di Lernia, S. Holocene Deposits of Saharan Rock Shelters: The Case of Takarkori and Other Sites from the Tadrart Acacus Mountains (Southwest Libya). *African Archaeological Review* **30**, 305–338 (2013).](http://paperpile.com/b/AGF0JC/X7Bm)

14. [Cherkinsky, A. & di Lernia, S. Bayesian approach to 14C dates for estimation of long-term archaeological sequences in arid environments: The Holocene site of Takarkori rockshelter, southwest Libya. *Radiocarbon* **55**, 771–782 (2013).](http://paperpile.com/b/AGF0JC/1h7J)

15. [Rotunno, R., Mercuri, A. M., Florenzano, A., Zerboni, A. & di Lernia, S. Coprolites from Rock Shelters: Hunter-Gatherers ‘Herding’ Barbary Sheep in the Early Holocene Sahara. *Journal of African Archaeology* **17**, 76–94 (2019).](http://paperpile.com/b/AGF0JC/QcLx)

16. [Rotunno, R., Cavorsi, L. & di Lernia, S. A Holocene Ceramic Sequence in the Central Sahara: Pottery Traditions and Social Dynamics Seen from the Takarkori Rockshelter (SW Libya). *African Archaeological Review* **40**, 647–672 (2023).](http://paperpile.com/b/AGF0JC/6CKi)

17. [di Lernia, S. Earliest Herders of the Central Sahara (Tadrart Acacus Mountains, Libya): A Punctuated Model for the Emergence of Pastoralism in Africa. *Journal of World Prehistory* **34**, 531–594 (2021).](http://paperpile.com/b/AGF0JC/0tGG)

18. [Lernia, S. di, di Lernia, S. & Tafuri, M. A. Persistent deathplaces and mobile landmarks: The Holocene mortuary and isotopic record from Wadi Takarkori (SW Libya). *Journal of Anthropological Archaeology* vol. 32 1–15 Preprint at https://doi.org/](http://paperpile.com/b/AGF0JC/0xmvE)[10.1016/j.jaa.2012.07.002](http://dx.doi.org/10.1016/j.jaa.2012.07.002) [(2013).](http://paperpile.com/b/AGF0JC/0xmvE)

19. [Tafuri, M. A., Bentley, R. A., Manzi, G. & di Lernia, S. Mobility and kinship in the prehistoric Sahara: Strontium isotope analysis of Holocene human skeletons from the Acacus Mts. (southwestern Libya). *Journal of Anthropological Archaeology* **25**, 390–402 (2006).](http://paperpile.com/b/AGF0JC/bNAT)

20. [di Lernia, S. *et al.* Inside the ‘African cattle complex’: animal burials in the holocene central Sahara. *PLoS One* **8**, e56879 (2013).](http://paperpile.com/b/AGF0JC/rQUC)

21. [di Lernia, S. & Gallinaro, M. *ATLAS of Tadrart Acacus Rock Art. A UNESCO World Heritage Site in Southwestern Libya*. (All’Insegna del Giglio, 2022).](http://paperpile.com/b/AGF0JC/JiTB)

22. [Ramsey, C. B. Bayesian Analysis of Radiocarbon Dates. *Radiocarbon* **51**, 337–360 (2009).](http://paperpile.com/b/AGF0JC/eNjj)

23. [Reimer, P. J. *et al.* The IntCal20 Northern Hemisphere Radiocarbon Age Calibration Curve (0–55 cal kBP). *Radiocarbon* **62**, 725–757 (2020).](http://paperpile.com/b/AGF0JC/9AsM)

24. [Vai, S. *et al.* Ancestral mitochondrial N lineage from the Neolithic ‘green’ Sahara. *Sci. Rep.* **9**, 3530 (2019).](http://paperpile.com/b/AGF0JC/68BW)

25. [Dabney, J. *et al.* Complete mitochondrial genome sequence of a Middle Pleistocene cave bear reconstructed from ultrashort DNA fragments. *Proc. Natl. Acad. Sci. U. S. A.* **110**, 15758–15763 (2013).](http://paperpile.com/b/AGF0JC/x6t8G)

26. [Meyer, M. & Kircher, M. Illumina sequencing library preparation for highly multiplexed target capture and sequencing. *Cold Spring Harb. Protoc.* **2010**, db.prot5448 (2010).](http://paperpile.com/b/AGF0JC/wmL4Q)

27. [Gansauge, M.-T. & Meyer, M. Selective enrichment of damaged DNA molecules for ancient genome sequencing. *Genome Res.* **24**, 1543–1549 (2014).](http://paperpile.com/b/AGF0JC/1rhf6)

28. [Gansauge, M.-T. *et al.* Single-stranded DNA library preparation from highly degraded DNA using T4 DNA ligase. *Nucleic Acids Res.* **45**, e79 (2017).](http://paperpile.com/b/AGF0JC/EDWGA)

29. [Peltzer, A. *et al.* EAGER: efficient ancient genome reconstruction. *Genome Biol.* **17**, 60 (2016).](http://paperpile.com/b/AGF0JC/3oZyA)

30. [Mathieson, I. *et al.* Genome-wide patterns of selection in 230 ancient Eurasians. *Nature* **528**, 499–503 (2015).](http://paperpile.com/b/AGF0JC/ACGf)

31. [Rohland, N. *et al.* Three assays for in-solution enrichment of ancient human DNA at more than a million SNPs. *Genome Res.* **32**, 2068–2078 (2022).](http://paperpile.com/b/AGF0JC/eUQg)

32. [Fu, Q. *et al.* A revised timescale for human evolution based on ancient mitochondrial genomes. *Curr. Biol.* **23**, 553–559 (2013).](http://paperpile.com/b/AGF0JC/oH7eX)

33. [Li, H. & Durbin, R. Fast and accurate short read alignment with Burrows–Wheeler transform. *Bioinformatics* **25**, 1754–1760 (2009).](http://paperpile.com/b/AGF0JC/XM78f)

34. [Schubert, M., Lindgreen, S. & Orlando, L. AdapterRemoval v2: rapid adapter trimming, identification, and read merging. *BMC Res. Notes* **9**, 88 (2016).](http://paperpile.com/b/AGF0JC/DK0o)

35. [Neukamm, J., Peltzer, A. & Nieselt, K. DamageProfiler: fast damage pattern calculation for ancient DNA. *Bioinformatics* **37**, 3652–3653 (2021).](http://paperpile.com/b/AGF0JC/m1s4)

36. [de Filippo, C., Meyer, M. & Prüfer, K. Quantifying and reducing spurious alignments for the analysis of ultra-short ancient DNA sequences. *BMC Biol.* **16**, 121 (2018).](http://paperpile.com/b/AGF0JC/n4sZ)

37. [Peyrégne, S. & Peter, B. M. AuthentiCT: a model of ancient DNA damage to estimate the proportion of present-day DNA contamination. *Genome Biol.* **21**, 246 (2020).](http://paperpile.com/b/AGF0JC/Snl0k)

38. [Posth, C. *et al.* Palaeogenomics of Upper Palaeolithic to Neolithic European hunter-gatherers. *Nature* **615**, 117–126 (2023).](http://paperpile.com/b/AGF0JC/Nutc)

39. [Skoglund, P. *et al.* Separating endogenous ancient DNA from modern day contamination in a Siberian Neandertal. *Proc. Natl. Acad. Sci. U. S. A.* **111**, 2229–2234 (2014).](http://paperpile.com/b/AGF0JC/P4f5U)

40. [Mallick, S. *et al.* The Allen Ancient DNA Resource (AADR): A curated compendium of ancient human genomes. *bioRxiv* 2023.04.06.535797 (2023) doi:](http://paperpile.com/b/AGF0JC/5Zb2)[10.1101/2023.04.06.535797](http://dx.doi.org/10.1101/2023.04.06.535797)[.](http://paperpile.com/b/AGF0JC/5Zb2)

41. [Fan, S. *et al.* African evolutionary history inferred from whole genome sequence data of 44 indigenous African populations. *Genome Biol.* **20**, 82 (2019).](http://paperpile.com/b/AGF0JC/WM0m)

42. [Lazaridis, I. *et al.* Ancient human genomes suggest three ancestral populations for present-day Europeans. *Nature* **513**, 409–413 (2014).](http://paperpile.com/b/AGF0JC/PuJPM)

43. [Pickrell, J. K. *et al.* The genetic prehistory of southern Africa. *Nat. Commun.* **3**, 1143 (2012).](http://paperpile.com/b/AGF0JC/tur2)

44. [D’Atanasio, E. *et al.* The genomic echoes of the last Green Sahara on the Fulani and Sahelian people. *Curr. Biol.* **33**, 5495–5504.e4 (2023).](http://paperpile.com/b/AGF0JC/4zFs)

45. [Simões, L. G. *et al.* Northwest African Neolithic initiated by migrants from Iberia and Levant. *Nature* (2023) doi:](http://paperpile.com/b/AGF0JC/mk0Ju)[10.1038/s41586-023-06166-6](http://dx.doi.org/10.1038/s41586-023-06166-6)[.](http://paperpile.com/b/AGF0JC/mk0Ju)

46. [Lucas-Sánchez, M., Fadhlaoui-Zid, K. & Comas, D. The genomic analysis of current-day North African populations reveals the existence of trans-Saharan migrations with different origins and dates. *Hum. Genet.* **142**, 305–320 (2023).](http://paperpile.com/b/AGF0JC/6Loh)

47. [Fortes-Lima, C. *et al.* Demographic and selection histories of populations across the Sahel/Savannah belt. *Mol. Biol. Evol.* **39**, (2022).](http://paperpile.com/b/AGF0JC/MuoE)

48. [Patterson, N. *et al.* Ancient admixture in human history. *Genetics* **192**, 1065–1093 (2012).](http://paperpile.com/b/AGF0JC/JjjJ)

49. [Peter, B. M., Petkova, D. & Novembre, J. Genetic landscapes reveal how human genetic diversity aligns with geography. *Mol. Biol. Evol.* **37**, 943–951 (2020).](http://paperpile.com/b/AGF0JC/iJ7E)

50. [Pugach, I. & Stoneking, M. Genome-wide insights into the genetic history of human populations. *Investig. Genet.* **6**, 6 (2015).](http://paperpile.com/b/AGF0JC/53Lk)

51. [Lazaridis, I. *et al.* Genomic insights into the origin of farming in the ancient Near East. *Nature* **536**, 419–424 (2016).](http://paperpile.com/b/AGF0JC/VxTvs)

52. [Maier, R. *et al.* On the limits of fitting complex models of population history to f-statistics. *Elife* **12**, (2023).](http://paperpile.com/b/AGF0JC/4RDtT)

53. [Flegontov, P. *et al.* Modeling of African population history using f-statistics is biased when applying all previously proposed SNP ascertainment schemes. *PLoS Genet.* **19**, e1010931 (2023).](http://paperpile.com/b/AGF0JC/wtT3)

54. [Narasimhan, V. M. *et al.* The formation of human populations in South and Central Asia. *Science* **365**, (2019).](http://paperpile.com/b/AGF0JC/32GQ)

55. [Ringbauer, H., Novembre, J. & Steinrücken, M. Parental relatedness through time revealed by runs of homozygosity in ancient DNA. *Nat. Commun.* **12**, 5425 (2021).](http://paperpile.com/b/AGF0JC/GhPx)

56. [Alexander, D. H., Novembre, J. & Lange, K. Fast model-based estimation of ancestry in unrelated individuals. *Genome Res.* **19**, 1655–1664 (2009).](http://paperpile.com/b/AGF0JC/gtzp)

57. [Schmid, C. *et al.* Poseidon – A framework for archaeogenetic human genotype data management. *eLife* (2024) doi:](http://paperpile.com/b/AGF0JC/HBm1)[10.7554/elife.98317.1](http://dx.doi.org/10.7554/elife.98317.1)[.](http://paperpile.com/b/AGF0JC/HBm1)

58. [Chang, C. C. *et al.* Second-generation PLINK: rising to the challenge of larger and richer datasets. *Gigascience* **4**, 7 (2015).](http://paperpile.com/b/AGF0JC/QTvI)

59. [Peter, B. M. 100,000 years of gene flow between Neandertals and Denisovans in the Altai mountains. *bioRxiv* 2020.03.13.990523 (2020) doi:](http://paperpile.com/b/AGF0JC/0ACkh)[10.1101/2020.03.13.990523](http://dx.doi.org/10.1101/2020.03.13.990523)[.](http://paperpile.com/b/AGF0JC/0ACkh)

60. [van de Loosdrecht, M. *et al.* Pleistocene North African genomes link Near Eastern and sub-Saharan African human populations. *Science* **360**, 548–552 (2018).](http://paperpile.com/b/AGF0JC/L1AMf)

61. [Fu, Q. *et al.* An early modern human from Romania with a recent Neanderthal ancestor. *Nature* **524**, 216–219 (2015).](http://paperpile.com/b/AGF0JC/YipHZ)

62. [Lipson, M. *et al.* Ancient West African foragers in the context of African population history. *Nature* **577**, 665–670 (2020).](http://paperpile.com/b/AGF0JC/SlHRu)

63. [Jones, E. R. *et al.* Upper Palaeolithic genomes reveal deep roots of modern Eurasians. *Nat. Commun.* **6**, 8912 (2015).](http://paperpile.com/b/AGF0JC/uFsyu)

64. [Wohns, A. W. *et al.* A unified genealogy of modern and ancient genomes. *Science* **375**, eabi8264 (2022).](http://paperpile.com/b/AGF0JC/ADGUf)

65. [Rivollat, M. *et al.* Ancient genome-wide DNA from France highlights the complexity of interactions between Mesolithic hunter-gatherers and Neolithic farmers. *Sci Adv* **6**, eaaz5344 (2020).](http://paperpile.com/b/AGF0JC/zFdXo)

66. [Prüfer, K. *et al.* The complete genome sequence of a Neanderthal from the Altai Mountains. *Nature* **505**, 43–49 (2014).](http://paperpile.com/b/AGF0JC/PK23O)

67. [Prüfer, K. *et al.* A high-coverage Neandertal genome from Vindija Cave in Croatia. *Science* **358**, 655–658 (2017).](http://paperpile.com/b/AGF0JC/KfGZD)

68. [Mafessoni, F. *et al.* A high-coverage Neandertal genome from Chagyrskaya Cave. *Proc. Natl. Acad. Sci. U. S. A.* **117**, 15132–15136 (2020).](http://paperpile.com/b/AGF0JC/QkkT1)

69. [Meyer, M. *et al.* A high-coverage genome sequence from an archaic Denisovan individual. *Science* **338**, 222–226 (2012).](http://paperpile.com/b/AGF0JC/4OGDo)

70. [1000 Genomes Project Consortium *et al.* A global reference for human genetic variation. *Nature* **526**, 68–74 (2015).](http://paperpile.com/b/AGF0JC/JzVfK)

71. [Sankararaman, S., Mallick, S., Patterson, N. & Reich, D. The Combined Landscape of Denisovan and Neanderthal Ancestry in Present-Day Humans. *Curr. Biol.* **26**, 1241–1247 (2016).](http://paperpile.com/b/AGF0JC/6aRTU)

72. [Sankararaman, S. *et al.* The genomic landscape of Neanderthal ancestry in present-day humans. *Nature* **507**, 354–357 (2014).](http://paperpile.com/b/AGF0JC/9XYOa)

73. [Aston, J. A. D. & Martin, D. E. K. Distributions associated with general runs and patterns in hidden Markov models. *aoas* **1**, 585–611 (2007).](http://paperpile.com/b/AGF0JC/aTO7H)

74. [Durbin, R., Eddy, S., Krogh, A. & Mitchison, G. *Biological Sequence Analysis: Probabalistic Models of Proteins and Nucleic Acids*. (Cambridge University Press, 2013).](http://paperpile.com/b/AGF0JC/8Z0w9)

75. [Prüfer, K. *et al.* A genome sequence from a modern human skull over 45,000 years old from Zlatý kůň in Czechia. *Nat Ecol Evol* **5**, 820–825 (2021).](http://paperpile.com/b/AGF0JC/sqlaS)

76. [Drummond, A. J., Suchard, M. A., Xie, D. & Rambaut, A. Bayesian phylogenetics with BEAUti and the BEAST 1.7. *Mol. Biol. Evol.* **29**, 1969–1973 (2012).](http://paperpile.com/b/AGF0JC/wlHl)

77. [Bouckaert, R. *et al.* BEAST 2.5: An advanced software platform for Bayesian evolutionary analysis. *PLoS Comput. Biol.* **15**, e1006650 (2019).](http://paperpile.com/b/AGF0JC/9vOql)

78. [Schlebusch, C. M. *et al.* Southern African ancient genomes estimate modern human divergence to 350,000 to 260,000 years ago. *Science* **358**, 652–655 (2017).](http://paperpile.com/b/AGF0JC/0S2AU)

79. [Skoglund, P. *et al.* Reconstructing Prehistoric African Population Structure. *Cell* **171**, 59–71.e21 (2017).](http://paperpile.com/b/AGF0JC/2Ziet)

80. [Hublin, J.-J. *et al.* Initial Upper Palaeolithic Homo sapiens from Bacho Kiro Cave, Bulgaria. *Nature* **581**, 299–302 (2020).](http://paperpile.com/b/AGF0JC/nfV6t)

81. [Hajdinjak, M. *et al.* Initial Upper Palaeolithic humans in Europe had recent Neanderthal ancestry. *Nature* **592**, 253–257 (2021).](http://paperpile.com/b/AGF0JC/IUO6U)

82. [Devièse, T. *et al.* Compound-specific radiocarbon dating and mitochondrial DNA analysis of the Pleistocene hominin from Salkhit Mongolia. *Nat. Commun.* **10**, 274 (2019).](http://paperpile.com/b/AGF0JC/VH3gd)

83. [Posth, C. *et al.* Pleistocene Mitochondrial Genomes Suggest a Single Major Dispersal of Non-Africans and a Late Glacial Population Turnover in Europe. *Curr. Biol.* **26**, 827–833 (2016).](http://paperpile.com/b/AGF0JC/3kUVj)

84. [Posth, C. *et al.* Deeply divergent archaic mitochondrial genome provides lower time boundary for African gene flow into Neanderthals. *Nat. Commun.* **8**, 16046 (2017).](http://paperpile.com/b/AGF0JC/1DOex)

85. [Pugach, I. *et al.* Ancient DNA from Guam and the peopling of the Pacific. *Proc. Natl. Acad. Sci. U. S. A.* **118**, (2021).](http://paperpile.com/b/AGF0JC/ZscSS)

86. [Prüfer, K. snpAD: an ancient DNA genotype caller. *Bioinformatics* **34**, 4165–4171 (2018).](http://paperpile.com/b/AGF0JC/HdOaS)

87. [Weissensteiner, H. *et al.* HaploGrep 2: mitochondrial haplogroup classification in the era of high-throughput sequencing. *Nucleic Acids Res.* **44**, W58–63 (2016).](http://paperpile.com/b/AGF0JC/yUaBe)

88. [Katoh, K. & Standley, D. M. MAFFT multiple sequence alignment software version 7: improvements in performance and usability. *Mol. Biol. Evol.* **30**, 772–780 (2013).](http://paperpile.com/b/AGF0JC/DwaGZ)

89. [Bouckaert, R. R. & Drummond, A. J. bModelTest: Bayesian phylogenetic site model averaging and model comparison. *BMC Evol. Biol.* **17**, 42 (2017).](http://paperpile.com/b/AGF0JC/HzowK)

90. [Kass, R. E. & Raftery, A. E. Bayes Factors. *J. Am. Stat. Assoc.* **90**, 773–795 (1995).](http://paperpile.com/b/AGF0JC/gDRNN)

# Supplementary Note 3: Non-peer reviewed abstract in Arabic

**جينومات بشرية من الصحراء الخضراء تكشف عن سلالة شمال أفريقية أصلية**

تُعرف اليوم الصحراء الكبرى بأنها واحدة من أكثر المناطق جفافاً على كوكب الأرض، لكنها كانت عبارة عن سافانا خضراء خلال الفترة الرطبة الأفريقية بين 14500 و5000 عام قبل الحاضر، وكانت تتخللها المسطحات المائية التي شجعت النشاط البشري وانتشار الرعي خلال الهولوسين الأوسط. نظرًا للظروف الحارة والجافة، فإن الحمض النووي لا يُحفظ غالباً بشكل جيد في هذه المنطقة، مما يؤدي إلى معرفة محدودة بتاريخ سكان الصحراء وماضيهم الديموغرافي. في هذه الدراسة، نقدم أول جينومات بشرية قديمة من الصحراء الكبرى تعود للعصر الحجري الحديث الرعوي، ويُقدر عمرها بـ 7000 عام. تعود هذه الجينومات إلى إناث وُجدن مدفونات في ملجأ تاكاركوري الصخري بجنوب غرب ليبيا، والذي كان يُستخدم كمدفن من قبل المجتمعات الرعوية.

وجدنا أن غالبية أسلاف أفراد تاكاركوري ينتمون إلى سلالة وراثية شمال أفريقية لم تكن معروفة سابقًا، والتي يبدو أنها ظلت منعزلة طوال معظم فترة وجودها، وتختلف عن سلالات أفريقيا جنوب الصحراء الكبرى وعن المجموعات الأخرى خارج أفريقيا التي تواجدت في نفس الفترة الزمنية. يرتبط أفراد موقع تاكاركوري ارتباطًا وثيقًا بالصيادين الجامعين الذين عاشوا قبل حوالي 15000 عام - وهي الفترة التي سبقت الفترة الرطبة الأفريقية بقليل - والذين سكنوا مغارة تافوغالت في المغرب وامتهنوا الصناعات الحجرية الإيبيرومورية. يُظهر التحليل أيضاً أن كلًا من تاكاركوري والأفراد الإيبيروموريين يرتبطون بشكل متساوٍ بسلالات أفريقيا جنوب الصحراء الكبرى، مما يشير إلى تدفق جيني محدود من جنوب الصحراء الكبرى إلى شمال أفريقيا خلال الفترة الرطبة الأفريقية.

على عكس مجموعات تافوغالت، التي تُظهر اختلاطًا مع النياندرتال بنسبة أقل بمرتين مقارنةً بالجينومات الموجودة خارج أفريقيا، تظهر جينومات أفراد تاكاركوري أصولًا نياندرتالية بنسبة أقل بعشر مرات مقارنةً بالمزارعين في منطقة المشرق. ومع ذلك، تظل هذه النسبة أعلى بشكل ملحوظ مقارنةً بالجينومات المعاصرة في أفريقيا جنوب الصحراء الكبرى. تدعم نتائجنا نموذجًا يُظهر أن انتشار النشاط الرعوي في هذه السلالة الشمالية الأفريقية المنعزلة كان نتيجة لاكتساب ثقافي أكثر من كونه نتيجة لتداخل جيني، مما يشير إلى أن الممارسات الرعوية انتشرت على نطاق واسع في شمال أفريقيا خلال أواخر العصر البلايستوسيني دون تدفق جيني كبير من مناطق أخرى.

1. This and the following calibrated date are obtained according to OxCal online version 4.4[^22^](https://paperpile.com/c/AGF0JC/eNjj), and calibration curve IntCal 20[^23^](https://paperpile.com/c/AGF0JC/9AsM). [↑](#footnote-ref-1)
