# Supplementary material for: Ancient DNA from the Green Sahara reveals ancestral North African lineage
Source: Nature. 2025 Apr 2;641(8061):144–50. doi: 10.1038/s41586-025-08793-7 (PMC12043513; doi:10.1038/s41586-025-08793-7)
Supplement: Supplementary file 2 — Reporting Summary [file 41586_2025_8793_MOESM2_ESM.pdf]

nature portfolio

Corresponding author(s): Nada Salem, Harald Rignbauer, David Caramelli, Savino di Lernia, Johannes Krause (2024-01-00029C)

Last updated by author(s): Jan 15, 2025

Reporting Summary

Nature Portfolio wishes to improve the reproducibility of the work that we publish. This form provides structure for consistency and transparency in reporting. For further information on Nature Portfolio policies, see our Editorial Policies and the Editorial Policy Checklist.

Statistics

For all statistical analyses, confirm that the following items are present in the figure legend, table legend, main text, or Methods section.

n/a	Confirmed	
☐	☒	The exact sample size (*n*) for each experimental group/condition, given as a discrete number and unit of measurement
☐	☒	A statement on whether measurements were taken from distinct samples or whether the same sample was measured repeatedly
☐	☒	The statistical test(s) used AND whether they are one- or two-sided<br>*Only common tests should be described solely by name; describe more complex techniques in the Methods section.*
☒	☐	A description of all covariates tested
☐	☒	A description of any assumptions or corrections, such as tests of normality and adjustment for multiple comparisons
☐	☒	A full description of the statistical parameters including central tendency (e.g. means) or other basic estimates (e.g. regression coefficient) AND variation (e.g. standard deviation) or associated estimates of uncertainty (e.g. confidence intervals)
☐	☒	For null hypothesis testing, the test statistic (e.g. *F*, *t*, *r*) with confidence intervals, effect sizes, degrees of freedom and *P* value noted<br>*Give P values as exact values whenever suitable.*
☐	☒	For Bayesian analysis, information on the choice of priors and Markov chain Monte Carlo settings
☒	☐	For hierarchical and complex designs, identification of the appropriate level for tests and full reporting of outcomes
☒	☐	Estimates of effect sizes (e.g. Cohen's *d*, Pearson's *r*), indicating how they were calculated

Our web collection on statistics for biologists contains articles on many of the points above.

Software and code

Policy information about availability of computer code

Data collection	No specific software was used for data collection. All software used for the processing of raw sequencing data and generation of genotype files are listed below.
Data analysis	The following freely available software was used for data analyses. The corresponding citations are provided in the Methods or in the Supplementary section: AdapterRemoval (v.2.3.1), Burrows-Wheeler Aligner (v.0.7.12), DeDup (v.0.12.1), EAGER (v.1.92.56), SpAl (https://bioinf.eva.mpg.de/SpAl/), DamageProfiler (v.1.1), AuthentiCT (v.1.0), hapCon_ROH (https://haproh.readthedocs.io/en/latest/hapROH_with_contamination.html), PMDtools (v.0.6), pileupCaller (v.1.4.0.2), samtools (v.1.3), smartpca (v.16000; EIGENSOFT v.8.0), qp3Pop (v.435; ADMIXTOOLS v.5.1), qpDstat (v.755; ADMIXTOOLS v.5.1), qpAdm (v.810), admixfrog (v.0.7.1), admixtools (v.2.0.0), MAFFT (v.7.508), mitoBench-ancientMT (https://github.com/alexhbnr/mitoBench-ancientMT), snpAD (v.0.3.9), HaploGrep2 (v.2.1.19), BEAST, LogCombiner and TreeAnnotator (v.2.6.7), bModelTest (v.1.2.1). DATES (v.753),hapROH (v.3.0), ADMIXTURE (v.1.3.0), PLINK (v.1.9). Data visualization was performed in RStudio (v.2022.12.0+353). The following R packages were used for visualization: cowplot (v.1.1.2), ggplot (v.3.4.2), ggh4x (v.0.2.3), ggnewscale (v.0.4.8), janno (v.1.0.0), magrittr (v.2.0.3), maps (v.3.4.1), patchwork (v.1.1.2), purrr (v.1.0.1), RColorBrewer (v.1.1.3), readxl (v.1.4.1), tidyr (v.1.3.0), tidyverse (v.1.3.2).

For manuscripts utilizing custom algorithms or software that are central to the research but not yet described in published literature, software must be made available to editors and reviewers. We strongly encourage code deposition in a community repository (e.g. GitHub). See the Nature Portfolio guidelines for submitting code & software for further information.

Data

Policy information about availability of data

All manuscripts must include a data availability statement. This statement should provide the following information, where applicable:

- Accession codes, unique identifiers, or web links for publicly available datasets
- A description of any restrictions on data availability
- For clinical datasets or third party data, please ensure that the statement adheres to our policy

The published genotype data, compiled and annotated as the Allen Ancient DNA Resource (AADR versions 51.1 and 54.1), was used for comparative analyses and is available here: https://reich.hms.harvard.edu/allen-ancient-dna-resource-aadr-downloadable-genotypes-present-day-and-ancient-dna-data. Additionally, previously published data from Pickrell et al. (2012), Lazaridis et al. (2014) and Fortes-Lima et al. (2022) were included. Present-day data from D'Atanasio et al. (2023) and Lucas-Sanchez et al. (2023) were obtained with permission. High-quality ancient shotgun sequencing data and present-day sub-Saharan genomes were sourced from the Allen Ancient Genome Diversity Project/John Templeton Ancient DNA Atlas (https://reich.hms.harvard.edu/ancient-genome-diversity-project).

Research involving human participants, their data, or biological material

Policy information about studies with human participants or human data. See also policy information about sex, gender (identity/presentation), and sexual orientation and race, ethnicity and racism.

Reporting on sex and gender	We reported the biological sex of the individuals. Identifying self-assigned gender is not possible from aDNA data.
Reporting on race, ethnicity, or other socially relevant groupings	For some analyses, we grouped samples based on geography, time period, and followed standard criteria. These groupings have been previously used in aDNA studies to increase statistical power, and are based on genetic affinity as well as archaeological and cultural context.
Population characteristics	NA
Recruitment	NA
Ethics oversight	NA

Note that full information on the approval of the study protocol must also be provided in the manuscript.

Field-specific reporting

Please select the one below that is the best fit for your research. If you are not sure, read the appropriate sections before making your selection.

☒ Life sciences ☐ Behavioural & social sciences ☐ Ecological, evolutionary & environmental sciences

For a reference copy of the document with all sections, see nature.com/documents/nr-reporting-summary-flat.pdf

Life sciences study design

All studies must disclose on these points even when the disclosure is negative.

Sample size	Genomic data from two ancient individuals from Libya were analyzed in this study. The sample size depended on the availability of human remains dating back to the Prehistoric period from the present-day Sahara region that had preserved and retrievable ancient DNA sequences. Specimens from this period and region are very rare due to the poor molecular preservation of human remains. Despite this, the analysis of millions of genetic variants in each individual provides valuable information about their genetic history.
Data exclusions	Reads shorter than 30 base pairs (bp), with more than 10% mismatch from the Reference genome and mapping quality score below 25 were discarded while preparing bamfiles for merged genomic libraries data.
Replication	Replication was successfully achieved by comparing the results obtained from both the shotgun and nuclear capture datasets, as well as between the single-stranded and double-stranded sequencing data.
Randomization	Randomization is not applicable to this study. The two samples were grouped according to the archaeological site of origin and radiocarbon date (pastoralism period).
Blinding	Blinding is not applicable in this study. The archaeological context for the two individuals analyzed, including the site location and estimated date, was known before sampling and analysis. This prior knowledge is essential for the conceptualization of the study.

Reporting for specific materials, systems and methods

We require information from authors about some types of materials, experimental systems and methods used in many studies. Here, indicate whether each material, system or method listed is relevant to your study. If you are not sure if a list item applies to your research, read the appropriate section before selecting a response.

Materials & experimental systems

n/a	Involved in the study
☒	☐ Antibodies
☒	☐ Eukaryotic cell lines
☐	☒ Palaeontology and archaeology
☒	☐ Animals and other organisms
☒	☐ Clinical data
☒	☐ Dual use research of concern
☒	☐ Plants

Methods

n/a	Involved in the study
☒	☐ ChIP-seq
☒	☐ Flow cytometry
☒	☐ MRI-based neuroimaging

Palaeontology and Archaeology

Specimen provenance: Archaeological samples were excavated in Libya between 2004 and 2006 at the Takarkori rock shelter, located in the Tadrart Acacus mountain range near the Algerian border. The excavation and handling of human remains were extensively discussed with the Department of Antiquities (DoA) in Tripoli, Libya (formerly the Socialist People's Libyan Arab Jamahiriya), particularly regarding ethical considerations.

Specimen deposition: The human remains are curated at the Museum of Anthropology of the University of Rome, La Sapienza.

Dating methods: *If new dates are provided, describe how they were obtained (e.g. collection, storage, sample pretreatment and measurement), where they were obtained (i.e. lab name), the calibration program and the protocol for quality assurance OR state that no new dates are provided.*

☒ Tick this box to confirm that the raw and calibrated dates are available in the paper or in Supplementary Information.

Ethics oversight: Permits for sampling and analysis of the archaeological material were obtained from the DoA. Approval for the excavation was granted on 28/01/2004, under reference number: ه ٩٨ – ١٠٠٠٠ ط ع ث م ٤٩٤٦٣ (translated as H98-10000 TATM 49463). Ethical considerations, including local community involvement and collaboration with the DoA, were discussed in the 'Inclusion and ethics' section of the manuscript.

Note that full information on the approval of the study protocol must also be provided in the manuscript.

Plants

Seed stocks: *Report on the source of all seed stocks or other plant material used. If applicable, state the seed stock centre and catalogue number. If plant specimens were collected from the field, describe the collection location, date and sampling procedures.*

Novel plant genotypes: *Describe the methods by which all novel plant genotypes were produced. This includes those generated by transgenic approaches, gene editing, chemical/radiation-based mutagenesis and hybridization. For transgenic lines, describe the transformation method, the number of independent lines analyzed and the generation upon which experiments were performed. For gene-edited lines, describe the editor used, the endogenous sequence targeted for editing, the targeting guide RNA sequence (if applicable) and how the editor was applied.*

Authentication: *Describe any authentication procedures for each seed stock used or novel genotype generated. Describe any experiments used to assess the effect of a mutation and, where applicable, how potential secondary effects (e.g. second site T-DNA insertions, mosiacism, off-target gene editing) were examined.*
